# Taking Care of the Puerto Rican Patient: Historical Perspectives, Health Status, and Health Care Access

**DOI:** 10.15766/mep_2374-8265.10984

**Published:** 2020-10-07

**Authors:** Débora H. Silva Díaz, Glenn Garcia, Camille Clare, Julia Su, Erica Friedman, Renee Williams, Juan Vazquez, John Paul Sánchez

**Affiliations:** 1 Professor, Department of Pediatrics, University of Puerto Rico School of Medicine; 2 Medical Student, Rutgers New Jersey Medical School; 3 Associate Professor, Department of Obstetrics and Gynecology, Associate Dean of Diversity and Inclusion, New York Medical College; 4 MD/PhD Candidate, Donald and Barbara Zucker School of Medicine at Hofstra/Northwell, National President, Latino Medical Student Association Inc.; 5 Interim Dean, CUNY School of Medicine; 6 Fellowship Director, Adult Gastroenterology and Hepatology, NYU Grossman School of Medicine; 7 Medical Student, Albert Einstein College of Medicine; 8 Associate Professor, Department of Emergency Medicine, Rutgers New Jersey Medical School

**Keywords:** Puerto Rican, Hispanic, Cultural Competence, Health Care Disparities, Case Discussion, Diversity, Inclusion, Health Equity

## Abstract

**Introduction:**

Hispanics are the largest minority group in the US at 18% of the population, of which Puerto Ricans are the second largest subgroup. Puerto Ricans have poorer health status than other US Hispanic and non-Hispanic populations. Thus, health care providers need to know about and distinguish the health care problems of Puerto Ricans to improve their health. Although there are some published curricula addressing how to provide health care to Hispanic populations, none address the specific needs of Puerto Ricans.

**Methods:**

We developed a 60-minute interactive workshop consisting of a PowerPoint presentation and case discussion aimed at increasing health care providers' knowledge and understanding of the historical perspective that led to Puerto Rican identity, health issues and disparities, and the health care access problems of mainland and islander Puerto Ricans. Evaluation consisted of pre- and postworkshop questionnaires.

**Results:**

There were a total of 64 participants with diverse ethnoracial identities including medical students, residents, faculty, physicians, researchers, administrators, and students/faculty from nursing, occupational therapy, genetic counseling, biomedical sciences, and social work programs. A comparison of pre- and postworkshop data showed a statistically significant increase in participants' confidence in meeting all learning objectives. Participants positively commented on the interactive nature of the workshop, the case discussion, and the historical perspective provided.

**Discussion:**

With the increasing migration of Puerto Ricans to the US mainland this module can uniquely improve the preparation of current and future health care providers to provide competent care to Puerto Rican patients.

## Educational Objectives

By the end of this activity, learners will be able to:

1. Describe the history of Puerto Rican identity within the United States.
2. Compare and contrast health issues and disparities of Puerto Ricans residing on the island and on the mainland.
3. Explain how at least one state or federal policy has impacted the health outcomes of Puerto Ricans on the mainland and/or the island.
4. Identify at least two health care access problems faced by Puerto Ricans on the mainland and/or the island.

## Introduction

Hispanics are the largest minority group in the US making up 18% of the population with an estimated rise to 23% by 2035.^1,2^ With this projected rise, recognizing and addressing the social determinants of health within the various Hispanic communities is of increasing importance for all health care professionals. From the total Hispanic population, Puerto Ricans are the second largest group with around 5.5 million living on mainland US, and comprising 9.5% of the Hispanic population.^2,3^ Thus health care providers need to know the health status of Puerto Ricans on the island and on the mainland to address health disparities and improve their health.

Puerto Ricans on the mainland report a lack of adequate access to health care, poorer health status, more chronic illnesses, worse psychological distress, and lower life span than other Hispanic and non-Hispanic populations in the US.^3–6^ Contributory factors include higher rates of diabetes, heart disease, and asthma.^7–8^ Puerto Ricans on the island suffer even worse health outcomes, for example, a 50% higher prevalence and three times the death rate from diabetes than those living on the mainland and in comparison to other Hispanic and non-Hispanic populations.^9^ Large-scale migrations in the last decade bring these health concerns of Puerto Ricans on the island to the mainland US.^10,11^ In 2017 alone, following the devastation of Hurricane Maria, over 300,000 Puerto Ricans migrated from the island to the mainland.^12^

Although there are some published curricula addressing how to provide health care to Hispanic populations with emphasis on communication skills and medical Spanish, there are none that addressed the specific health needs of Puerto Ricans.^13–15^ In addition, *MedEdPORTAL* has no curricula specific to the social determinants of health care in Puerto Rico, Puerto Rican communities, or under the umbrella of the Hispanic community. This workshop presents a unique topic of increasing relevance given the large number of Puerto Ricans living or relocating to mainland United States. Of note, three of the coauthors of this workshop are Puerto Ricans who have lived and worked with Puerto Rican populations both on the mainland and on the island. Combined, three of the coauthors have significant experience in providing education on health care disparities, competent care, and the development of curricula for medically underserved populations. These faculty members and students are uniquely positioned to advocate for educational initiatives, such as this workshop, to be included in medical school curricula, in order to improve medical education for all health care providers.

This workshop was developed by following the six-step approach to curriculum development by Kern and colleagues.^16^ In steps 1 and 2, problem identification and general needs assessment and targeted needs assessment, we conducted a critical literature review focusing on Puerto Rican history and identity formation; Puerto Rican health statistics, health care problems, and health care access; and training resources available for health care providers specific to Hispanic populations. This module may also assist medical schools and residency programs in meeting Liaison Committee on Medical Education Elements (LCME) 3.3 Diversity/Pipeline Programs and Partnerships as well as 7.6 Cultural Competence and Health Care Disparities; and ACGME Interpersonal and Communication Skills Competence (regarding effective communication skills with patients and families of different backgrounds) and the new diversity standard effective July 1, 2019 (ensuring a diverse workforce for a diverse patient population).^17,18^

For LCME step 3, goals and objectives were developed from the literature review and coauthors' suggestions. For step 4, educational strategies, we chose an interactive workshop using a PowerPoint presentation, case discussion, and reflection exercises. The Institutional Review Board (IRB) at Rutgers New Jersey Medical School approved the evaluation method. In step 5, the workshop was implemented at various institutions as part of their Hispanic Heritage Month celebrations for health care professionals and trainees in New York City. For step 6, evaluation and feedback, a questionnaire was developed based on the PowerPoint presentation's learning objectives and content in order for participants to assess the attainment of the delineated workshop objectives. Participant's pre- and postworkshop questionnaires were used in the evaluation and revision of the workshop.

Although the workshop was developed specifically with medical students, residents, and physicians in mind, it is also appropriate for all other health professions and prehealth trainees. Thus, this curriculum can be for use in all health professional training programs by any faculty member that prepares for it. The purpose is to train future health professionals about the social determinants of health of the Puerto Rican community, with special emphasis on medical conditions. The long-term goal is that implementing workshops like this one will improve the care provided to Puerto Rican patients. It addresses specific knowledge related to the history of Puerto Rican identity formation, and the health status and health care access issues of Puerto Ricans living on the island and the mainland. It also describes the underlying causes of health care disparities between Puerto Ricans living on the mainland and other Hispanic and non-Hispanic populations. The case discussion and reflection exercises provide an avenue to contextualize the newly acquired knowledge about the health status of the Puerto Rican population. Final recommendations are provided for improving the health of the Puerto Rican population, thus adding a unique curricular component to health care literature related to the care of Hispanic populations.

## Methods

The workshop was developed, implemented, and evaluated by a team of experts in Puerto Rican history, Puerto Rican health, health care delivery to the Puerto Rican population both on the island and on the mainland, and with extensive experience in teaching and curricula development on health disparities and the care of patients across a broad range of backgrounds. The development team included a physician and educator with significant experience providing clinical care to underserved populations in Puerto Rico, developing curricula, and teaching; a physician with ample experience doing research and developing curricula in health disparities; and a medical student who has taught Puerto Rican history and has published his work on health care disparities. Implementation and evaluation of the workshop was done by all authors. The workshop was piloted at a *MedEdPORTAL* training which took place at the Universidad Central del Caribe College of Medicine in Puerto Rico. This training was attended by faculty and students from the four medical schools in Puerto Rico. The results were used to make improvements to the workshop before implementing it at New York medical schools.

The workshop was implemented seven times at seven different medical schools in the state of New York (New York City Health + Hospitals/Metropolitan, an educational clinical affiliate of New York Medical College, Albert Einstein College of Medicine, CUNY School of Medicine, NYU Grossman School of Medicine, SUNY Downstate College of Medicine, Vagelos College of Physicians and Surgeons of Columbia University, and Donald and Barbara Zucker School of Medicine at Hofstra/Northwell). Because this was an educational study that included students and residents, IRB approval was necessary for publishing purposes. IRB approval was received at five different sites, and our results included only the data obtained from those institutions. Evaluation of the workshop served for its final edits.

This 60-minute interactive workshop aimed to increase health care providers' confidence and understanding of the historical perspective that led to Puerto Rican identity, their health issues and disparities, and health care access problems of Puerto Ricans on the island and on the mainland. Specific recommendations on how to improve the health care of Puerto Rican patients were provided. The target audience were all health care, public health, and health policy professionals and trainees, and prehealth students. The ideal facilitator is a fellow or faculty member experienced in diversity and inclusion education and/or with knowledge of social determinants of health and/or specific knowledge of Puerto Rican health, history, or health care systems. The workshop employed two instructional strategies: an interactive PowerPoint presentation and a large-group facilitated case discussion and reflection session.

The PowerPoint presentation (Appendix A) began with a brief overview of Puerto Rican history and cultural origins, then delved into health statistics for Puerto Rican populations on the island and on the mainland, including comparisons between these two groups and between Puerto Ricans and other Hispanic and non-Hispanic populations. It also included specific health data and statistics of Puerto Ricans living in New York City as a contextualized example. The workshop continued with three case presentations exercises. During the cases, participants were expected to tie in the information learned during the presentation into the care of three fictional patients through a guided discussion. The facilitator guided participants to discuss and reflect on the health problems of the patient, the risk factors contributing to health problems, problems related with access to health care, and different ways to improve the health care the patient was receiving. The workshop ended with a discussion of recommendations to serve the Puerto Rican community. Participants were asked to conduct pre- and postworkshop self-assessments based upon the presentation's learning objectives and content. Data from IRB-approved sites was analyzed using descriptive statistics for demographic data, paired student *t* tests to compare pre/posttest question results, and thematic analysis based on the workshop's objectives was used to analyze the answers to open-ended questions.

### Appendix A: Taking Care of the Puerto Rican Patient

The content of the workshop is contained within a 50-slide PowerPoint presentation, including a brief history of Puerto Rican identity formation; health status and statistics of Puerto Ricans on the island; influence of migration from the island to the mainland; health status, statistics, and health care access issues of Puerto Ricans on the mainland as compared to other Hispanic and non-Hispanic populations; case presentation and guided discussion; and recommendations for improving the health of the Puerto Rican population.

### Appendix B: Facilitator Guide

The guide outlines step-by-step instructions for the facilitator with specific instructions for conducting the workshop and details discussion points for each slide. Instructions specify how to present each slide to ensure consistent delivery across sites. Facilitators who are not well-versed in Puerto Rican history, culture, and health care may follow the guide and execute the presentation to a high degree.

### Appendix C: Evaluation Forms

The pre- and a postworkshop questionnaire assess participants' confidence aligned to the workshop objectives and overall knowledge of the topics included in the workshop. Additionally, the preworkshop evaluation includes various demographics questions and the postworkshop evaluation includes qualitative feedback questions.

Quantitative data was analyzed using the Mann-Whitney U Test (nonparametric test) to compare pre- and postworkshop results. Qualitative feedback was analyzed through thematic analysis.

A review of the PowerPoint presentation, cases, and evaluation forms by facilitators took approximately 3 hours, but varied with the facilitators' familiarity with the subject matter.

Materials required included audiovisual equipment, a computer for the PowerPoint presentation, and printed copies of the pre- and postworkshop evaluation forms. A summary of the suggested timeline for the workshop was as follows:

1. Preworkshop evaluation: 2–3 minutes
2. Slides 1–7, including introduction, objectives, agenda, history, identity formation: 5 minutes
3. Slides 8–29, including migration, health care status and health statistics of Puerto Ricans on the island and mainland US, and Puerto Ricans in NYC examples: 20 minutes
4. Slides 30–31, with case discussion and reflection exercises: 20 minutes
5. Slide 45–50, on health care recommendations, questions, and answers: 10 minutes
6. Postworkshop evaluation: 2–3 minutes

## Results

There were 68 participants attending the workshop at sites with IRB approval, of which 64 completed the pre- and postworkshop questionnaires. The sites included New York City Health + Hospitals/Metropolitan (an educational clinical affiliate of New York Medical College), Albert Einstein College of Medicine, CUNY School of Medicine, NYU School of Medicine, and Donald and Barbara Zucker School of Medicine at Hofstra/Northwell. Data from these 64 participants were included in the statistical analysis. Participants completed pre- and postworkshop questionnaires.

Participants' roles were diverse with 43% (27) reporting they were medical students, 3% (2) residents or fellows, 19% (12) academic faculty, 8% (5) nonacademic clinicians, and 27% (17) reporting other roles such as university administrators, nurses, genetic counselors, and their trainees. From those who reported their race/ethnicity, 16 (25%) identified as Latino, 11 (17%) as Puerto Rican, 20 (31%) as white, 12 (19%) as Black, 9 (14%) as Asian, 3 (5%) as Native American, and 3 (5%) identified as other. Of note, participants could identify with more than one racial or ethnic identity.

Self-assessment of participants regarding their confidence in their ability to meet the workshop's learning objectives ranged from *no confidence* to *complete confidence*. Results of the Mann-Whitney U Test comparing pre- and postworkshop results demonstrated that, for each objective, confidence improved, and the difference was statistically significant (<.01; Table 1). Results of the Mann-Whitney U Test comparing pre- and postworkshop results for each knowledge question demonstrated a statistically significant improvement (<.01) increase in the average score between pre- and postworkshop test (Table 2).

**Table 1.**
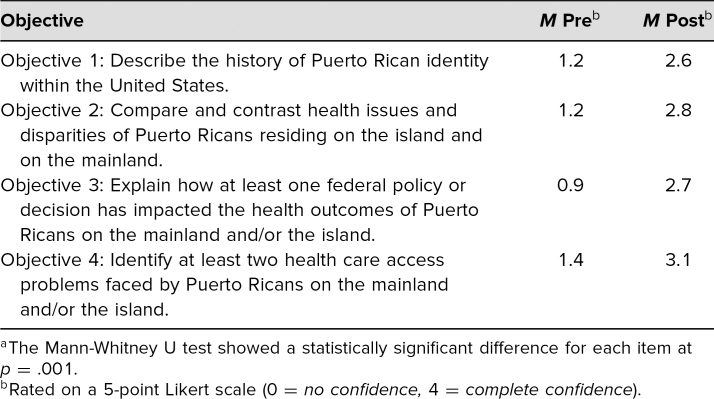
Mean Responses to Pre- and Postworkshop Confidence Questions (*n* = 64)^a^

**Table 2.**
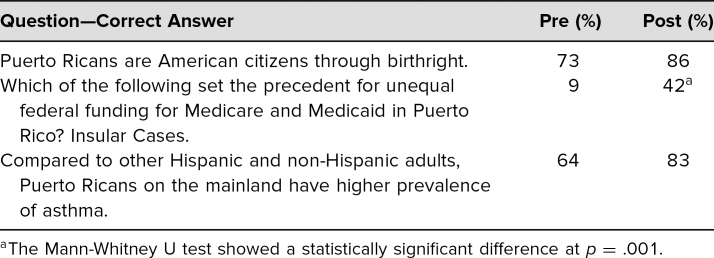
Mean Percent Correct for Pre-/Postworkshop Knowledge Questions (*n* = 64)

Thematic analysis of the qualitative questions resulted in recurring themes. Examples of comments by attendees, organized by these themes according to objectives, included:

• Objective 1: Describe the history of Puerto Rican identity within the United States.
  ○ “I appreciate learning about how the historical context of Puerto Rico and current events affect health care.”
• Objective 2: Compare and contrast health issues and disparities of Puerto Ricans residing on the island and on the mainland.
  ○ “[I] learned differences of health status of Puerto Ricans living in the US on the island; very clear presentation. Thank you for bringing this workshop to us!”
• Objective 3: Explain how at least one state or federal policy has impacted the health outcomes of Puerto Ricans on the mainland and/or the island.
  ○ “Historical perspective helped me understand the different policies that impacted what health care is like today. I had never heard about [the] insular cases.”
• Objective 4: Identify at least two health care access problems faced by Puerto Ricans on the mainland and/or the island.
  ○ Access to care issues were covered as part of the guided case discussions and most of the discussion time was spent on discussing access to care issues. Recurring comments regarding case discussion included:
    ▪ “The cases were a good interactive way to discuss health problems unique to Puerto Ricans.”
    ▪ “The cases were very helpful in putting things into perspective.”

Attendees also provided valuable insight into how to improve the presentation. One recurring theme was to have information about available resources to improve the care of Puerto Rican patients included within the PowerPoint Presentation:

• “More substantial recommendations that we can do to help patients in practice.”
• “More focus on approaches to various challenges related to poor access to health care.”

The most common remarks were about appreciation of the historical context and interactive cases used during the workshop, and about the participants' lack of knowledge about the content discussed prior to participating in the workshop. Less common, but also pertinent, was that several participants would have liked to have a longer workshop and expand on the historical and policy analysis.

## Discussion

This workshop was developed to teach health professionals and trainees unique health issues, disparities, and contributing factors for the Puerto Rican community. It helps remedy the lack of teaching material specific to the secondlargest subgroup of Hispanics and the only one to exist entirely within United States territories. Despite existing entirely within US territories, Puerto Rican communities have evolved into two distinct groups with differing sources of health issues. Hence, the objectives were for participants to understand the history of Puerto Rican identity within the United States, compare and contrast the health issues and disparities of Puerto Ricans residing on the island and on the mainland, explain how federal policies have impacted the health outcomes of Puerto Ricans, and describe health care access problems faced by Puerto Ricans. Overall, evaluations were positive and, according to both quantitative and qualitative data, all objectives were successfully achieved increasing the confidence of the participants. We are certain that the workshop has met its goal of increasing the awareness and understanding of the biopsychosocial factors that impact the health of Puerto Ricans. Thus, we submit that trainees and professionals have demonstrated the educational value of the workshop.

Participants' feedback demonstrated that the workshop structure and content were well received and effective. Participants overwhelmingly commended the historical perspective provided as a way of understanding Puerto Rican culture. Most of them also highlighted the interactive case discussion and reflection as an excellent manner to exemplify health care disparities and access problems faced by Puerto Ricans. Many also positively commented on the cofacilitators speaking skills and knowledge of the material.

Opportunities for improvement were also derived from participants' feedback and from the postworkshop questionnaire results. Many attendees wanted more resources and specific recommendations to improve the health care of their Puerto Rican patients, including research opportunities. Some attendees commented that they would have liked more time to discuss each case in small groups prior to the large group exercise. Although there was a statistically significant increase in knowledge to the question regarding policies (precedent for unequal federal funding for Medicare and Medicaid in Puerto Rico), the postworkshop percent correct was only 42%, thus a clearer explanation of the insular cases is needed.

The workshop's content was revised and updated on subsequent presentations based on the results and feedback from earlier sessions. A clearer description of insular cases is now contained in the facilitator's guide (Appendix B). We improved the recommendation slides including more specific information and research recommendations and added two slides containing specific resources for health care providers. The facilitator's guide also contains an alternative to the 60-minute workshop, which would be to divide the large group into small groups with at least three facilitators to discuss the cases. If this alternative is used, then the workshop's allotted time should increase to at least 90 minutes.

Despite meeting the objectives, there were limitations and challenges to the implementation and evaluation of the workshop. This workshop was conducted by two of the coauthors who identify as Puerto Ricans and self-trained for the development and implementation of the workshop. To account for this limitation, we provided an expansive facilitator guide to well prepare facilitators. As with any topic, faculty should review the workshop's material and reflect on whether they have the aptitude to learn and teach the content. Once the workshop is implemented, faculty should also improve their teaching based on the results of the postworkshop evaluation.

Although specifically tailored to increase the knowledge and confidence in health care providers taking care of Puerto Rican patients, this workshop can be used as part of any medical school or residency program's health disparities/communication skills curriculum. The former would assist medical schools in meeting the LCME requirements by enhancing knowledge and awareness of diversity and health disparities, and by improving culturally competent care. The latter would assist residency programs in meeting the ACGME requirements of teaching and assessing residents' competency in providing competent care for a broad range of patients through adequate communication skills and program requirements in providing a diverse learning environment. Since residents provide direct patient care, residency programs could not only assess confidence and knowledge after the workshop, but also measure the impact of this increase in knowledge and confidence by assessing the Puerto Rican patients' perception of how they are being cared for before and after the implementation of the workshop. This workshop can also be used as a stepping stone towards advocacy and tailoring curricula to address the growing needs of the increasing conglomerate that is the Hispanic community. Future researchers can use this workshop as an example to create modules for other unique communities with large ethnic enclaves within the United States (e.g., Mexican/Mexican American/Chicano, Cuban, Dominican, Colombian, etc.). The emergence of these modules will help to accurately identify these populations as distinct groups—as opposed to grouping them under the monolithic Hispanic or Latino categories. Furthermore, for the Puerto Rican community much of the information contained within this workshop can be utilized by advocacy groups (e.g., AMA, National Hispanic Medical Association, etc.) to lobby for policy changes that will improve the health care of Puerto Ricans on the island and on mainland United States.

## Appendices

Taking Care of the Puerto Rican Patient.pptxFacilitator Guide.docxEvaluation Forms.docx
All appendices are peer reviewed as integral parts of the Original Publication.
