# Supplementary material for: Taking Care of the Puerto Rican Patient: Historical Perspectives, Health Status, and Health Care Access
Source: MedEdPORTAL. 2020 Oct 7;16:10984. doi: 10.15766/mep_2374-8265.10984 (PMC7549386; doi:10.15766/mep_2374-8265.10984)
Supplement: Supplementary file 1 — Taking Care of the Puerto Rican Patient.pptxFacilitator Guide.docxEvaluation Forms.docx [file mep_2374-8265.10984-s001.zip › A. Taking Care of the Puerto Rican Patient.pptx]

## Slide 1
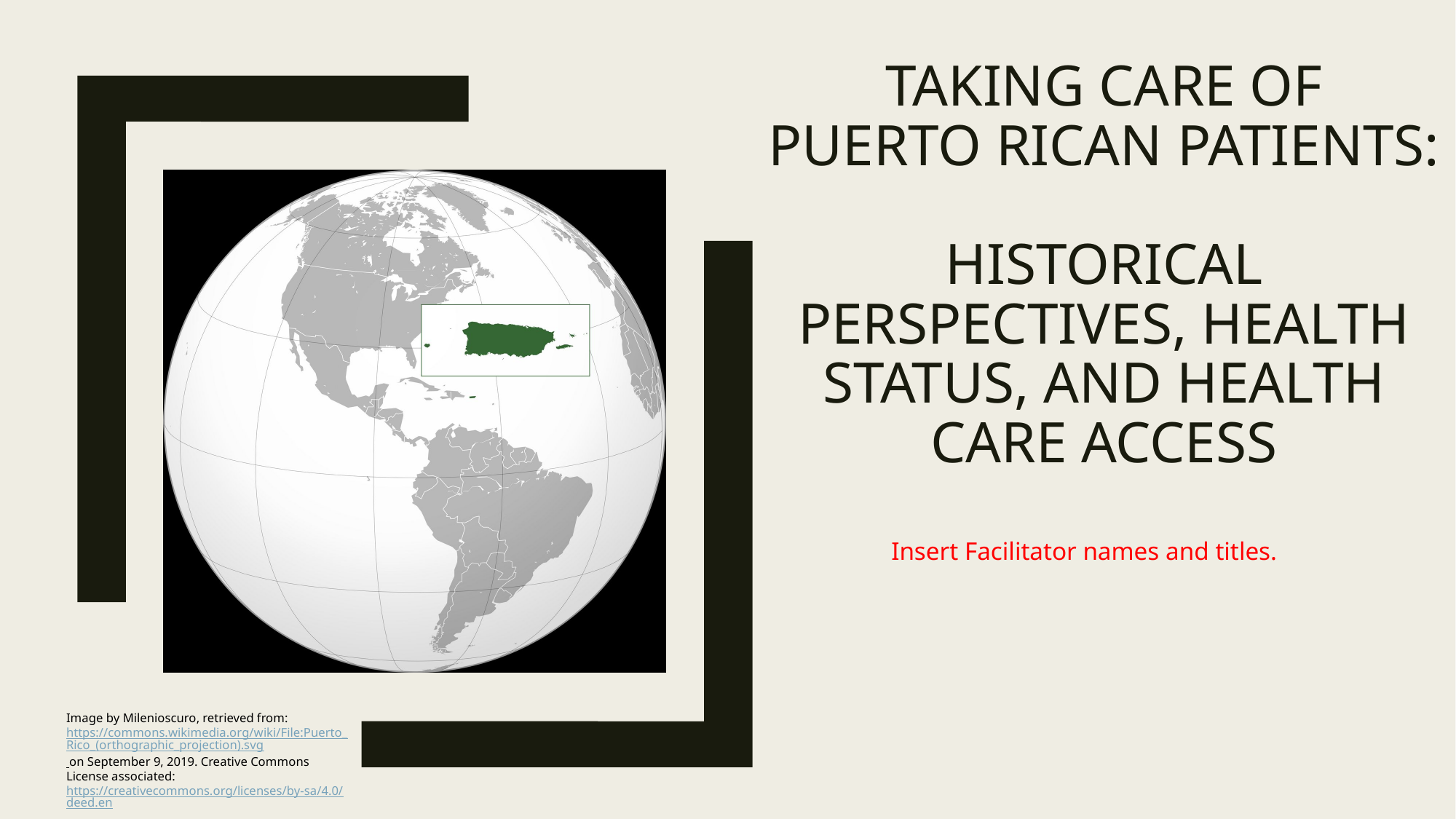

# Taking Care of Puerto Rican Patients: Historical Perspectives, Health Status, and Health Care Access
Insert Facilitator names and titles.
Image by Milenioscuro, retrieved from: https://commons.wikimedia.org/wiki/File:Puerto_Rico_(orthographic_projection).svg on September 9, 2019. Creative Commons License associated: https://creativecommons.org/licenses/by-sa/4.0/deed.en.

## Slide 2
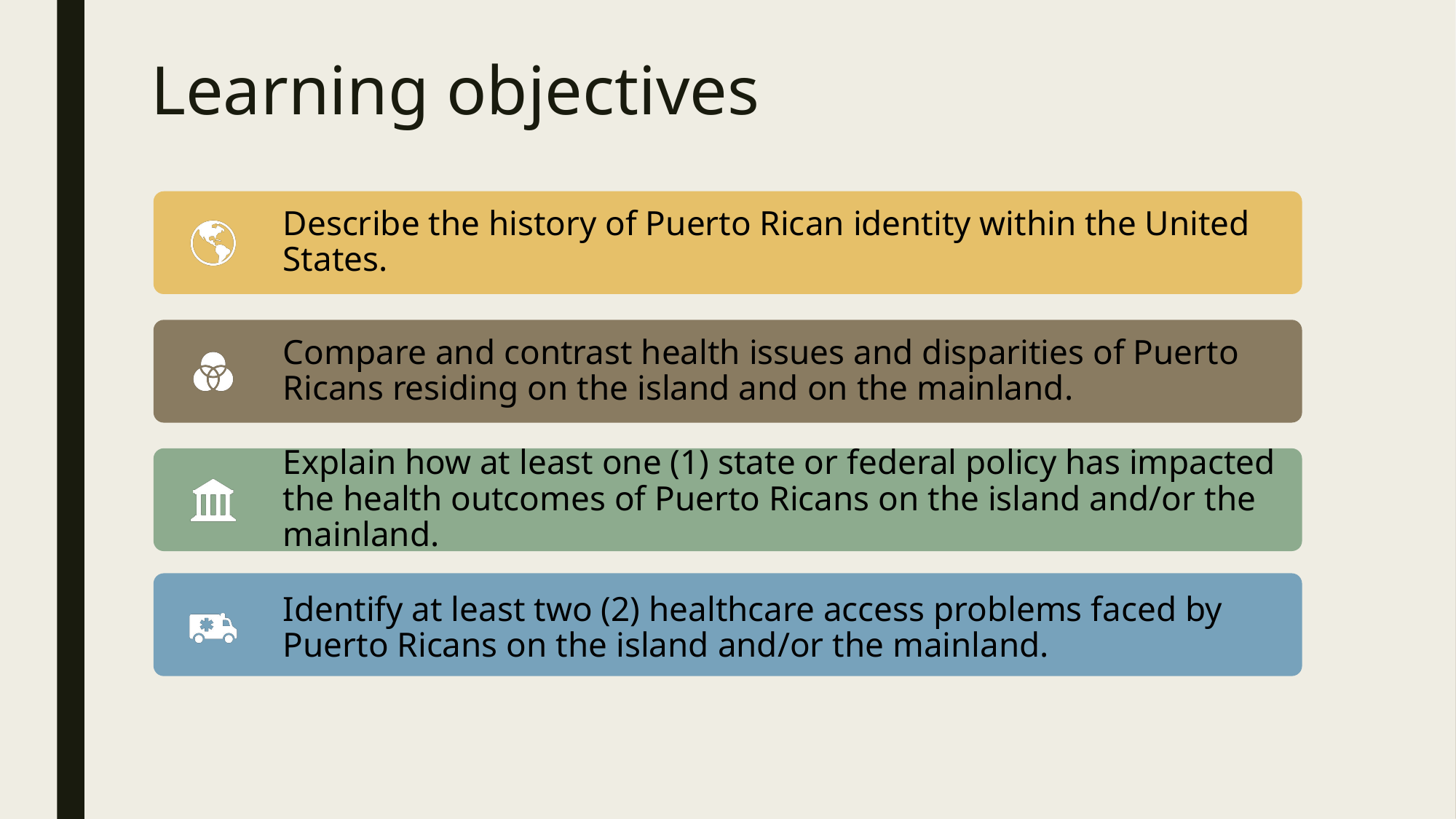

# Learning objectives

## Slide 3
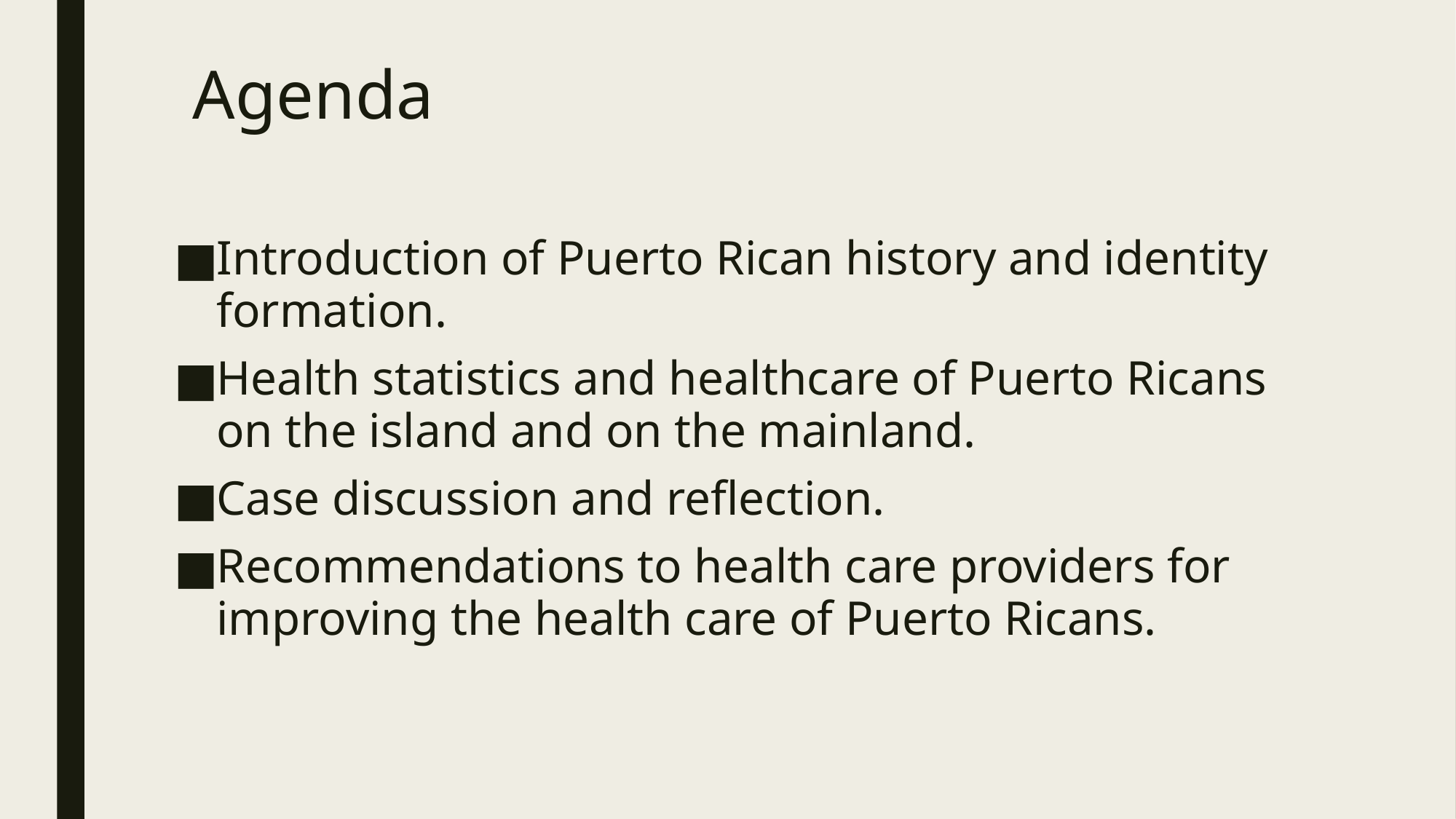

# Agenda
Introduction of Puerto Rican history and identity formation.
Health statistics and healthcare of Puerto Ricans on the island and on the mainland.
Case discussion and reflection.
Recommendations to health care providers for improving the health care of Puerto Ricans.

## Slide 4
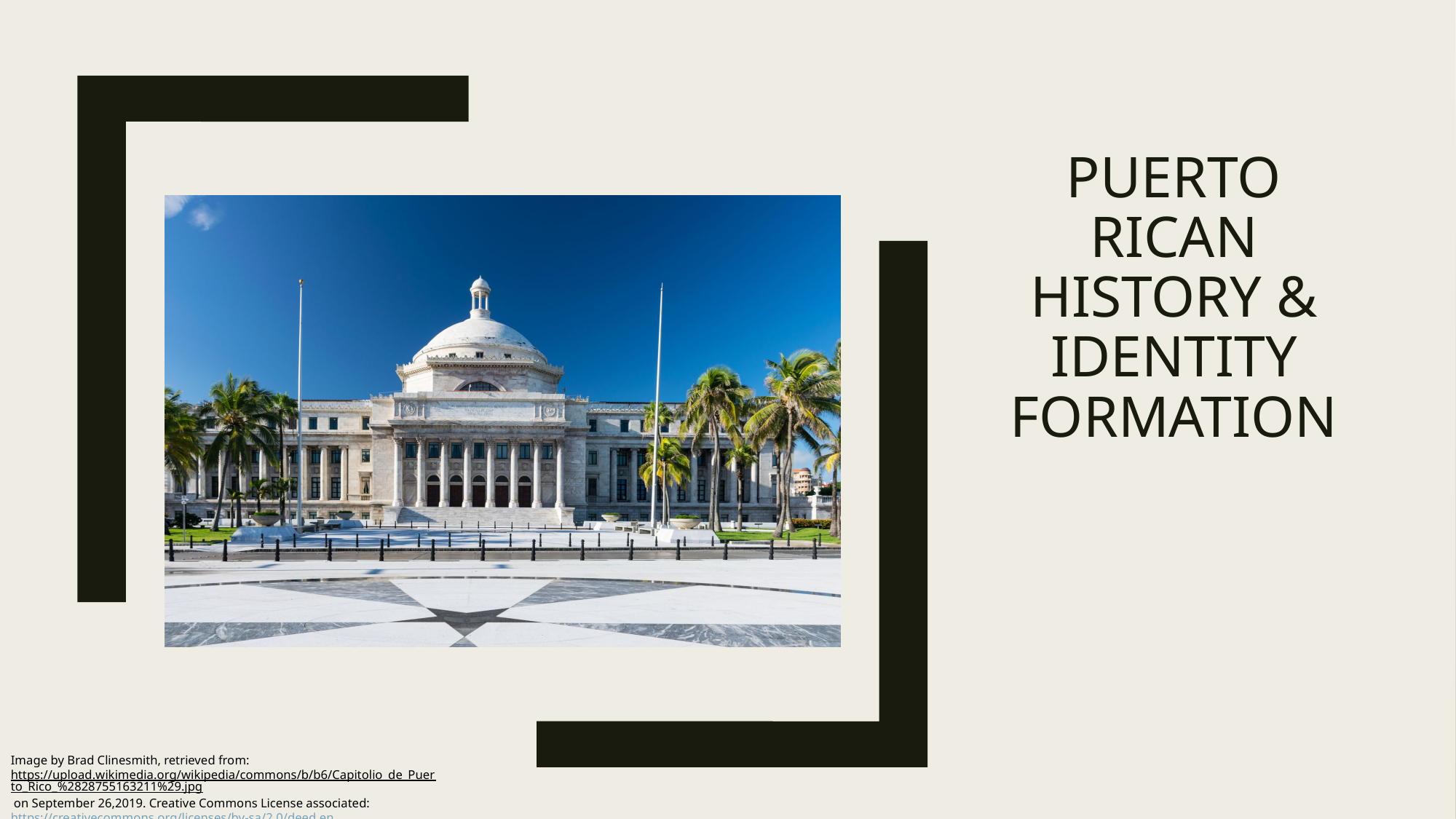

# Puerto Rican History & Identity Formation
Image by Brad Clinesmith, retrieved from: https://upload.wikimedia.org/wikipedia/commons/b/b6/Capitolio_de_Puerto_Rico_%2828755163211%29.jpg on September 26,2019. Creative Commons License associated: https://creativecommons.org/licenses/by-sa/2.0/deed.en.

## Slide 5
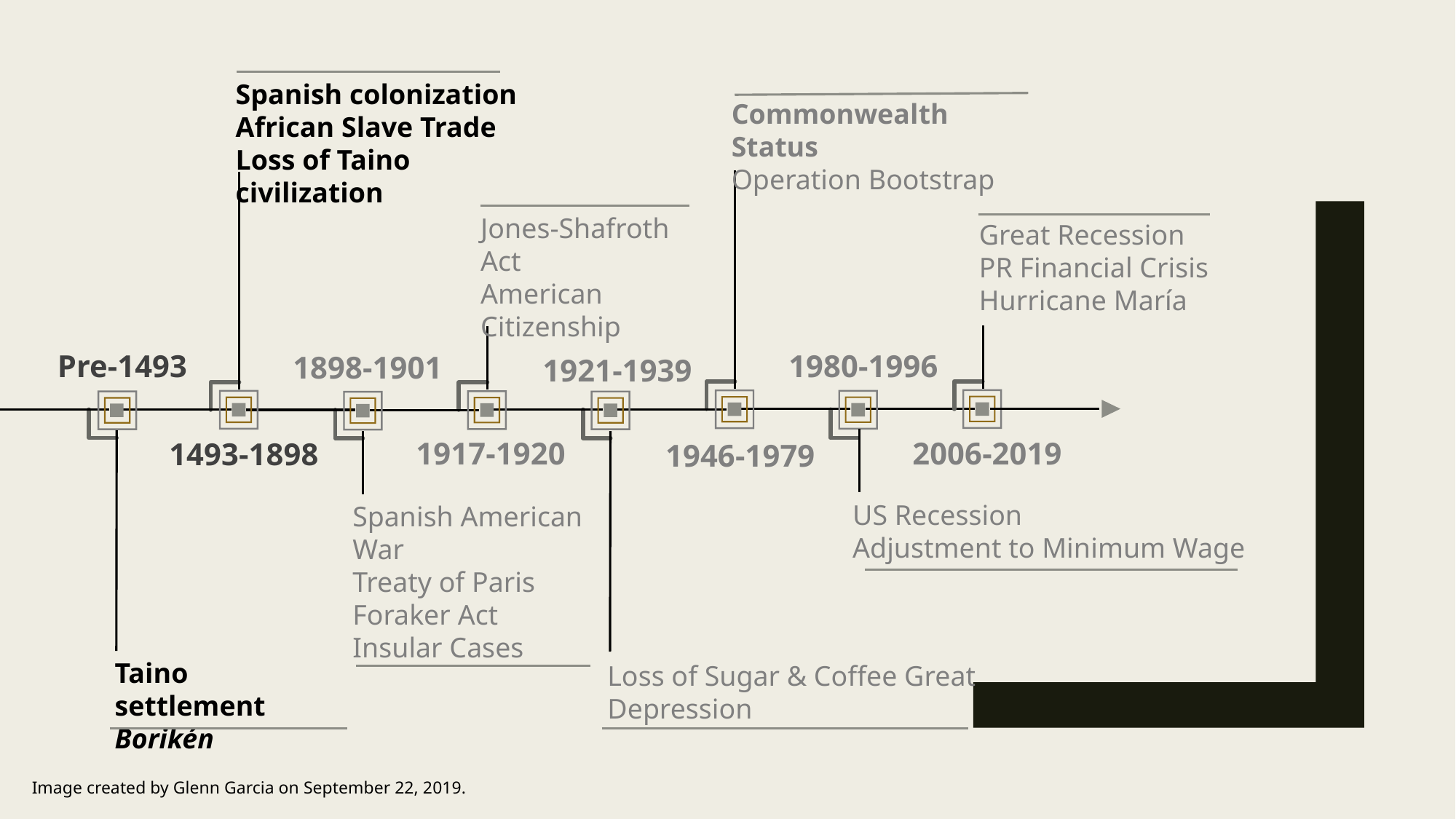

Spanish colonization
African Slave Trade
Loss of Taino civilization
Commonwealth Status
Operation Bootstrap
Jones-Shafroth Act
American Citizenship
Great Recession
PR Financial Crisis
Hurricane María
Pre-1493
1980-1996
1898-1901
1921-1939
1917-1920
2006-2019
1493-1898
1946-1979
US Recession
Adjustment to Minimum Wage
Spanish American
War
Treaty of Paris
Foraker Act
Insular Cases
Taino settlement Borikén
Loss of Sugar & Coffee Great Depression
Image created by Glenn Garcia on September 22, 2019.

## Slide 6
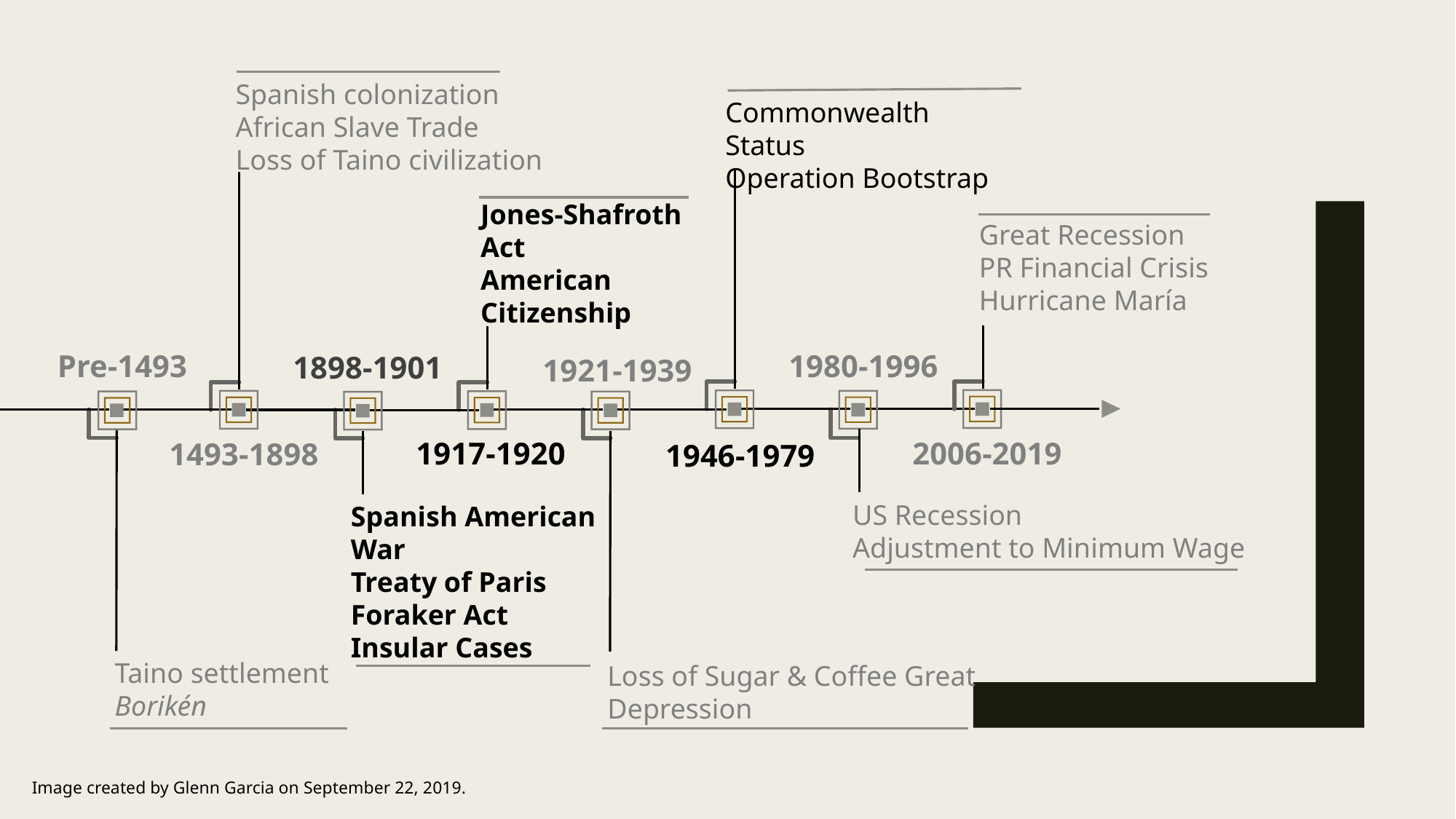

Spanish colonization
African Slave Trade
Loss of Taino civilization
Commonwealth Status
Operation Bootstrap
Jones-Shafroth Act
American Citizenship
Great Recession
PR Financial Crisis
Hurricane María
Pre-1493
1980-1996
1898-1901
1921-1939
1917-1920
2006-2019
1493-1898
1946-1979
US Recession
Adjustment to Minimum Wage
Spanish American
War
Treaty of Paris
Foraker Act
Insular Cases
Taino settlement Borikén
Loss of Sugar & Coffee Great Depression
Image created by Glenn Garcia on September 22, 2019.

## Slide 7
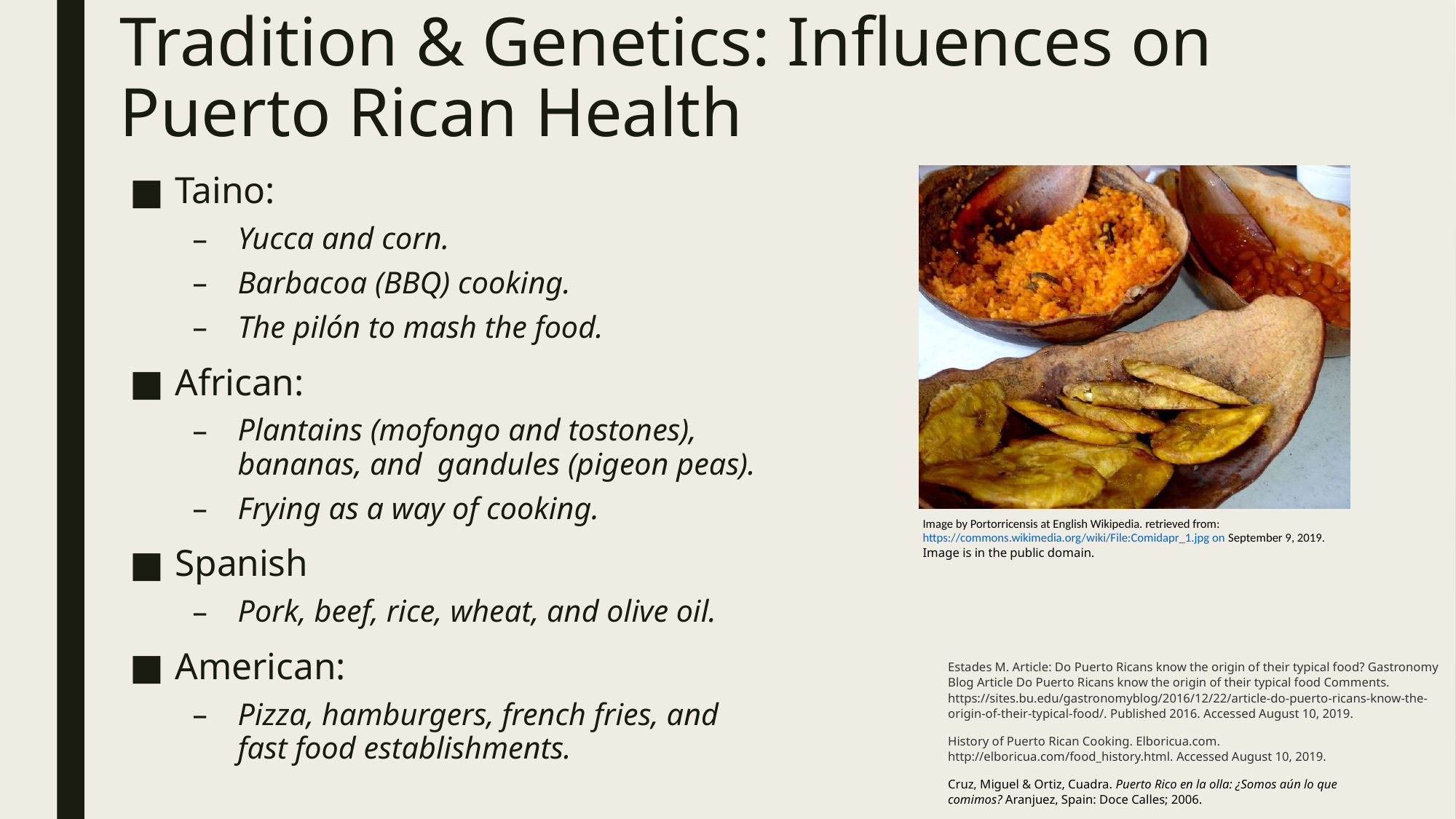

Tradition & Genetics: Influences on Puerto Rican Health
Taino:
Yucca and corn.
Barbacoa (BBQ) cooking.
The pilón to mash the food.
African:
Plantains (mofongo and tostones), bananas, and gandules (pigeon peas).
Frying as a way of cooking.
Spanish
Pork, beef, rice, wheat, and olive oil.
American:
Pizza, hamburgers, french fries, and fast food establishments.
Image by Portorricensis at English Wikipedia. retrieved from: https://commons.wikimedia.org/wiki/File:Comidapr_1.jpg on September 9, 2019. Image is in the public domain.
Estades M. Article: Do Puerto Ricans know the origin of their typical food? Gastronomy Blog Article Do Puerto Ricans know the origin of their typical food Comments. https://sites.bu.edu/gastronomyblog/2016/12/22/article-do-puerto-ricans-know-the-origin-of-their-typical-food/. Published 2016. Accessed August 10, 2019.
History of Puerto Rican Cooking. Elboricua.com. http://elboricua.com/food_history.html. Accessed August 10, 2019.
Cruz, Miguel & Ortiz, Cuadra. Puerto Rico en la olla: ¿Somos aún lo que comimos? Aranjuez, Spain: Doce Calles; 2006.

## Slide 8
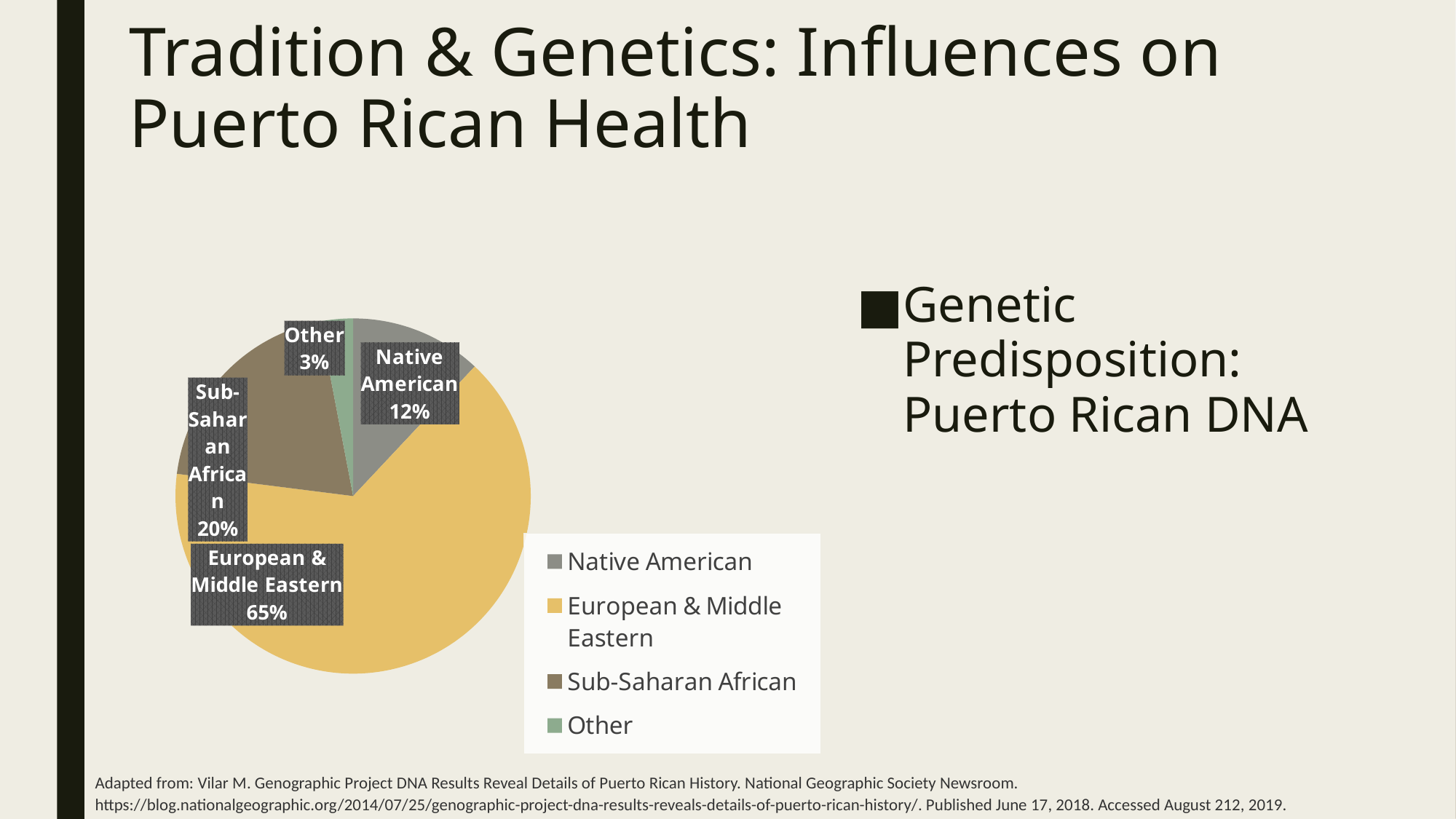

# Tradition & Genetics: Influences on Puerto Rican Health
### Chart
| Category | Column1 |
|---|---|
| Native American | 12.0 |
| European & Middle Eastern | 65.0 |
| Sub-Saharan African | 20.0 |
| Other | 3.0 |Genetic Predisposition: Puerto Rican DNA
Adapted from: Vilar M. Genographic Project DNA Results Reveal Details of Puerto Rican History. National Geographic Society Newsroom. https://blog.nationalgeographic.org/2014/07/25/genographic-project-dna-results-reveals-details-of-puerto-rican-history/. Published June 17, 2018. Accessed August 212, 2019.

## Slide 9
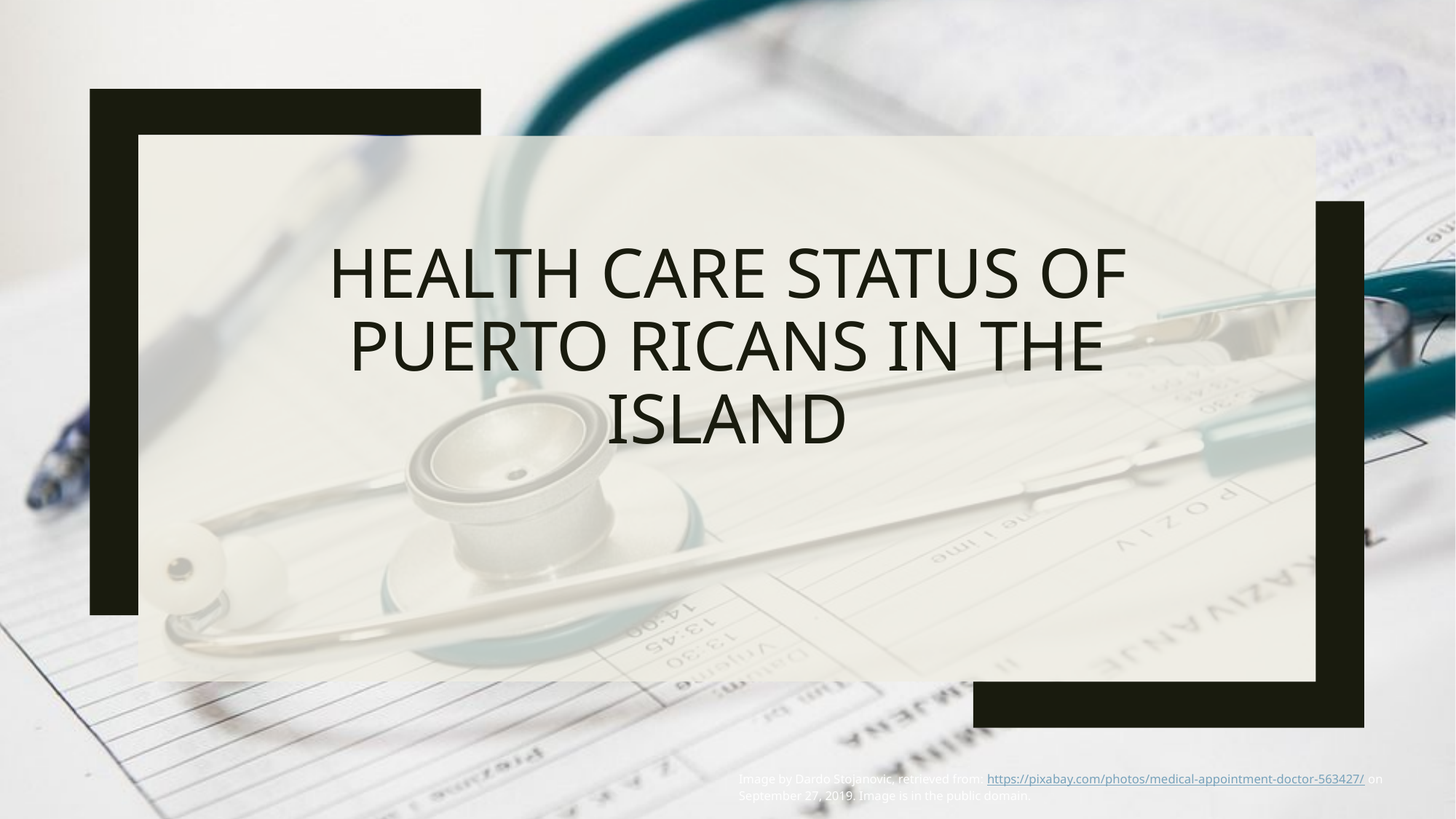

# Health Care Status of Puerto Ricans in the Island
Image by Dardo Stojanovic, retrieved from: https://pixabay.com/photos/medical-appointment-doctor-563427/ on September 27, 2019. Image is in the public domain.

## Slide 10
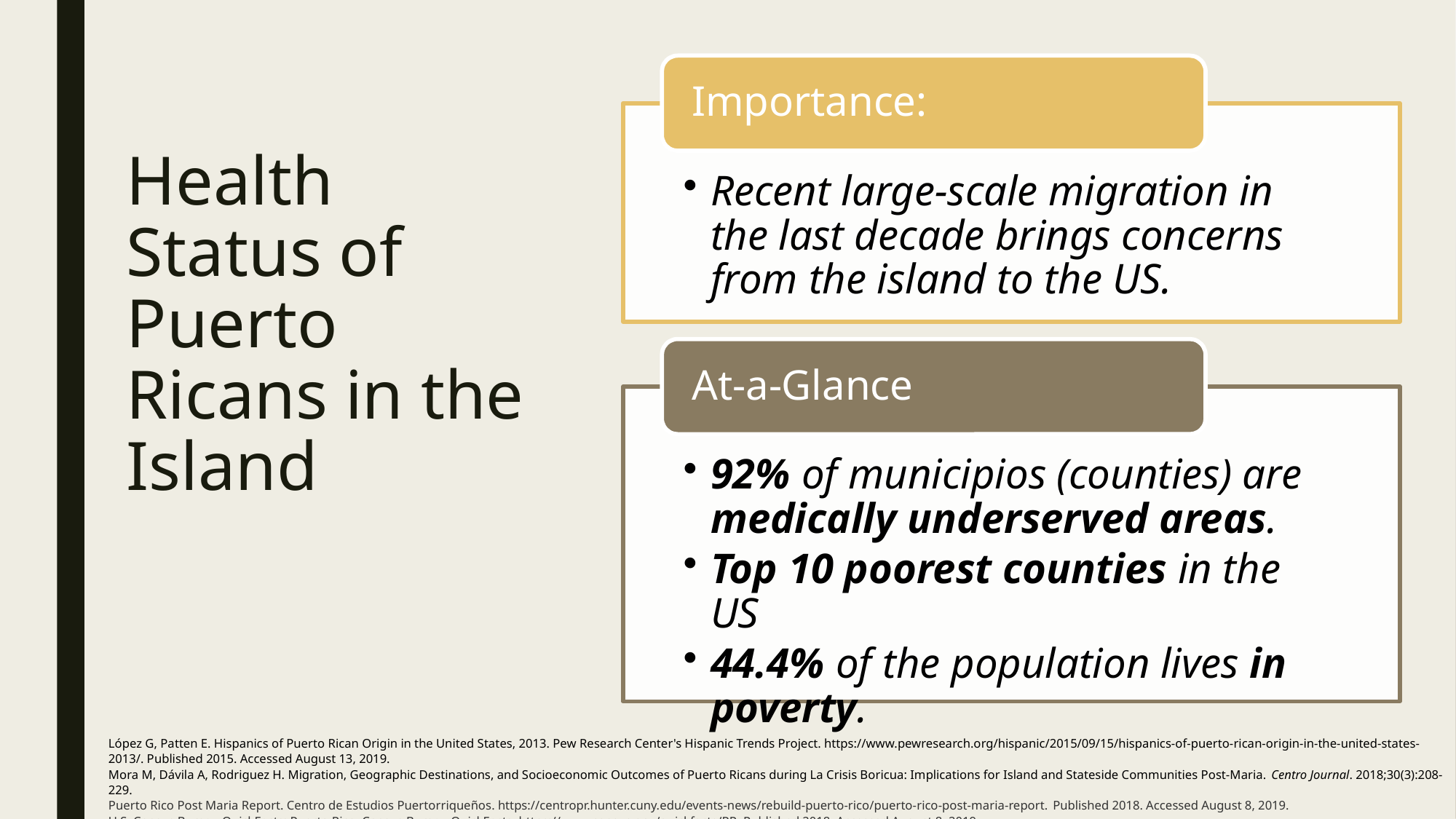

# Health Status of Puerto Ricans in the Island
López G, Patten E. Hispanics of Puerto Rican Origin in the United States, 2013. Pew Research Center's Hispanic Trends Project. https://www.pewresearch.org/hispanic/2015/09/15/hispanics-of-puerto-rican-origin-in-the-united-states-2013/. Published 2015. Accessed August 13, 2019.
Mora M, Dávila A, Rodriguez H. Migration, Geographic Destinations, and Socioeconomic Outcomes of Puerto Ricans during La Crisis Boricua: Implications for Island and Stateside Communities Post-Maria. Centro Journal. 2018;30(3):208-229.
Puerto Rico Post Maria Report. Centro de Estudios Puertorriqueños. https://centropr.hunter.cuny.edu/events-news/rebuild-puerto-rico/puerto-rico-post-maria-report. Published 2018. Accessed August 8, 2019.
U.S. Census Bureau QuickFacts: Puerto Rico. Census Bureau QuickFacts. https://www.census.gov/quickfacts/PR. Published 2018. Accessed August 8, 2019.

## Slide 11
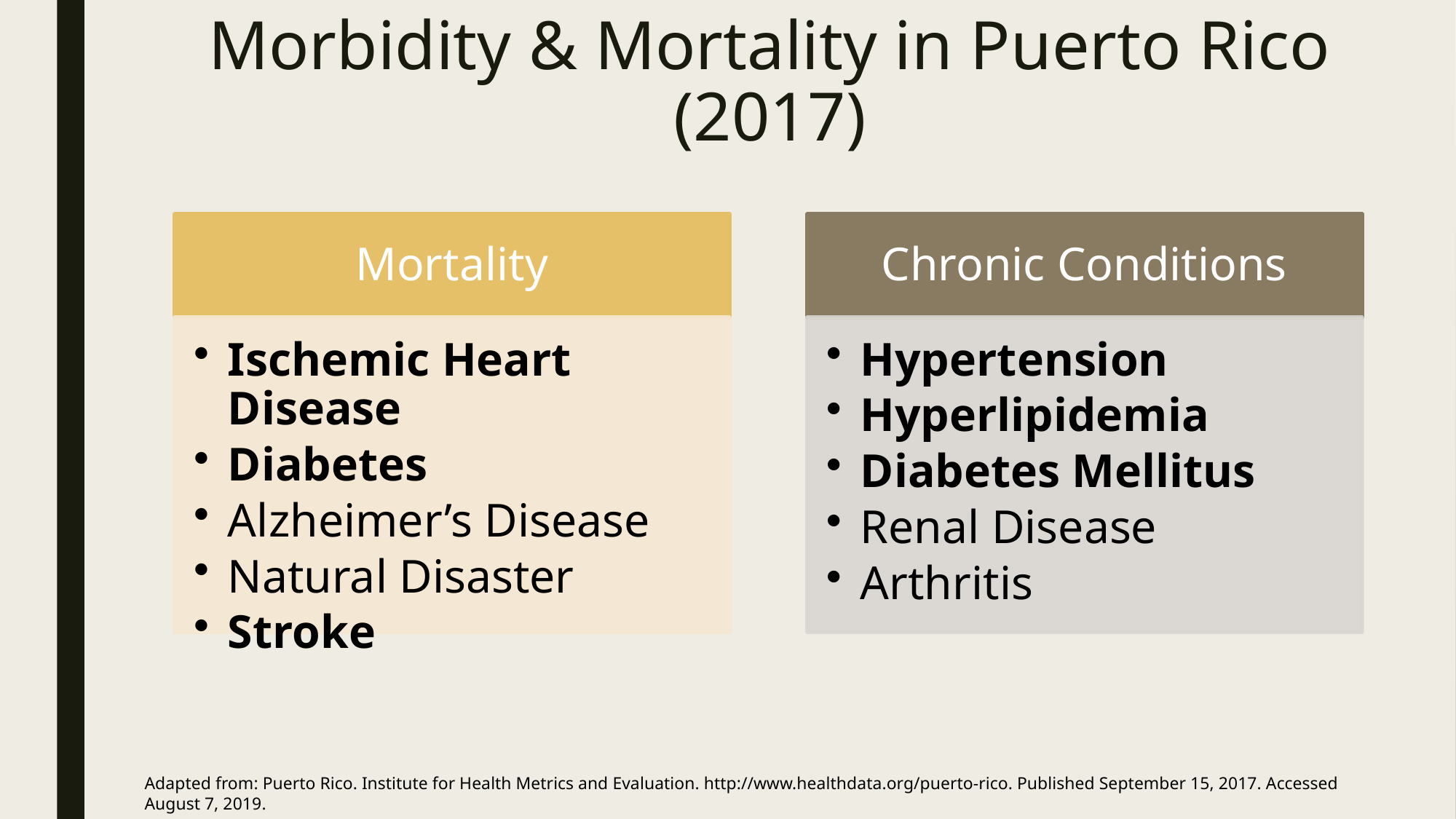

# Morbidity & Mortality in Puerto Rico (2017)
Adapted from: Puerto Rico. Institute for Health Metrics and Evaluation. http://www.healthdata.org/puerto-rico. Published September 15, 2017. Accessed August 7, 2019.

## Slide 12
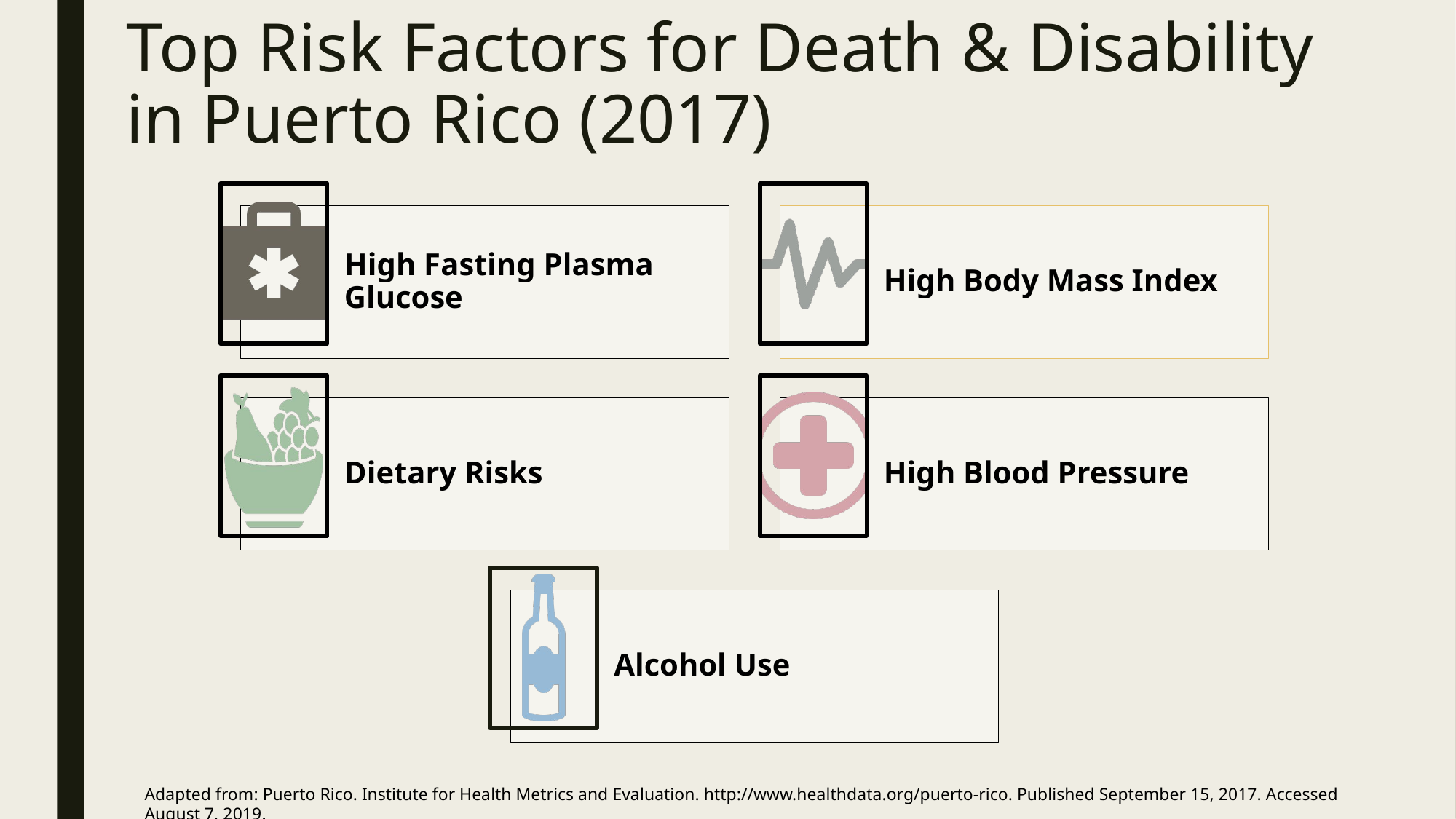

Top Risk Factors for Death & Disability in Puerto Rico (2017)
Adapted from: Puerto Rico. Institute for Health Metrics and Evaluation. http://www.healthdata.org/puerto-rico. Published September 15, 2017. Accessed August 7, 2019.

## Slide 13
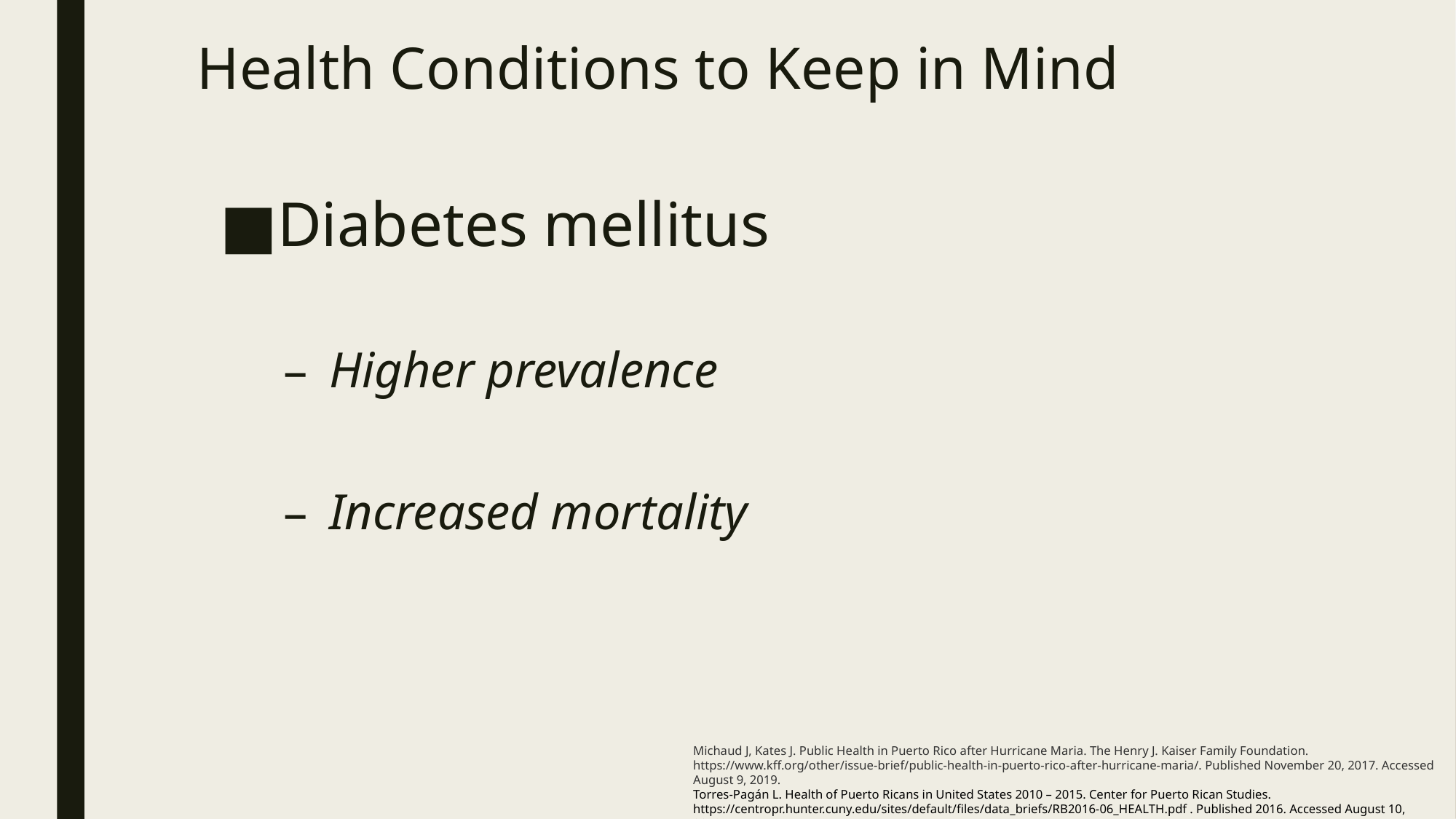

Health Conditions to Keep in Mind
Diabetes mellitus
Higher prevalence
Increased mortality
Michaud J, Kates J. Public Health in Puerto Rico after Hurricane Maria. The Henry J. Kaiser Family Foundation. https://www.kff.org/other/issue-brief/public-health-in-puerto-rico-after-hurricane-maria/. Published November 20, 2017. Accessed August 9, 2019.
Torres-Pagán L. Health of Puerto Ricans in United States 2010 – 2015. Center for Puerto Rican Studies. https://centropr.hunter.cuny.edu/sites/default/files/data_briefs/RB2016-06_HEALTH.pdf . Published 2016. Accessed August 10, 2019.

## Slide 14
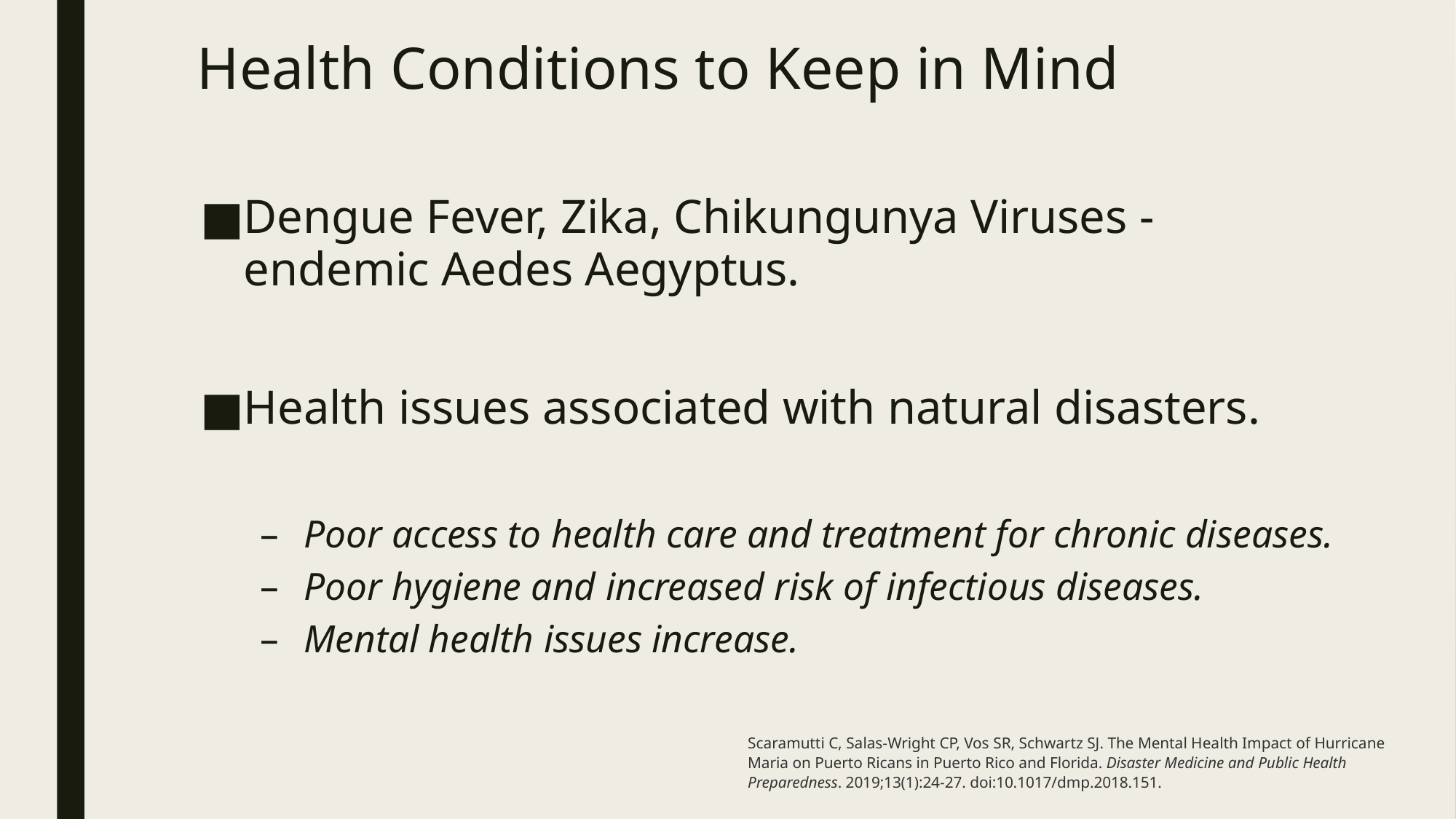

# Health Conditions to Keep in Mind
Dengue Fever, Zika, Chikungunya Viruses - endemic Aedes Aegyptus.
Health issues associated with natural disasters.
Poor access to health care and treatment for chronic diseases.
Poor hygiene and increased risk of infectious diseases.
Mental health issues increase.
Scaramutti C, Salas-Wright CP, Vos SR, Schwartz SJ. The Mental Health Impact of Hurricane Maria on Puerto Ricans in Puerto Rico and Florida. Disaster Medicine and Public Health Preparedness. 2019;13(1):24-27. doi:10.1017/dmp.2018.151.

## Slide 15
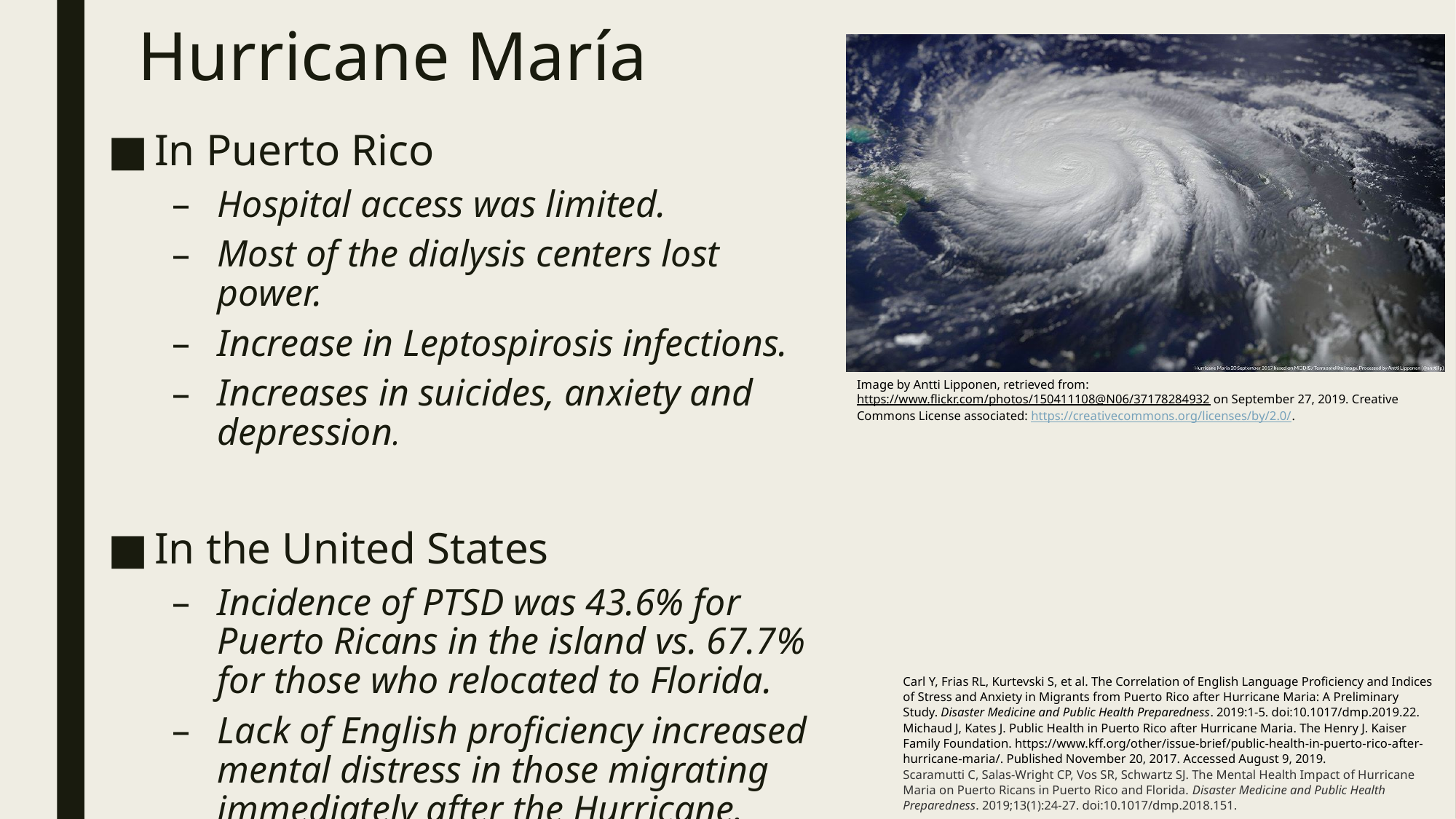

Hurricane María
In Puerto Rico
Hospital access was limited.
Most of the dialysis centers lost power.
Increase in Leptospirosis infections.
Increases in suicides, anxiety and depression.
In the United States
Incidence of PTSD was 43.6% for Puerto Ricans in the island vs. 67.7% for those who relocated to Florida.
Lack of English proficiency increased mental distress in those migrating immediately after the Hurricane.
Image by Antti Lipponen, retrieved from: https://www.flickr.com/photos/150411108@N06/37178284932 on September 27, 2019. Creative Commons License associated: https://creativecommons.org/licenses/by/2.0/.
Carl Y, Frias RL, Kurtevski S, et al. The Correlation of English Language Proficiency and Indices of Stress and Anxiety in Migrants from Puerto Rico after Hurricane Maria: A Preliminary Study. Disaster Medicine and Public Health Preparedness. 2019:1-5. doi:10.1017/dmp.2019.22.
Michaud J, Kates J. Public Health in Puerto Rico after Hurricane Maria. The Henry J. Kaiser Family Foundation. https://www.kff.org/other/issue-brief/public-health-in-puerto-rico-after-hurricane-maria/. Published November 20, 2017. Accessed August 9, 2019.
Scaramutti C, Salas-Wright CP, Vos SR, Schwartz SJ. The Mental Health Impact of Hurricane Maria on Puerto Ricans in Puerto Rico and Florida. Disaster Medicine and Public Health Preparedness. 2019;13(1):24-27. doi:10.1017/dmp.2018.151.

## Slide 16
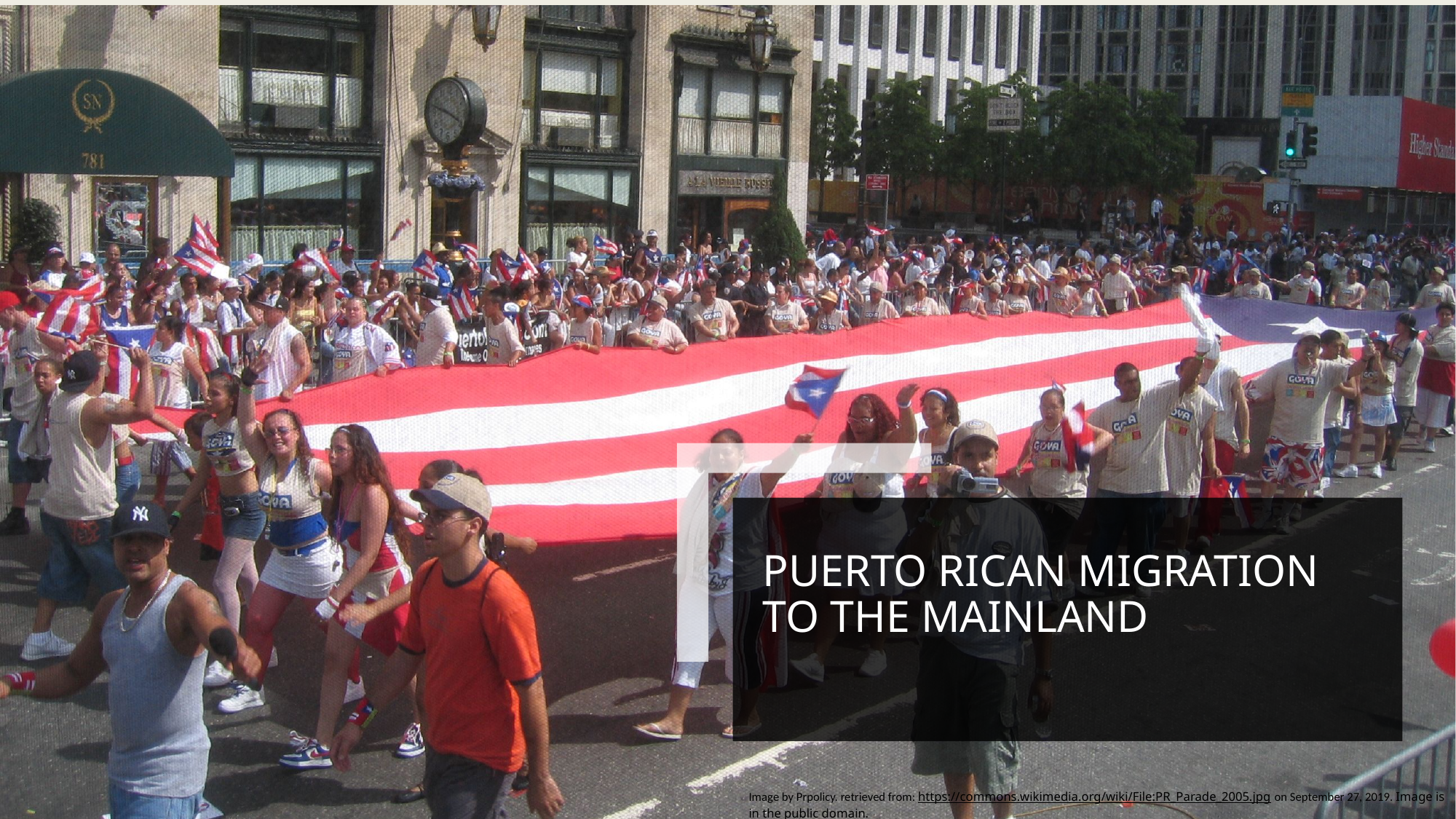

# Puerto Rican Migration to the mainland
Image by Prpolicy. retrieved from: https://commons.wikimedia.org/wiki/File:PR_Parade_2005.jpg on September 27, 2019. Image is in the public domain.

## Slide 17
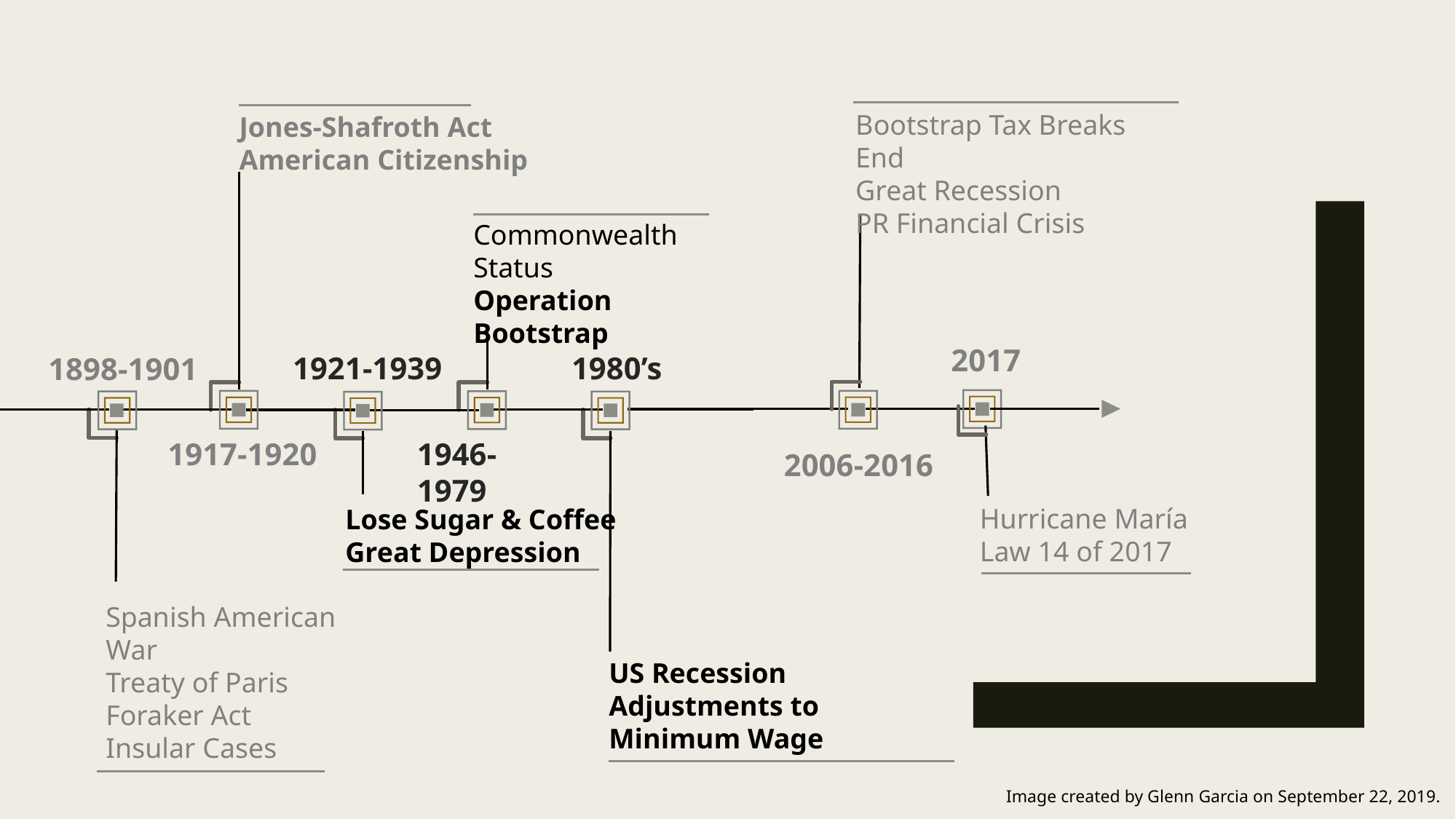

Bootstrap Tax Breaks End
Great Recession
PR Financial Crisis
Jones-Shafroth Act
American Citizenship
Commonwealth Status
Operation Bootstrap
2017
1921-1939
1980’s
1898-1901
1917-1920
1946-1979
2006-2016
Hurricane María
Law 14 of 2017
Lose Sugar & Coffee
Great Depression
Spanish American
War
Treaty of Paris
Foraker Act
Insular Cases
US Recession
Adjustments to Minimum Wage
Image created by Glenn Garcia on September 22, 2019.

## Slide 18
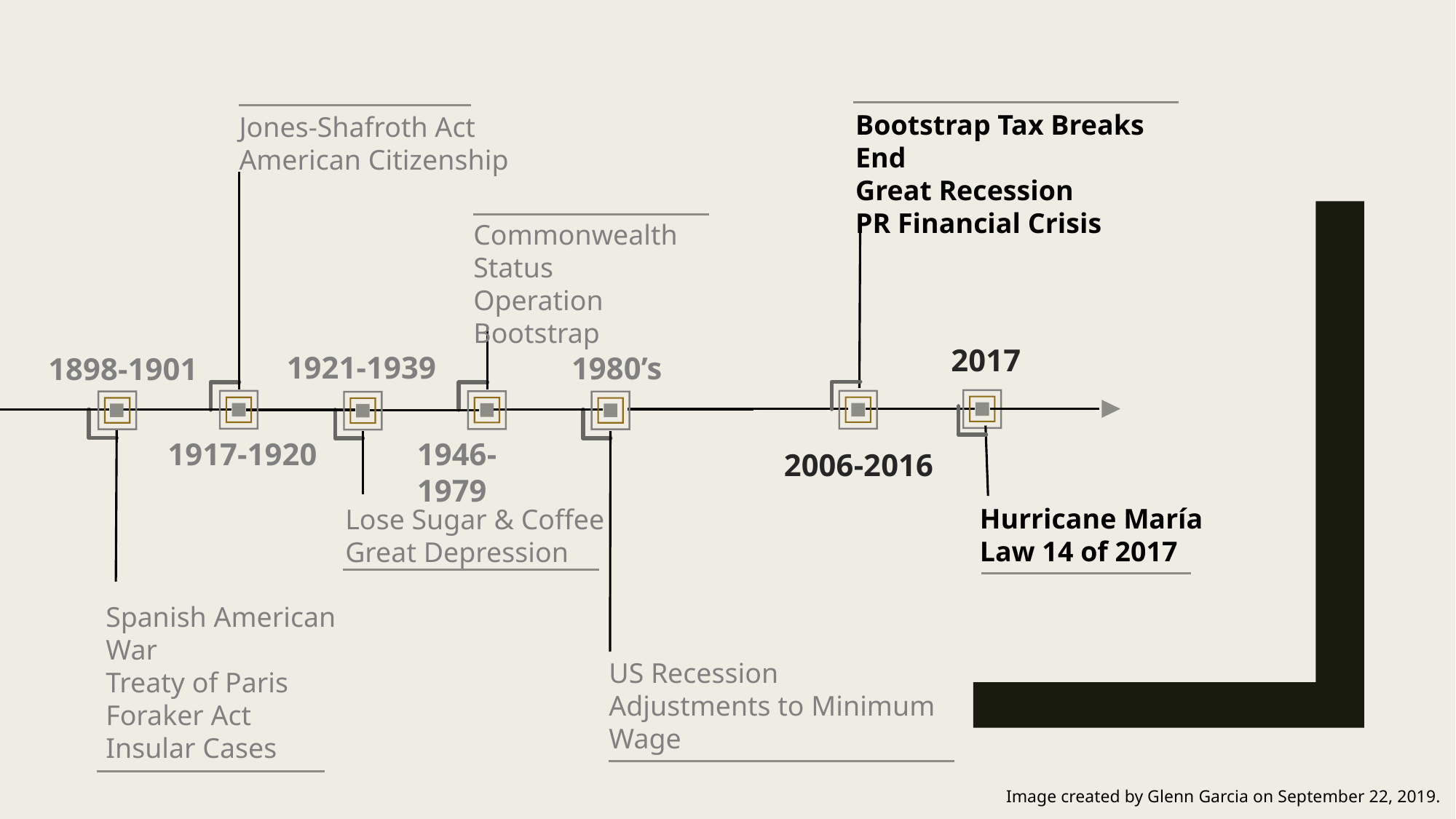

Bootstrap Tax Breaks End
Great Recession
PR Financial Crisis
Jones-Shafroth Act
American Citizenship
Commonwealth Status
Operation Bootstrap
2017
1921-1939
1980’s
1898-1901
1917-1920
1946-1979
2006-2016
Hurricane María
Law 14 of 2017
Lose Sugar & Coffee
Great Depression
Spanish American
War
Treaty of Paris
Foraker Act
Insular Cases
US Recession
Adjustments to Minimum Wage
Image created by Glenn Garcia on September 22, 2019.

## Slide 19
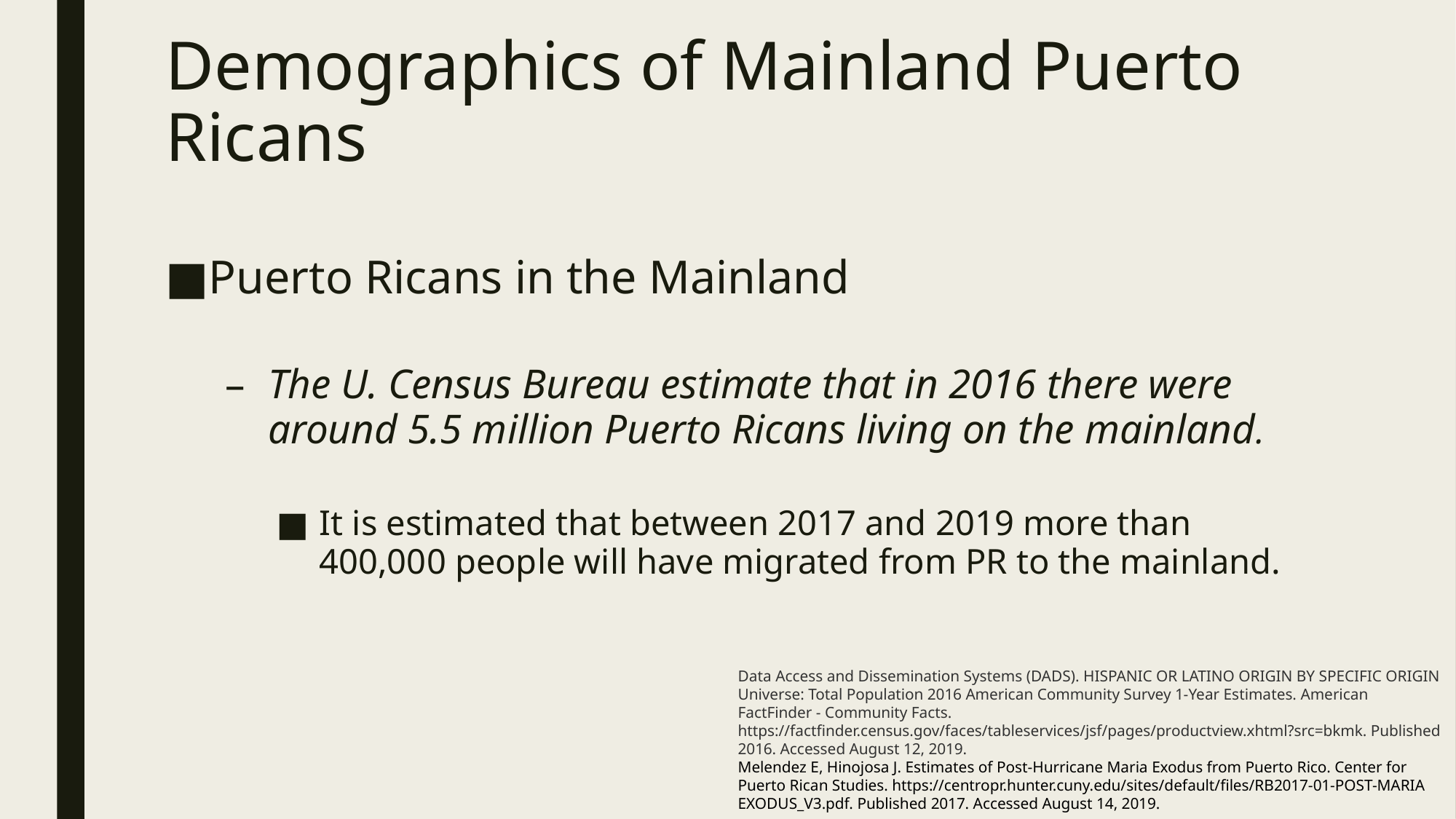

Demographics of Mainland Puerto Ricans
Puerto Ricans in the Mainland
The U. Census Bureau estimate that in 2016 there were around 5.5 million Puerto Ricans living on the mainland.
It is estimated that between 2017 and 2019 more than 400,000 people will have migrated from PR to the mainland.
Data Access and Dissemination Systems (DADS). HISPANIC OR LATINO ORIGIN BY SPECIFIC ORIGIN Universe: Total Population 2016 American Community Survey 1-Year Estimates. American FactFinder - Community Facts. https://factfinder.census.gov/faces/tableservices/jsf/pages/productview.xhtml?src=bkmk. Published 2016. Accessed August 12, 2019.
Melendez E, Hinojosa J. Estimates of Post-Hurricane Maria Exodus from Puerto Rico. Center for Puerto Rican Studies. https://centropr.hunter.cuny.edu/sites/default/files/RB2017-01-POST-MARIA EXODUS_V3.pdf. Published 2017. Accessed August 14, 2019.

## Slide 20
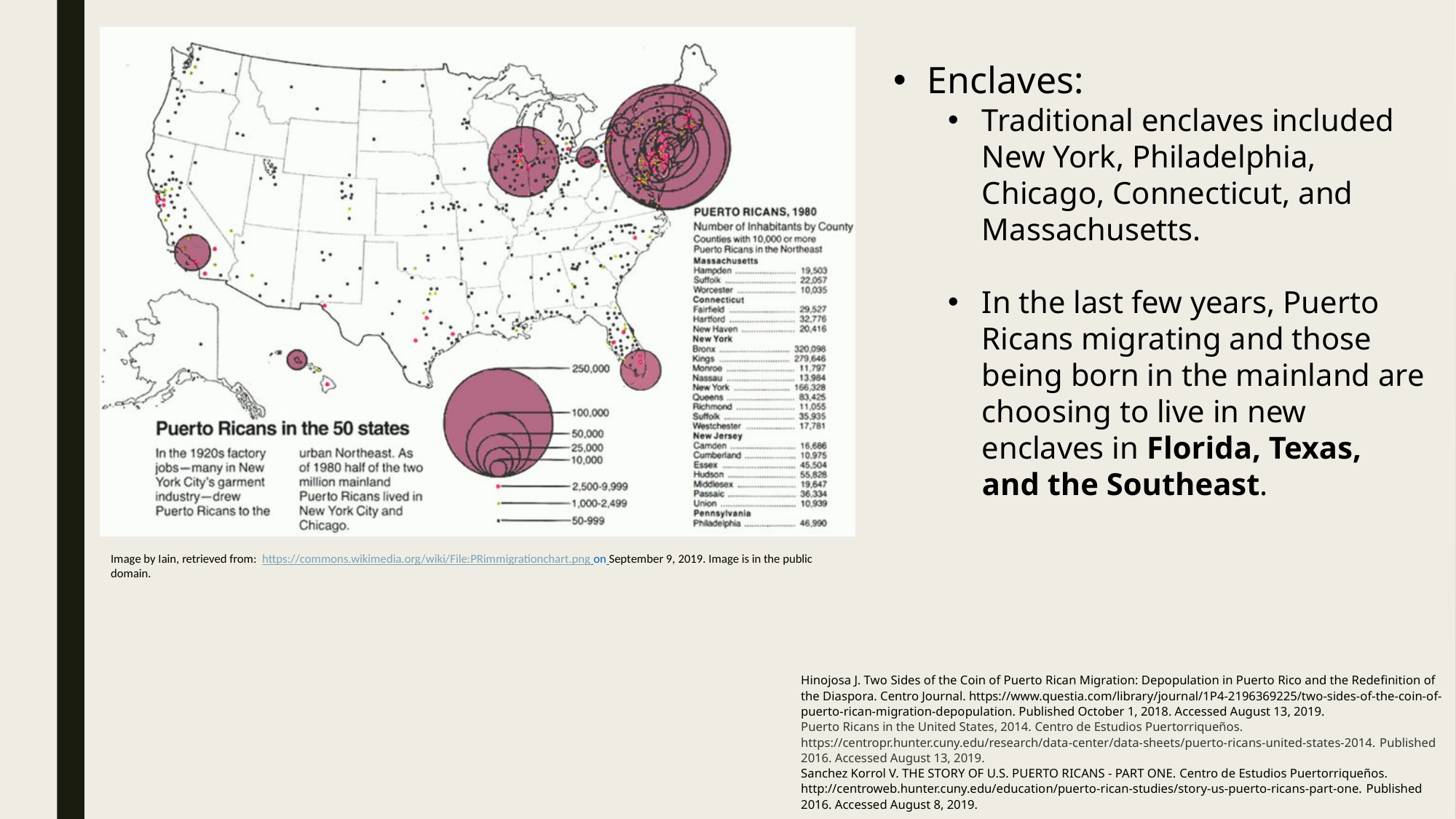

Enclaves:
Traditional enclaves included New York, Philadelphia, Chicago, Connecticut, and Massachusetts.
In the last few years, Puerto Ricans migrating and those being born in the mainland are choosing to live in new enclaves in Florida, Texas, and the Southeast.
Image by Iain, retrieved from: https://commons.wikimedia.org/wiki/File:PRimmigrationchart.png on September 9, 2019. Image is in the public domain.
Hinojosa J. Two Sides of the Coin of Puerto Rican Migration: Depopulation in Puerto Rico and the Redefinition of the Diaspora. Centro Journal. https://www.questia.com/library/journal/1P4-2196369225/two-sides-of-the-coin-of-puerto-rican-migration-depopulation. Published October 1, 2018. Accessed August 13, 2019.
Puerto Ricans in the United States, 2014. Centro de Estudios Puertorriqueños. https://centropr.hunter.cuny.edu/research/data-center/data-sheets/puerto-ricans-united-states-2014. Published 2016. Accessed August 13, 2019.
Sanchez Korrol V. THE STORY OF U.S. PUERTO RICANS - PART ONE. Centro de Estudios Puertorriqueños. http://centroweb.hunter.cuny.edu/education/puerto-rican-studies/story-us-puerto-ricans-part-one. Published 2016. Accessed August 8, 2019.

## Slide 21
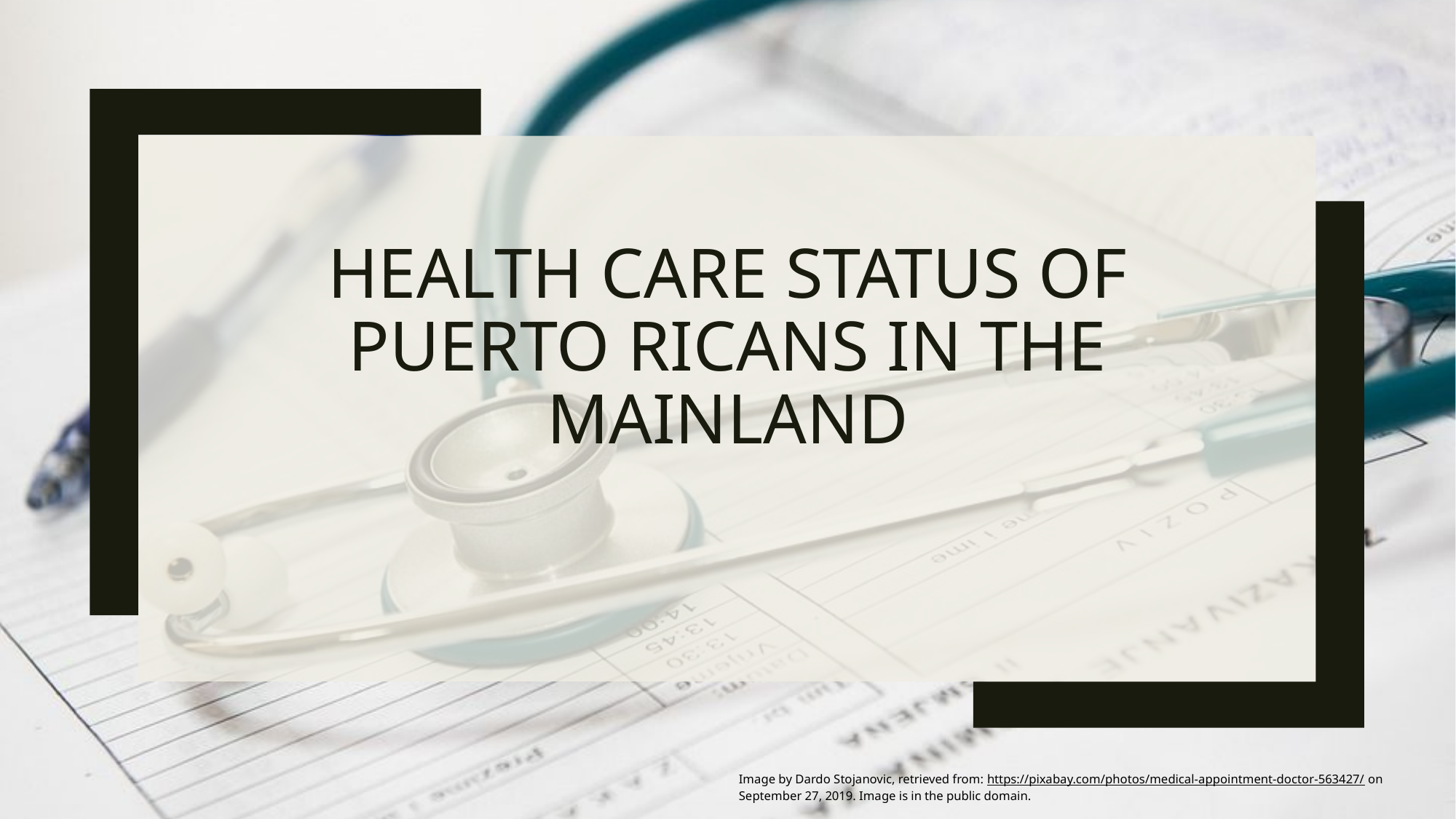

# Health Care Status of Puerto Ricans in the Mainland
Image by Dardo Stojanovic, retrieved from: https://pixabay.com/photos/medical-appointment-doctor-563427/ on September 27, 2019. Image is in the public domain.

## Slide 22
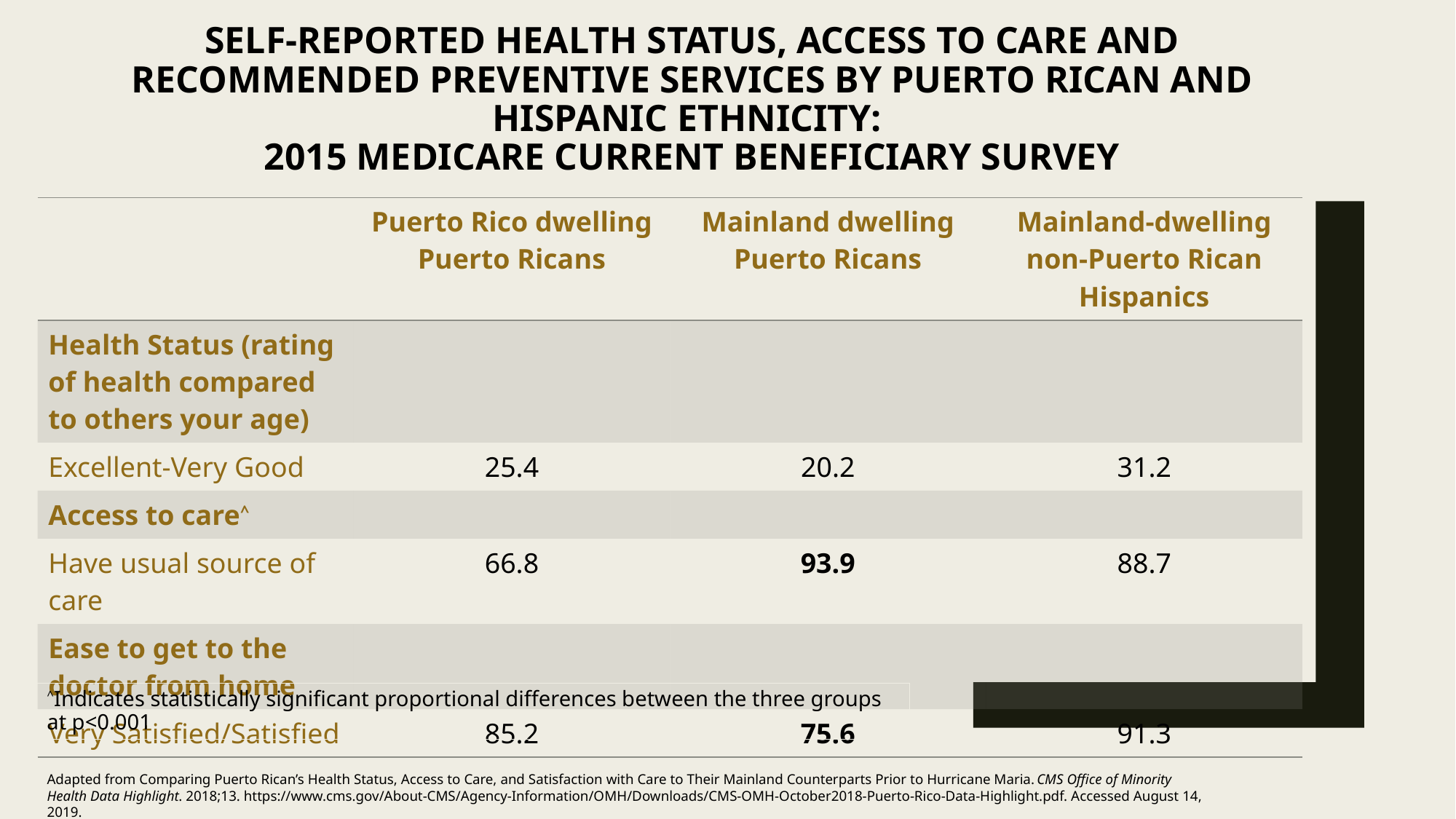

# Self-Reported Health Status, Access to Care and Recommended Preventive Services by Puerto Rican and Hispanic Ethnicity: 2015 Medicare Current Beneficiary Survey
| | Puerto Rico dwelling Puerto Ricans | Mainland dwelling Puerto Ricans | Mainland-dwelling non-Puerto Rican Hispanics |
| --- | --- | --- | --- |
| Health Status (rating of health compared to others your age) | | | |
| Excellent-Very Good | 25.4 | 20.2 | 31.2 |
| Access to care^ | | | |
| Have usual source of care | 66.8 | 93.9 | 88.7 |
| Ease to get to the doctor from home | | | |
| Very Satisfied/Satisfied | 85.2 | 75.6 | 91.3 |
^Indicates statistically significant proportional differences between the three groups at p<0.001
Adapted from Comparing Puerto Rican’s Health Status, Access to Care, and Satisfaction with Care to Their Mainland Counterparts Prior to Hurricane Maria. CMS Office of Minority Health Data Highlight. 2018;13. https://www.cms.gov/About-CMS/Agency-Information/OMH/Downloads/CMS-OMH-October2018-Puerto-Rico-Data-Highlight.pdf. Accessed August 14, 2019.

## Slide 23
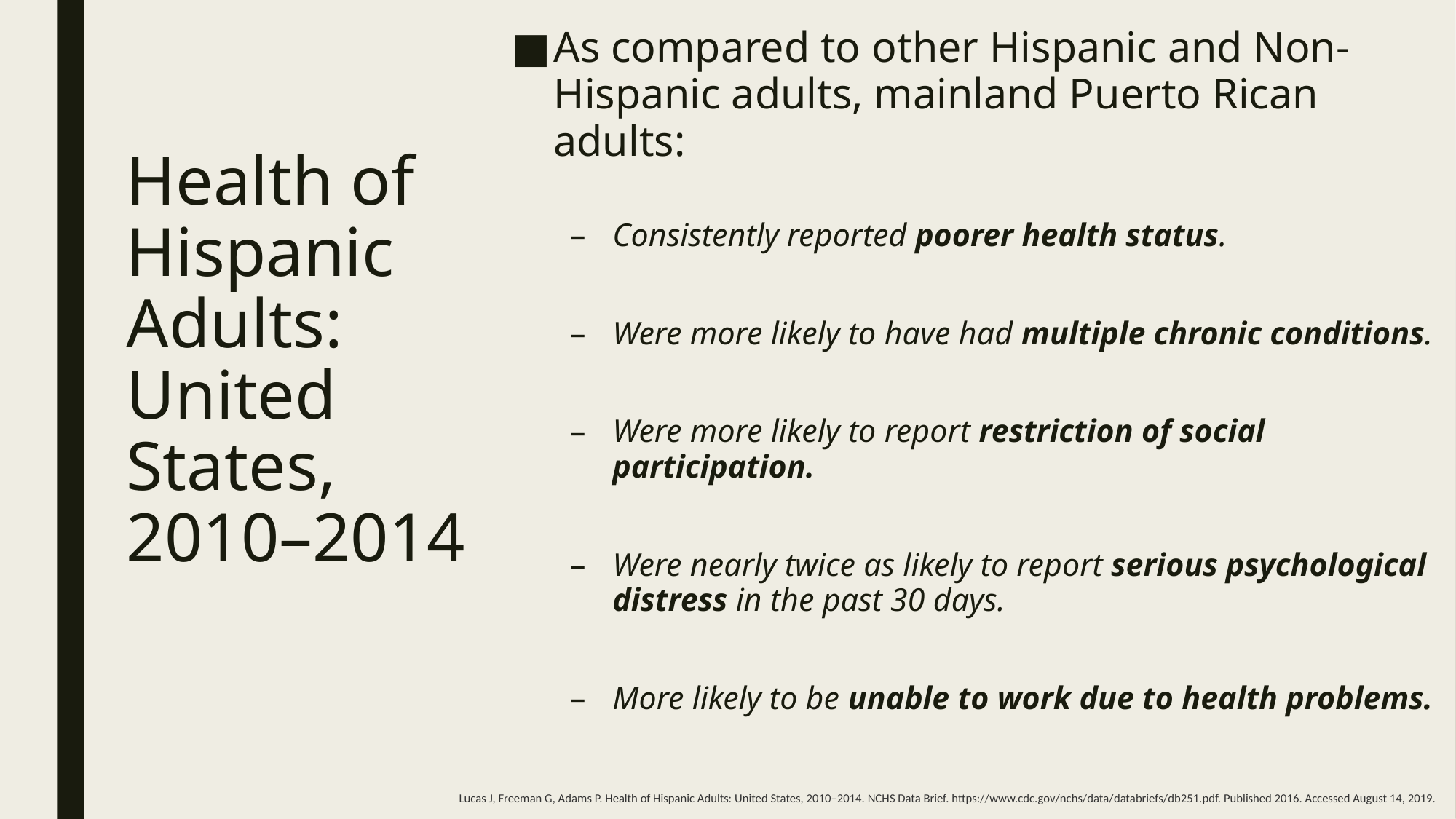

As compared to other Hispanic and Non- Hispanic adults, mainland Puerto Rican adults:
Consistently reported poorer health status.
Were more likely to have had multiple chronic conditions.
Were more likely to report restriction of social participation.
Were nearly twice as likely to report serious psychological distress in the past 30 days.
More likely to be unable to work due to health problems.
# Health of Hispanic Adults: United States, 2010–2014
Lucas J, Freeman G, Adams P. Health of Hispanic Adults: United States, 2010–2014. NCHS Data Brief. https://www.cdc.gov/nchs/data/databriefs/db251.pdf. Published 2016. Accessed August 14, 2019.

## Slide 24
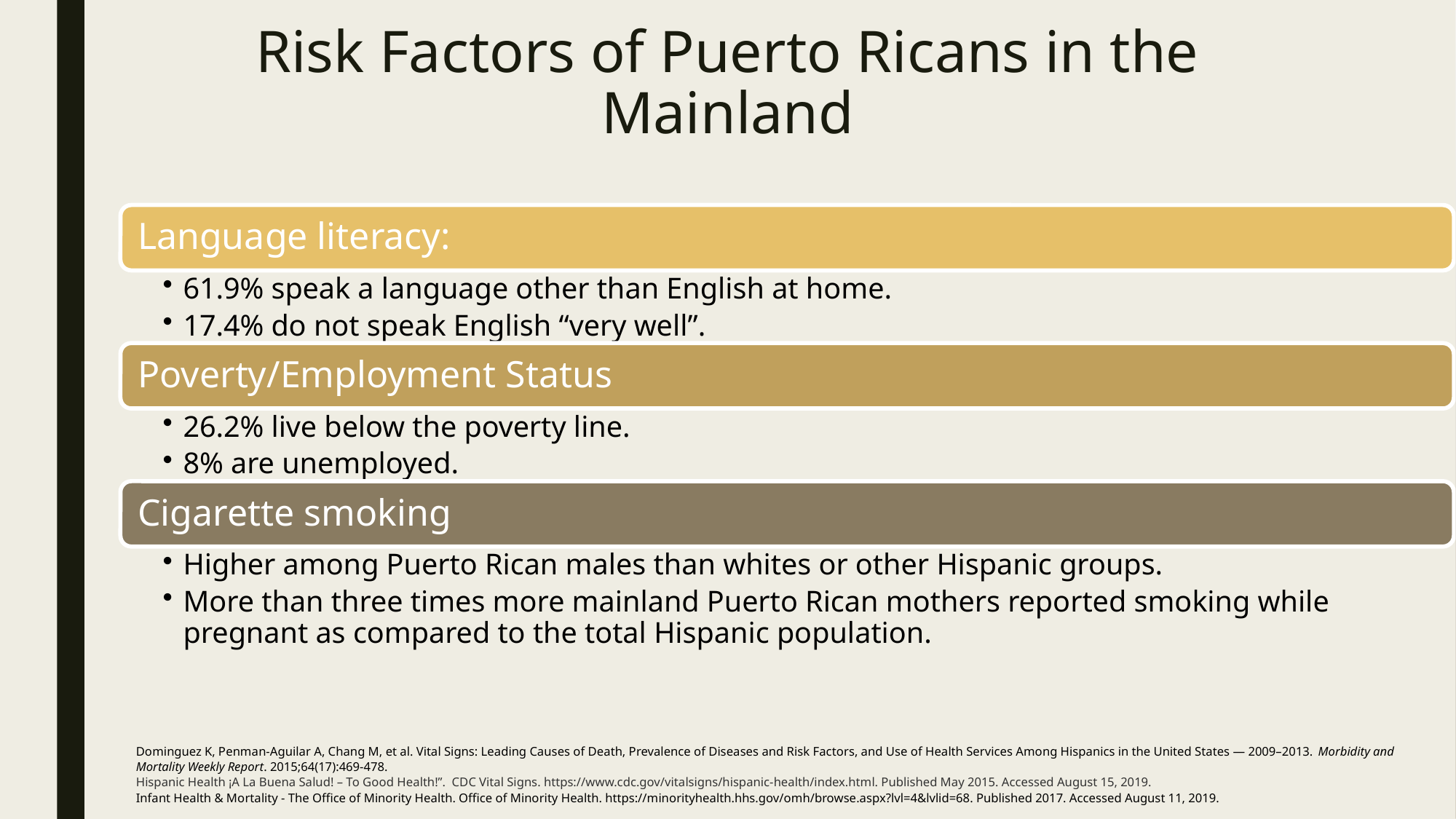

# Risk Factors of Puerto Ricans in the Mainland
Dominguez K, Penman-Aguilar A, Chang M, et al. Vital Signs: Leading Causes of Death, Prevalence of Diseases and Risk Factors, and Use of Health Services Among Hispanics in the United States — 2009–2013. Morbidity and Mortality Weekly Report. 2015;64(17):469-478.
Hispanic Health ¡A La Buena Salud! – To Good Health!”. CDC Vital Signs. https://www.cdc.gov/vitalsigns/hispanic-health/index.html. Published May 2015. Accessed August 15, 2019.
Infant Health & Mortality - The Office of Minority Health. Office of Minority Health. https://minorityhealth.hhs.gov/omh/browse.aspx?lvl=4&lvlid=68. Published 2017. Accessed August 11, 2019.

## Slide 25
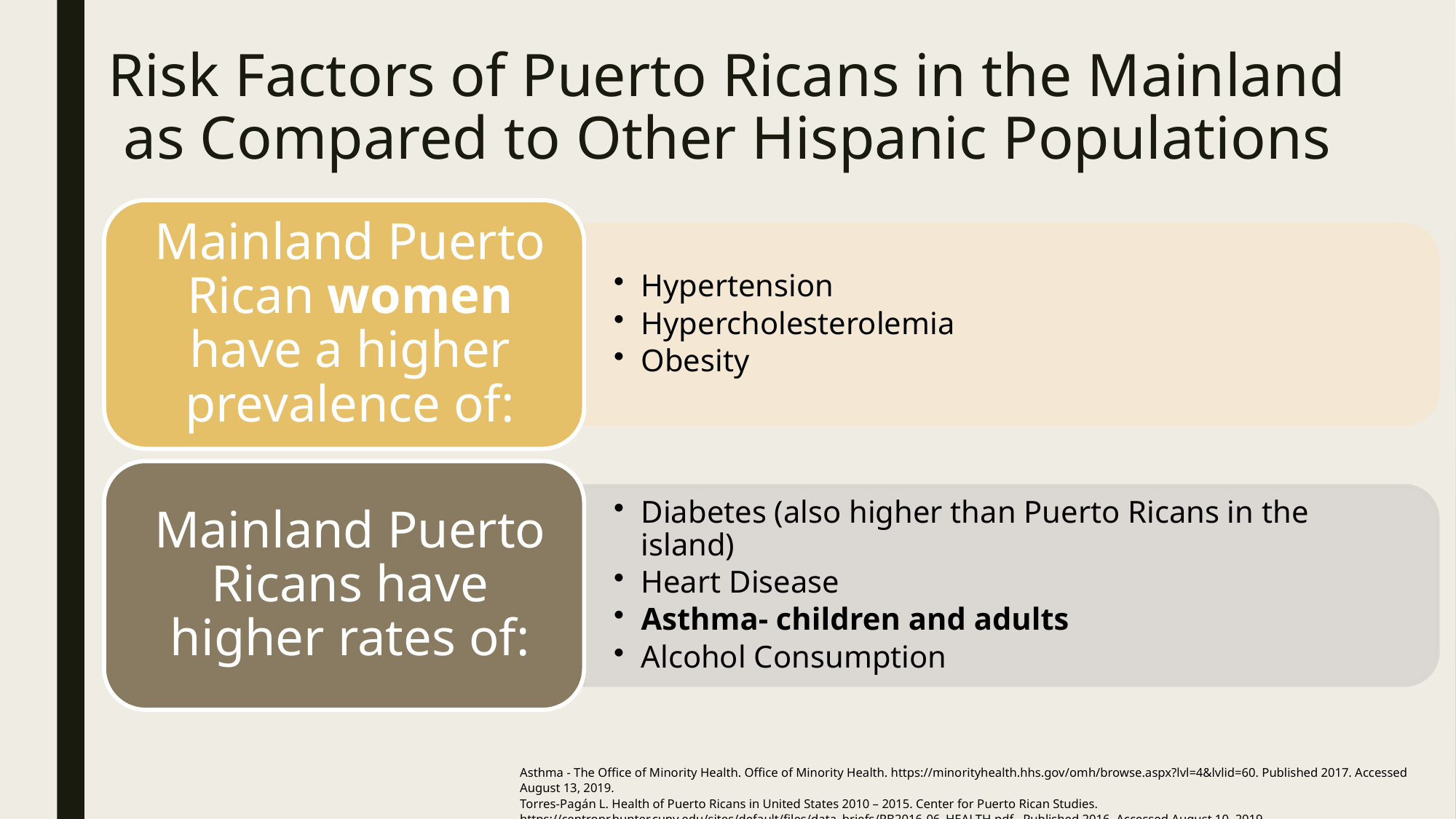

# Risk Factors of Puerto Ricans in the Mainland as Compared to Other Hispanic Populations
Asthma - The Office of Minority Health. Office of Minority Health. https://minorityhealth.hhs.gov/omh/browse.aspx?lvl=4&lvlid=60. Published 2017. Accessed August 13, 2019.
Torres-Pagán L. Health of Puerto Ricans in United States 2010 – 2015. Center for Puerto Rican Studies. https://centropr.hunter.cuny.edu/sites/default/files/data_briefs/RB2016-06_HEALTH.pdf . Published 2016. Accessed August 10, 2019.

## Slide 26
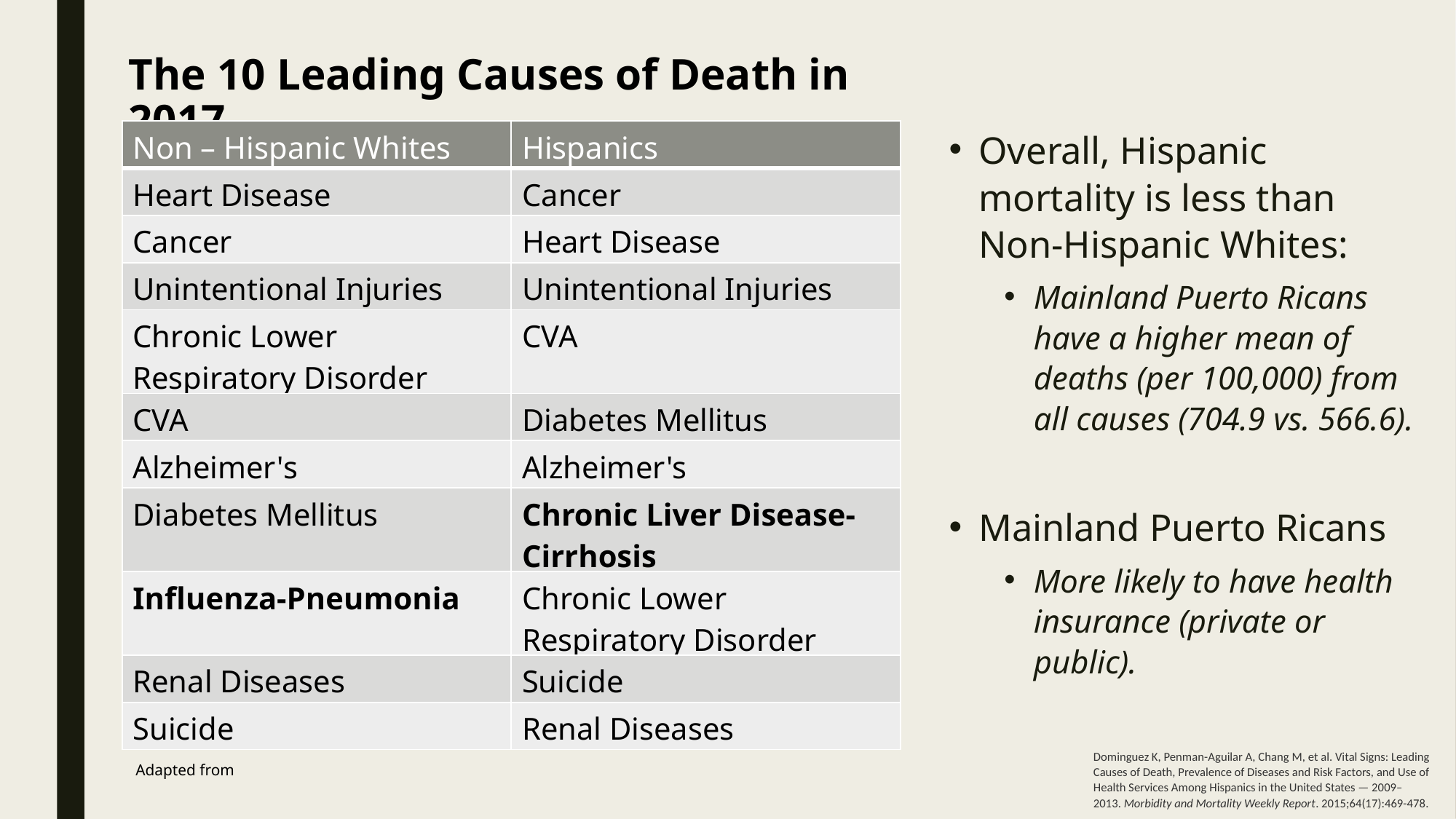

The 10 Leading Causes of Death in 2017
Overall, Hispanic mortality is less than Non-Hispanic Whites:
Mainland Puerto Ricans have a higher mean of deaths (per 100,000) from all causes (704.9 vs. 566.6).
Mainland Puerto Ricans
More likely to have health insurance (private or public).
| Non – Hispanic Whites | Hispanics |
| --- | --- |
| Heart Disease | Cancer |
| Cancer | Heart Disease |
| Unintentional Injuries | Unintentional Injuries |
| Chronic Lower Respiratory Disorder | CVA |
| CVA | Diabetes Mellitus |
| Alzheimer's | Alzheimer's |
| Diabetes Mellitus | Chronic Liver Disease-Cirrhosis |
| Influenza-Pneumonia | Chronic Lower Respiratory Disorder |
| Renal Diseases | Suicide |
| Suicide | Renal Diseases |
Dominguez K, Penman-Aguilar A, Chang M, et al. Vital Signs: Leading Causes of Death, Prevalence of Diseases and Risk Factors, and Use of Health Services Among Hispanics in the United States — 2009–2013. Morbidity and Mortality Weekly Report. 2015;64(17):469-478.
Adapted from

## Slide 27
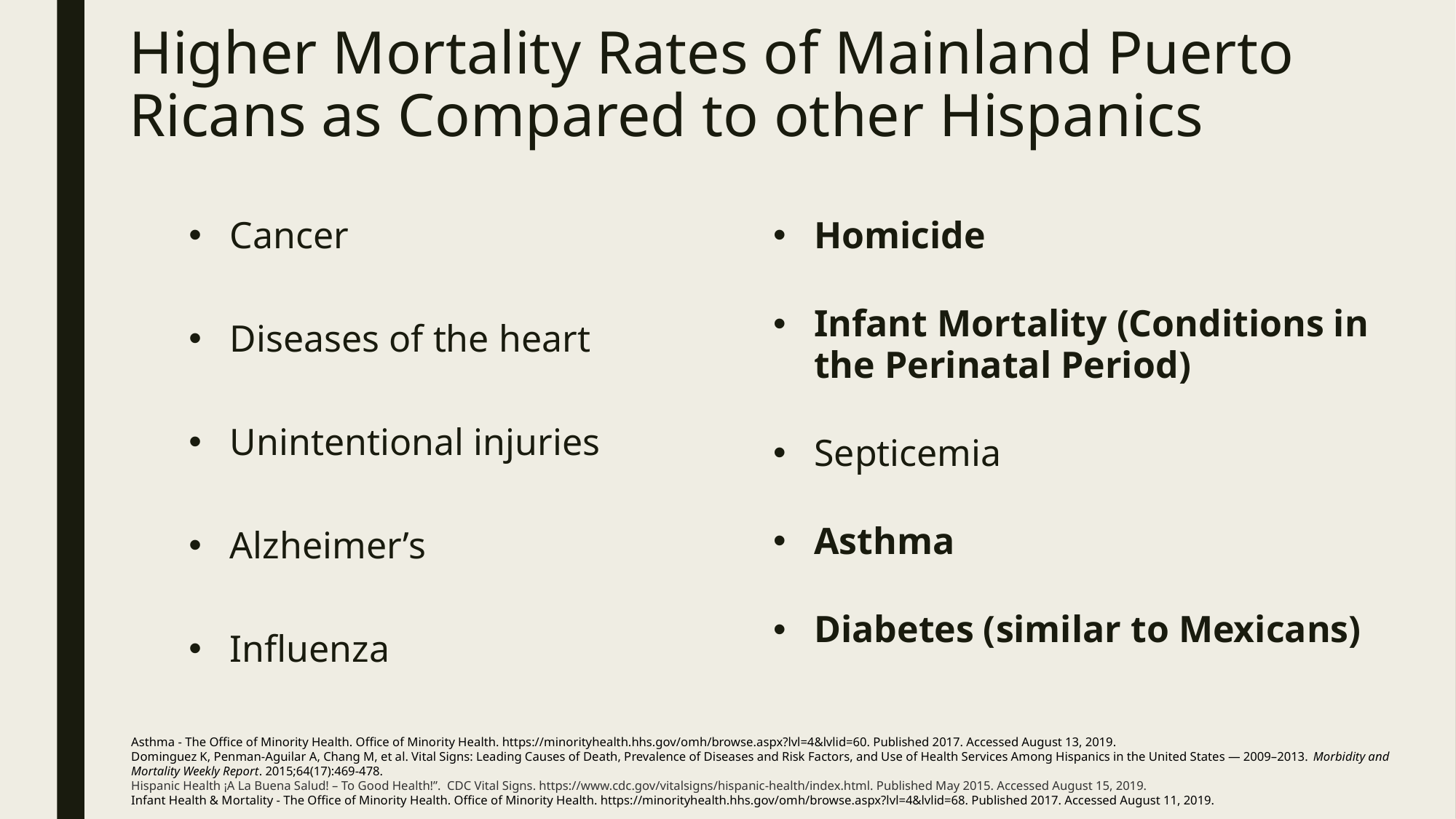

# Higher Mortality Rates of Mainland Puerto Ricans as Compared to other Hispanics
Cancer
Diseases of the heart
Unintentional injuries
Alzheimer’s
Influenza
Homicide
Infant Mortality (Conditions in the Perinatal Period)
Septicemia
Asthma
Diabetes (similar to Mexicans)
Asthma - The Office of Minority Health. Office of Minority Health. https://minorityhealth.hhs.gov/omh/browse.aspx?lvl=4&lvlid=60. Published 2017. Accessed August 13, 2019.
Dominguez K, Penman-Aguilar A, Chang M, et al. Vital Signs: Leading Causes of Death, Prevalence of Diseases and Risk Factors, and Use of Health Services Among Hispanics in the United States — 2009–2013. Morbidity and Mortality Weekly Report. 2015;64(17):469-478.
Hispanic Health ¡A La Buena Salud! – To Good Health!”. CDC Vital Signs. https://www.cdc.gov/vitalsigns/hispanic-health/index.html. Published May 2015. Accessed August 15, 2019.
Infant Health & Mortality - The Office of Minority Health. Office of Minority Health. https://minorityhealth.hhs.gov/omh/browse.aspx?lvl=4&lvlid=68. Published 2017. Accessed August 11, 2019.

## Slide 28
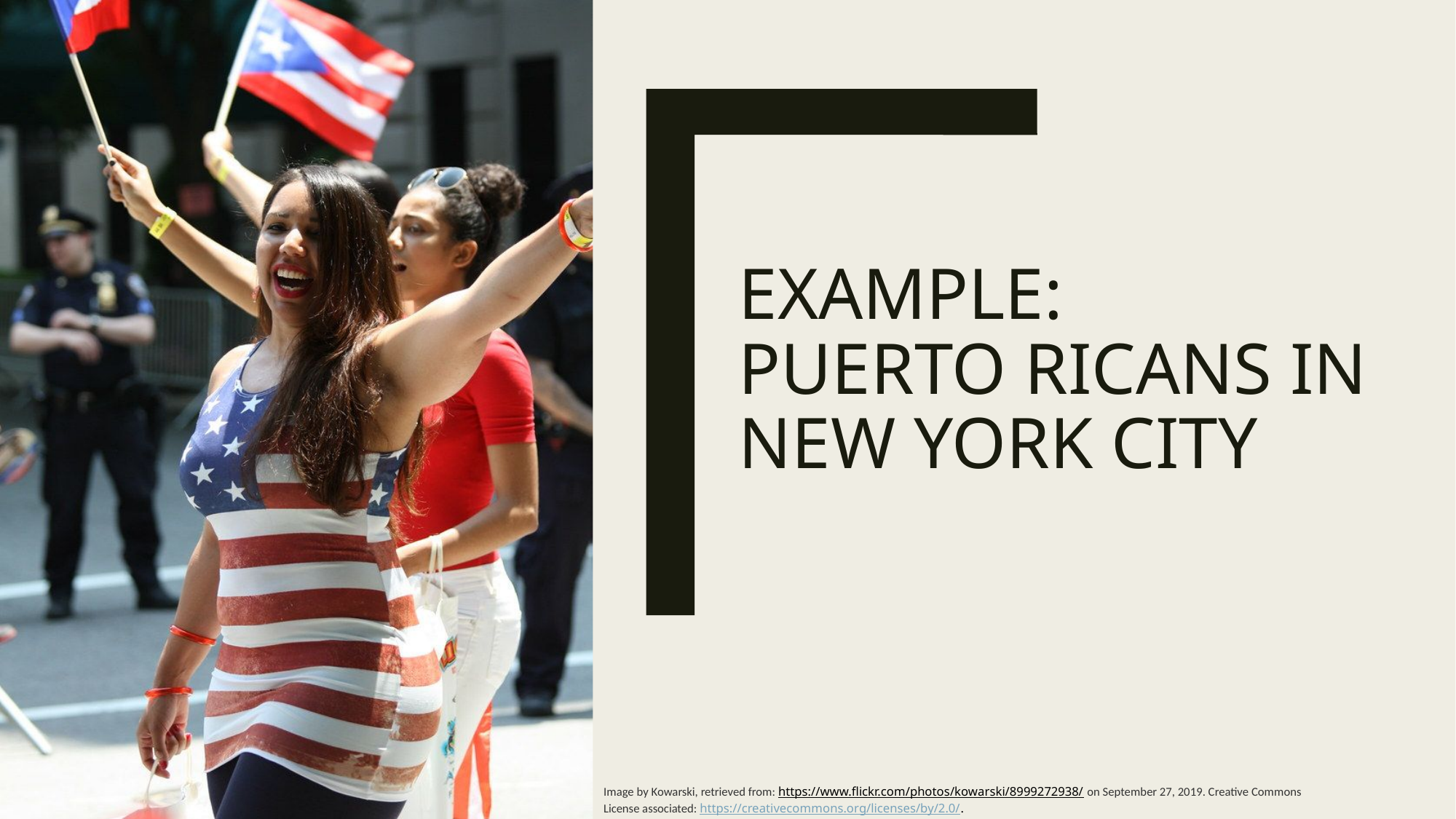

# Example: Puerto ricans in new york city
Image by Kowarski, retrieved from: https://www.flickr.com/photos/kowarski/8999272938/ on September 27, 2019. Creative Commons License associated: https://creativecommons.org/licenses/by/2.0/.

## Slide 29
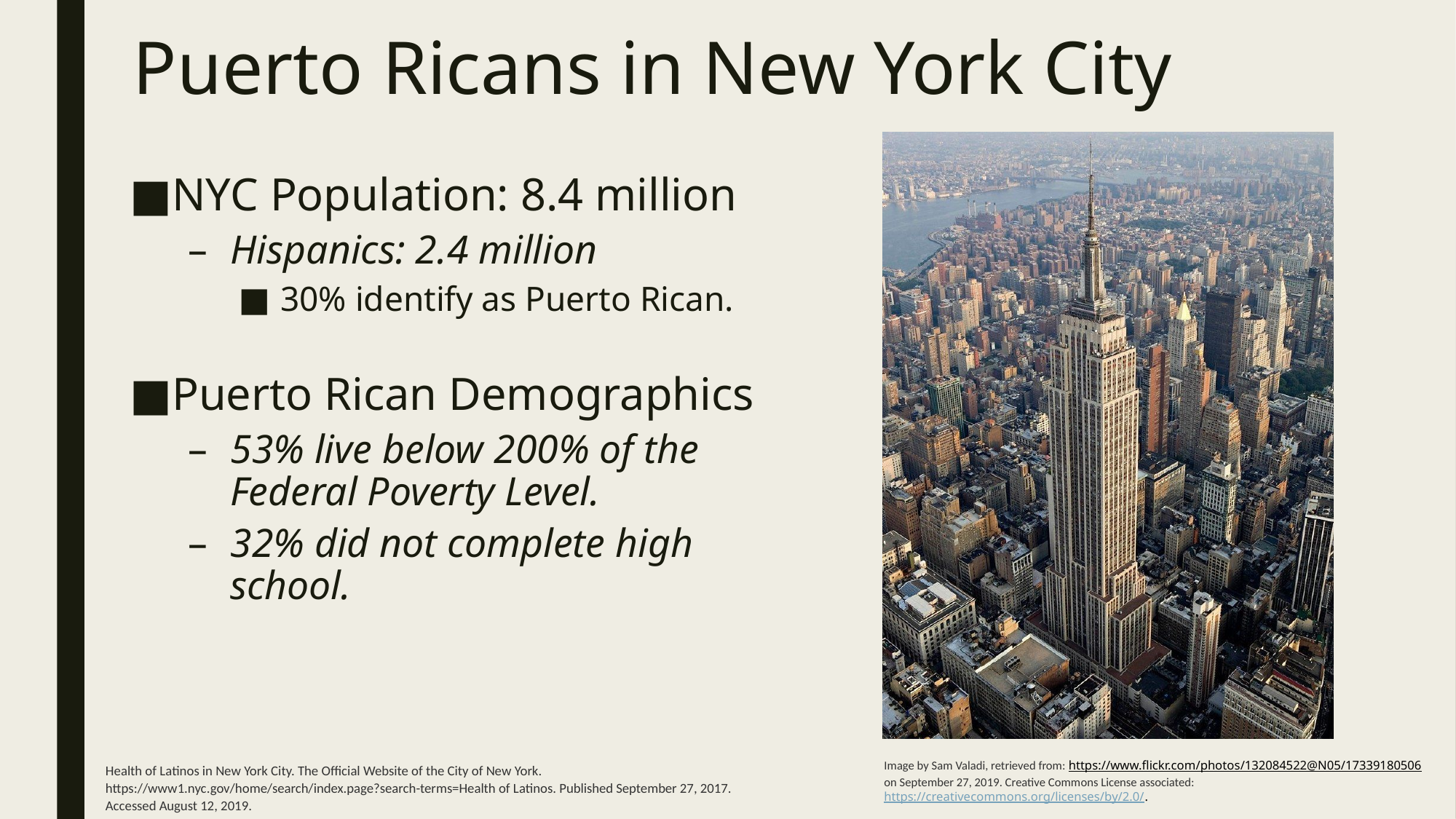

# Puerto Ricans in New York City
NYC Population: 8.4 million
Hispanics: 2.4 million
30% identify as Puerto Rican.
Puerto Rican Demographics
53% live below 200% of the Federal Poverty Level.
32% did not complete high school.
Image by Sam Valadi, retrieved from: https://www.flickr.com/photos/132084522@N05/17339180506 on September 27, 2019. Creative Commons License associated: https://creativecommons.org/licenses/by/2.0/.
Health of Latinos in New York City. The Official Website of the City of New York. https://www1.nyc.gov/home/search/index.page?search-terms=Health of Latinos. Published September 27, 2017. Accessed August 12, 2019.

## Slide 30
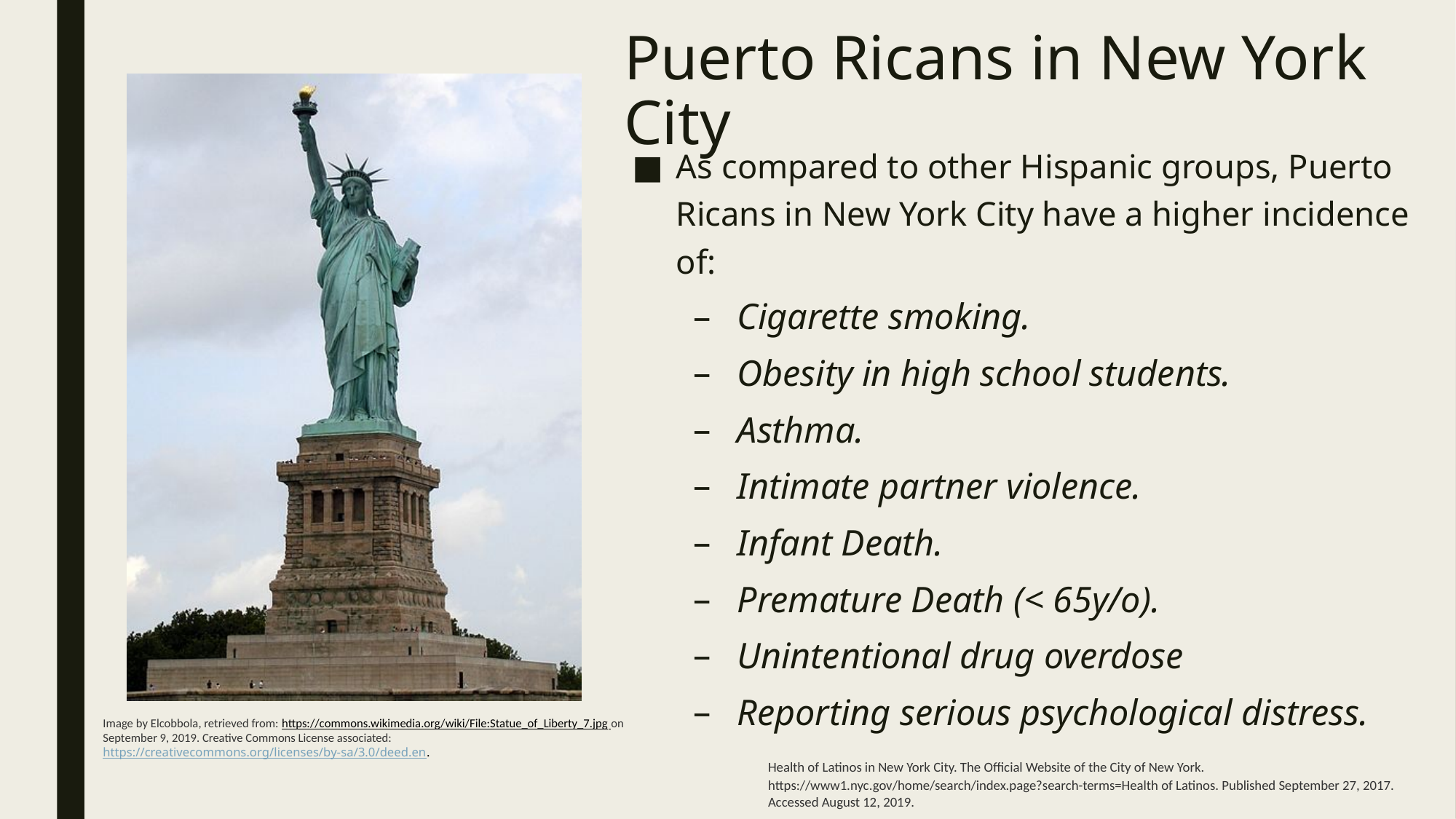

Puerto Ricans in New York City
As compared to other Hispanic groups, Puerto Ricans in New York City have a higher incidence of:
Cigarette smoking.
Obesity in high school students.
Asthma.
Intimate partner violence.
Infant Death.
Premature Death (< 65y/o).
Unintentional drug overdose
Reporting serious psychological distress.
Image by Elcobbola, retrieved from: https://commons.wikimedia.org/wiki/File:Statue_of_Liberty_7.jpg on September 9, 2019. Creative Commons License associated: https://creativecommons.org/licenses/by-sa/3.0/deed.en.
Health of Latinos in New York City. The Official Website of the City of New York. https://www1.nyc.gov/home/search/index.page?search-terms=Health of Latinos. Published September 27, 2017. Accessed August 12, 2019.

## Slide 31
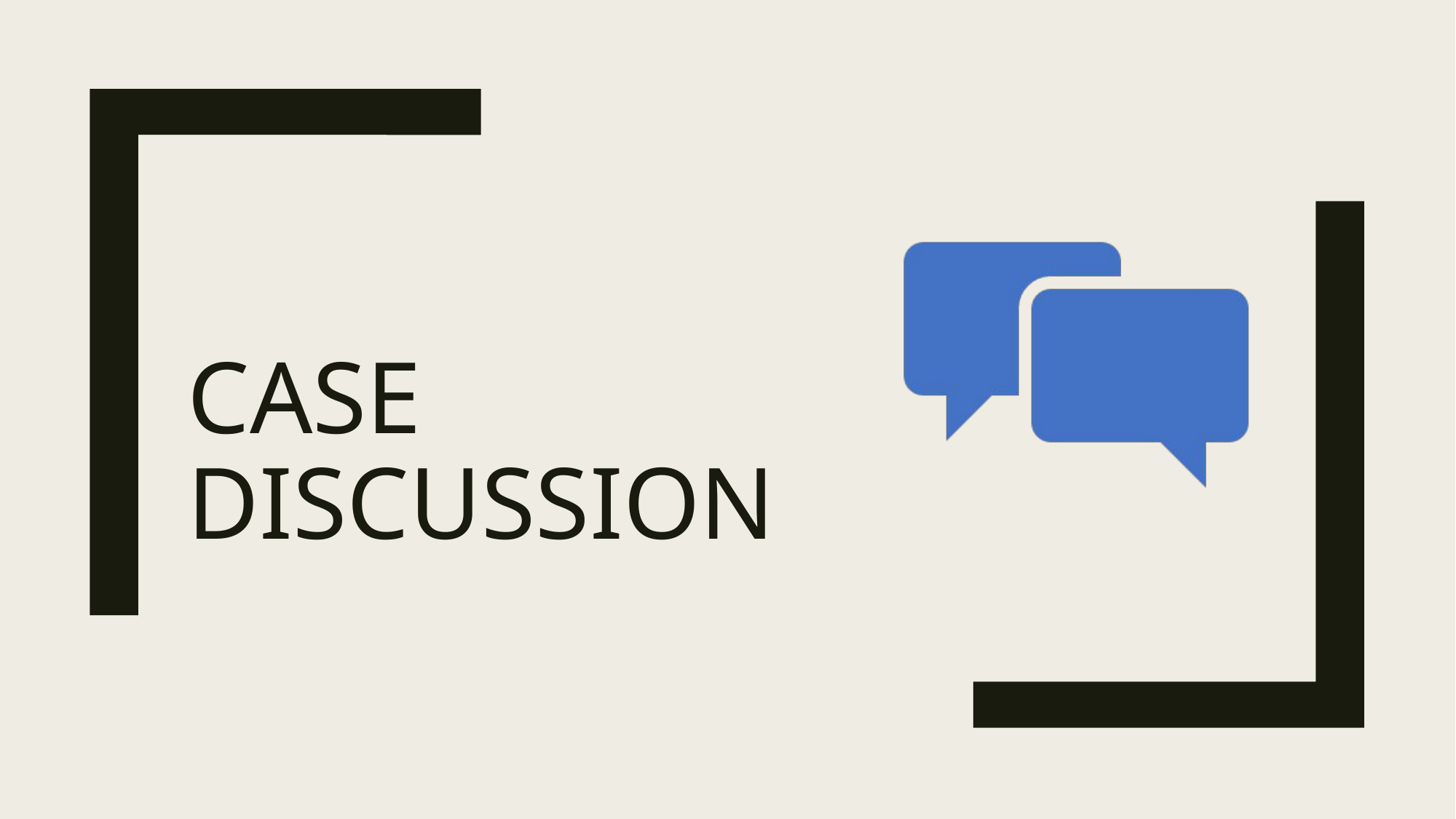

# Case discussion

## Slide 32
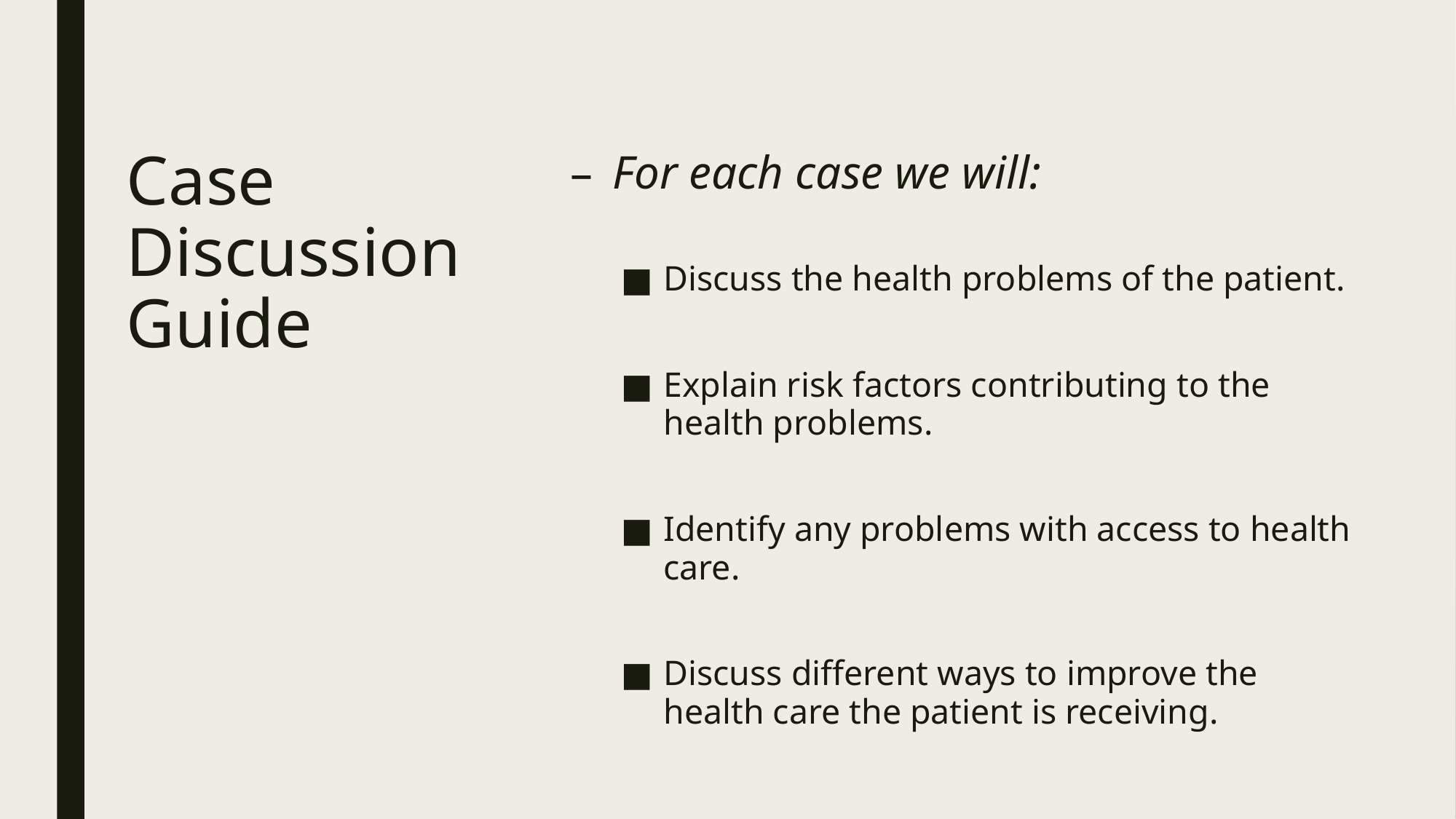

For each case we will:
Discuss the health problems of the patient.
Explain risk factors contributing to the health problems.
Identify any problems with access to health care.
Discuss different ways to improve the health care the patient is receiving.
# Case Discussion Guide

## Slide 33
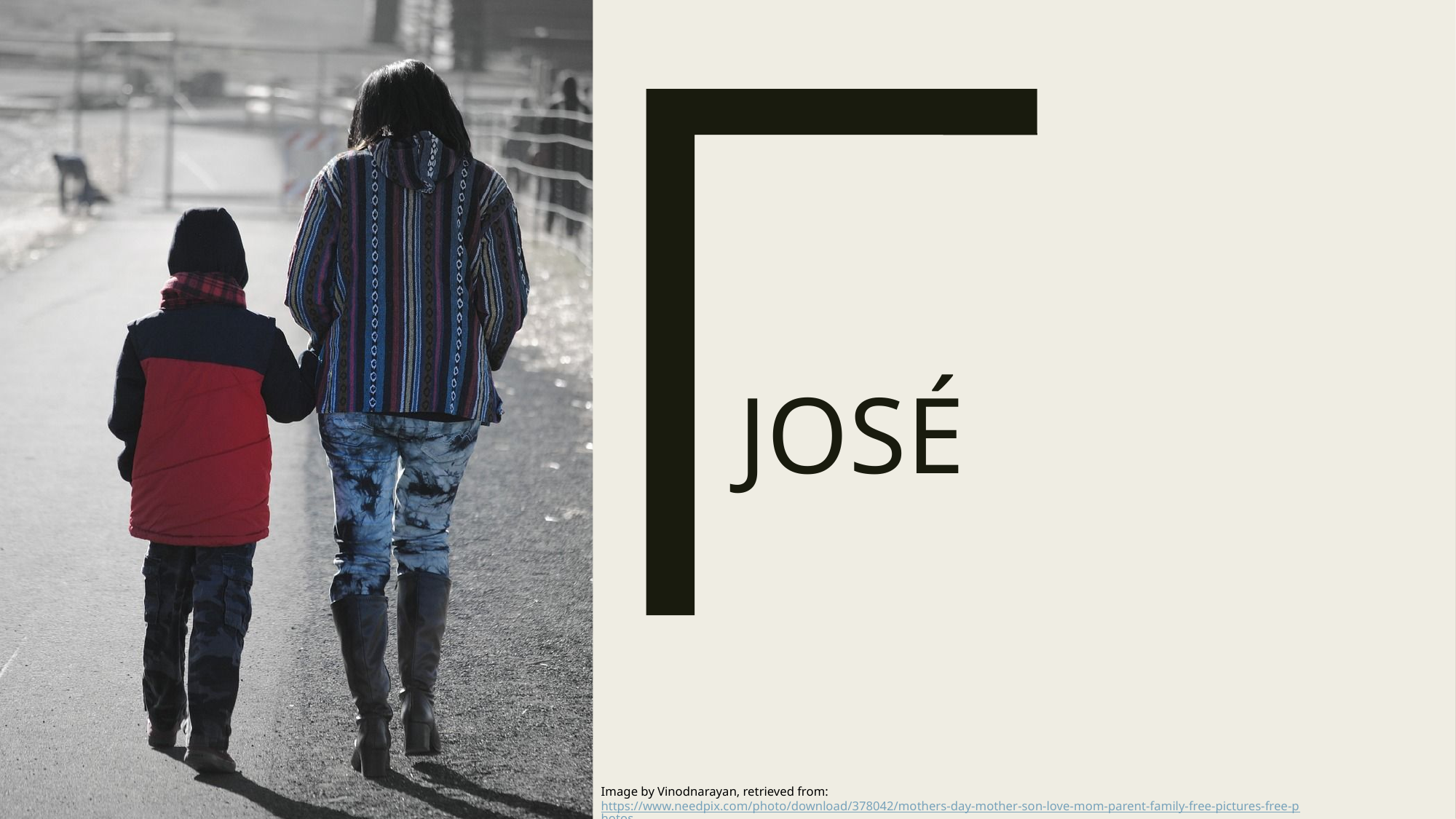

# José
Image by Vinodnarayan, retrieved from: https://www.needpix.com/photo/download/378042/mothers-day-mother-son-love-mom-parent-family-free-pictures-free-photos on September 27,2019. Image is in the public domain.

## Slide 34
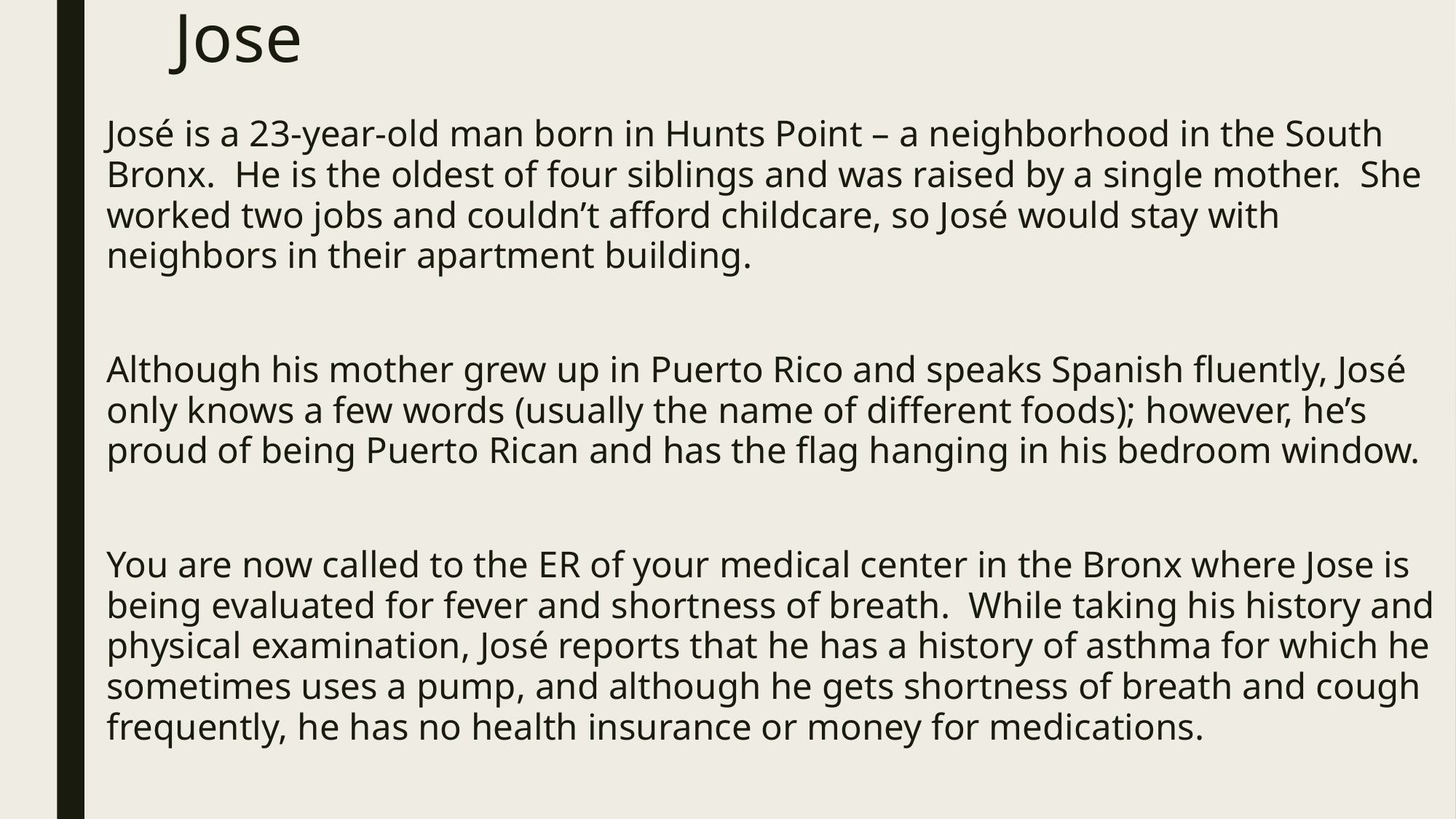

# Jose
José is a 23-year-old man born in Hunts Point – a neighborhood in the South Bronx. He is the oldest of four siblings and was raised by a single mother. She worked two jobs and couldn’t afford childcare, so José would stay with neighbors in their apartment building.
Although his mother grew up in Puerto Rico and speaks Spanish fluently, José only knows a few words (usually the name of different foods); however, he’s proud of being Puerto Rican and has the flag hanging in his bedroom window.
You are now called to the ER of your medical center in the Bronx where Jose is being evaluated for fever and shortness of breath. While taking his history and physical examination, José reports that he has a history of asthma for which he sometimes uses a pump, and although he gets shortness of breath and cough frequently, he has no health insurance or money for medications.

## Slide 35
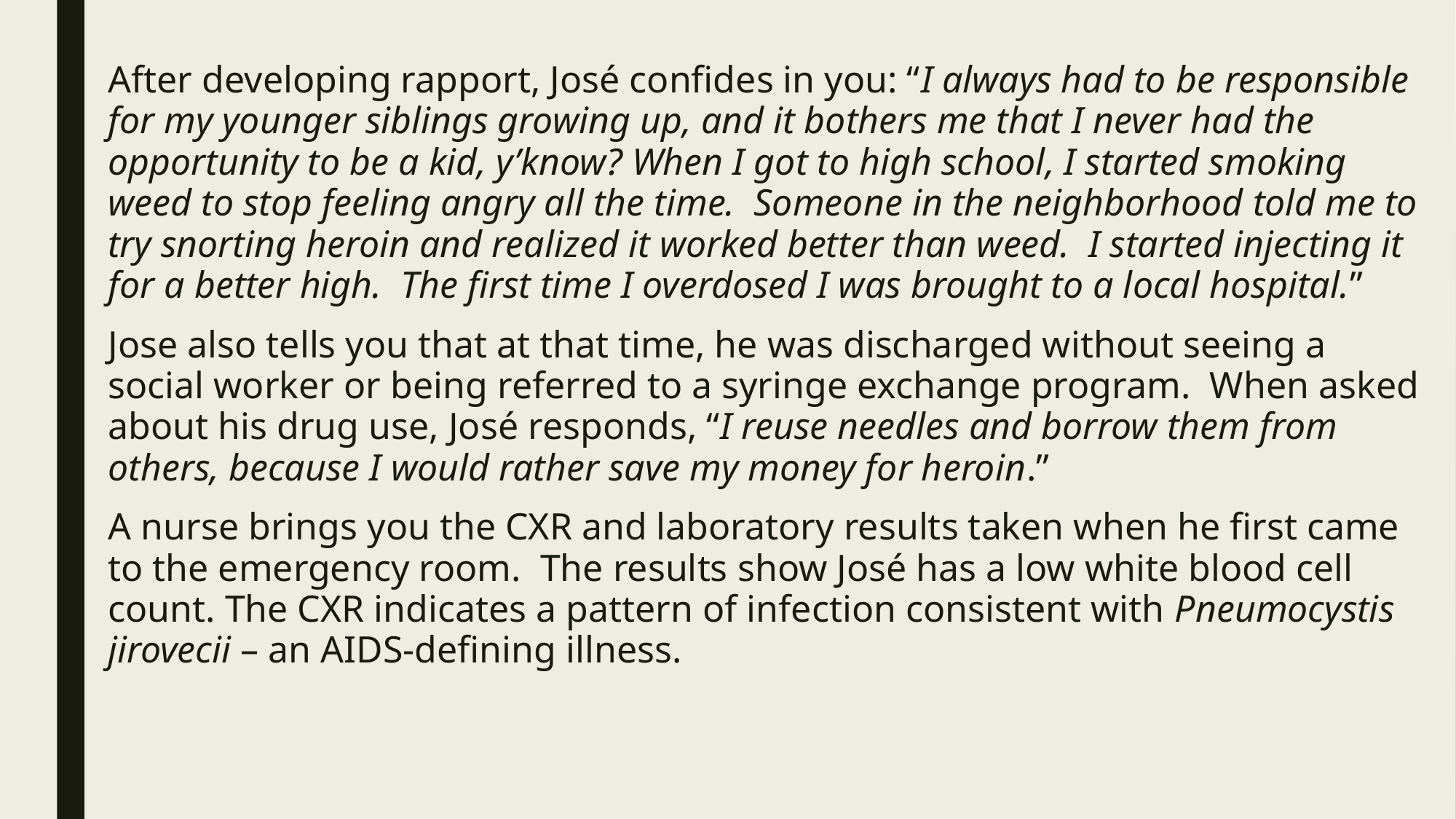

After developing rapport, José confides in you: “I always had to be responsible for my younger siblings growing up, and it bothers me that I never had the opportunity to be a kid, y’know? When I got to high school, I started smoking weed to stop feeling angry all the time. Someone in the neighborhood told me to try snorting heroin and realized it worked better than weed. I started injecting it for a better high. The first time I overdosed I was brought to a local hospital.”
Jose also tells you that at that time, he was discharged without seeing a social worker or being referred to a syringe exchange program. When asked about his drug use, José responds, “I reuse needles and borrow them from others, because I would rather save my money for heroin.”
A nurse brings you the CXR and laboratory results taken when he first came to the emergency room. The results show José has a low white blood cell count. The CXR indicates a pattern of infection consistent with Pneumocystis jirovecii – an AIDS-defining illness.

## Slide 36
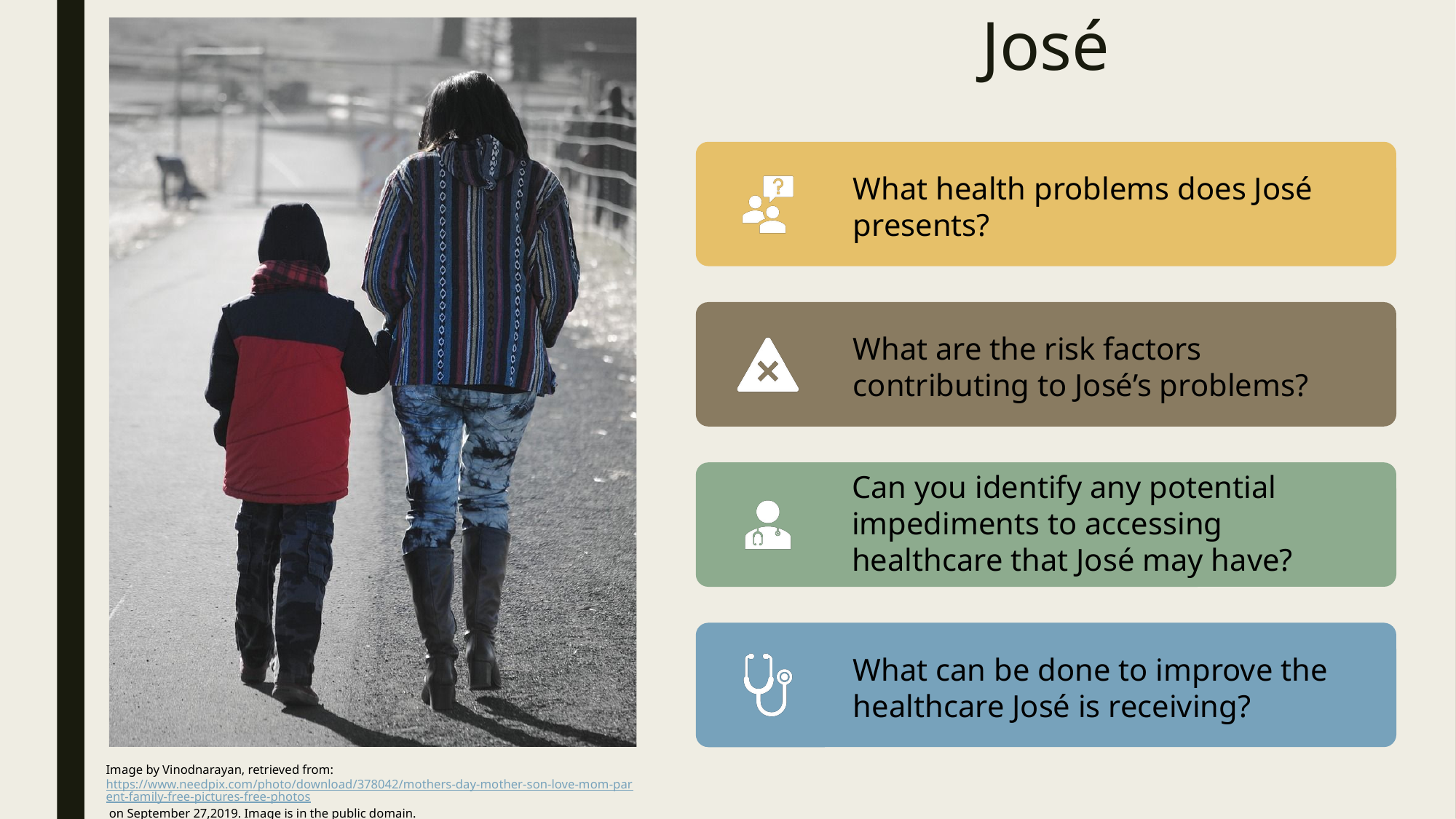

# José
Image by Vinodnarayan, retrieved from: https://www.needpix.com/photo/download/378042/mothers-day-mother-son-love-mom-parent-family-free-pictures-free-photos on September 27,2019. Image is in the public domain.

## Slide 37
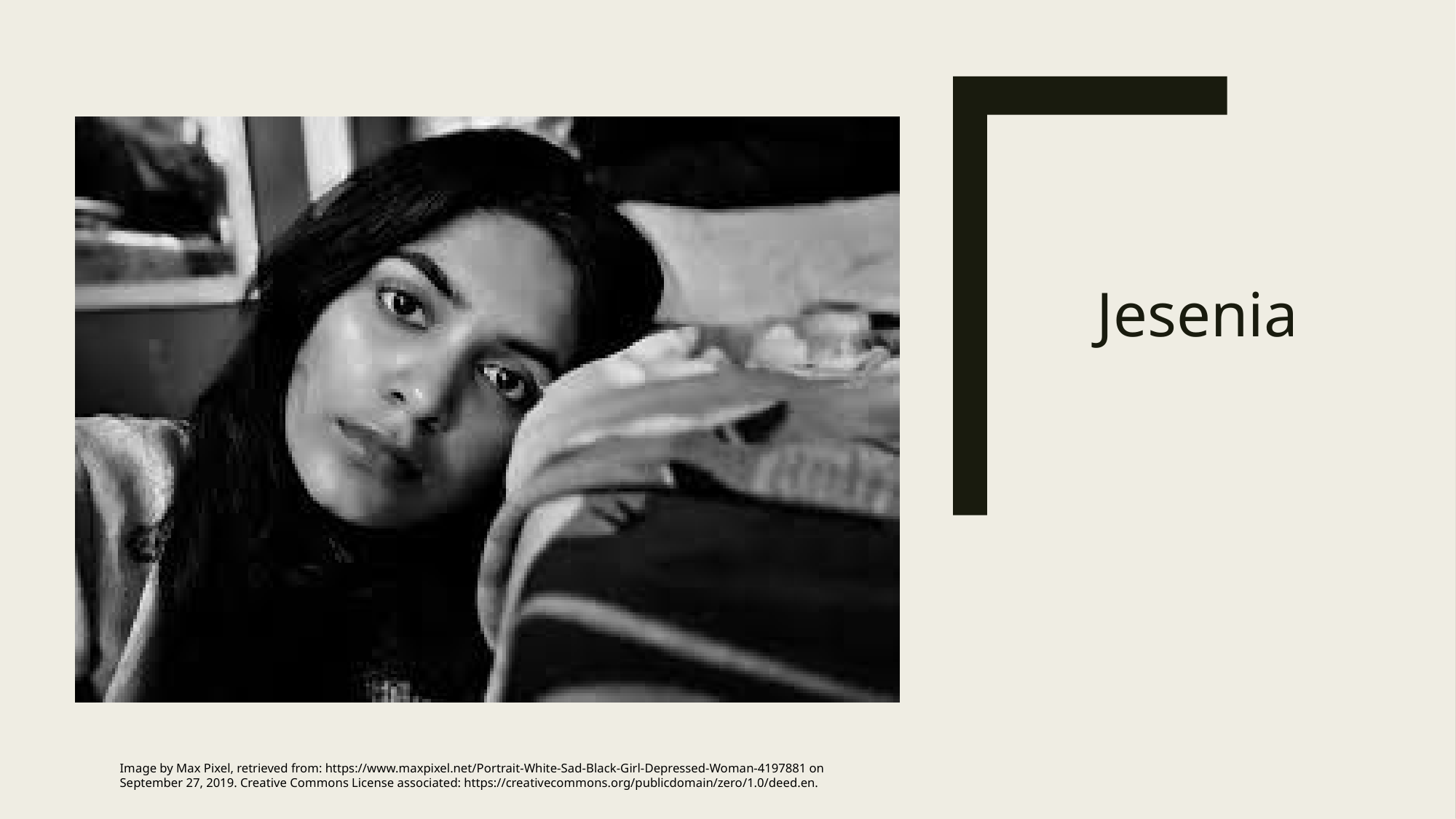

# Jesenia
Image by Max Pixel, retrieved from: https://www.maxpixel.net/Portrait-White-Sad-Black-Girl-Depressed-Woman-4197881 on September 27, 2019. Creative Commons License associated: https://creativecommons.org/publicdomain/zero/1.0/deed.en.

## Slide 38
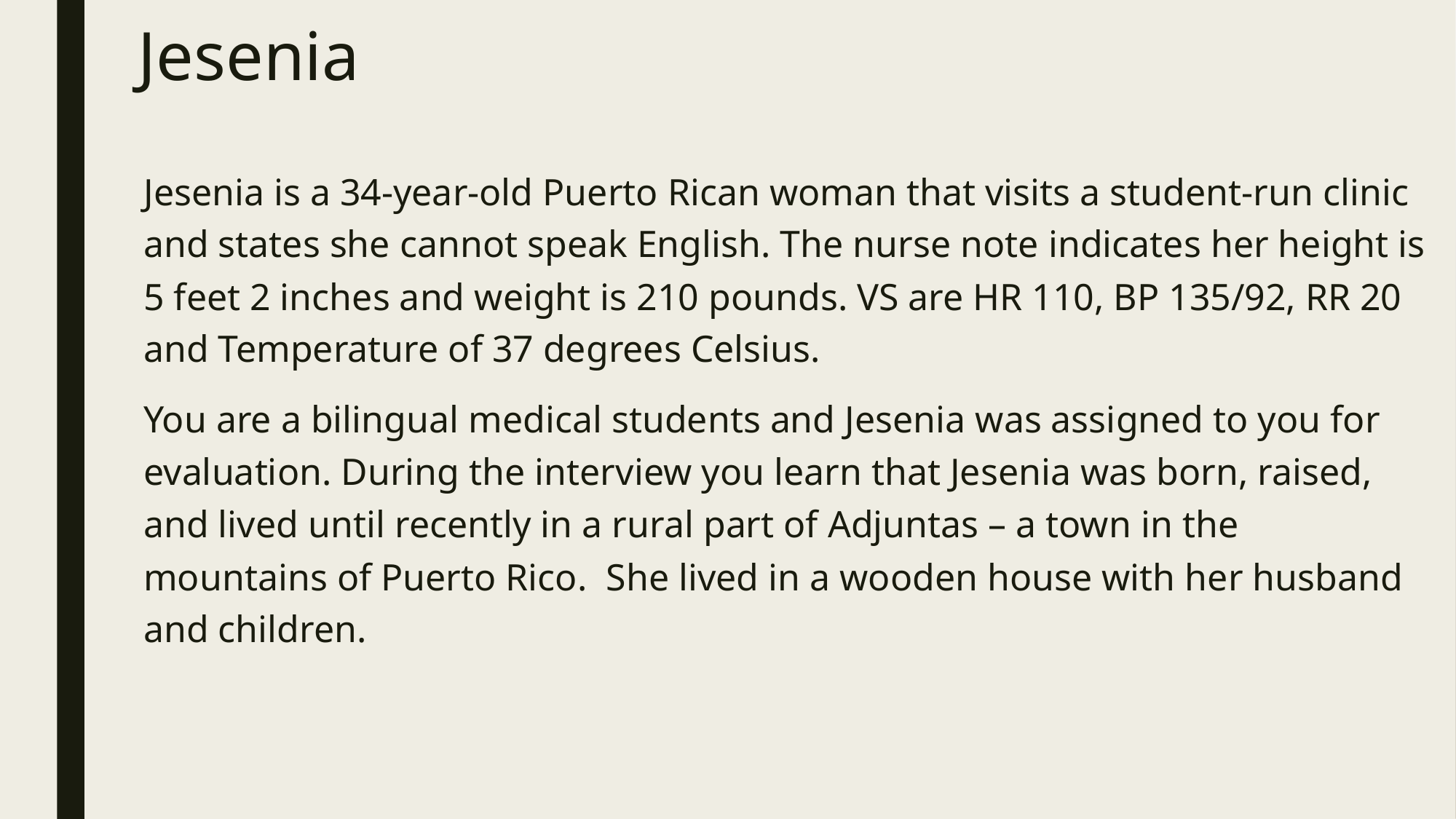

# Jesenia
Jesenia is a 34-year-old Puerto Rican woman that visits a student-run clinic and states she cannot speak English. The nurse note indicates her height is 5 feet 2 inches and weight is 210 pounds. VS are HR 110, BP 135/92, RR 20 and Temperature of 37 degrees Celsius.
You are a bilingual medical students and Jesenia was assigned to you for evaluation. During the interview you learn that Jesenia was born, raised, and lived until recently in a rural part of Adjuntas – a town in the mountains of Puerto Rico. She lived in a wooden house with her husband and children.

## Slide 39
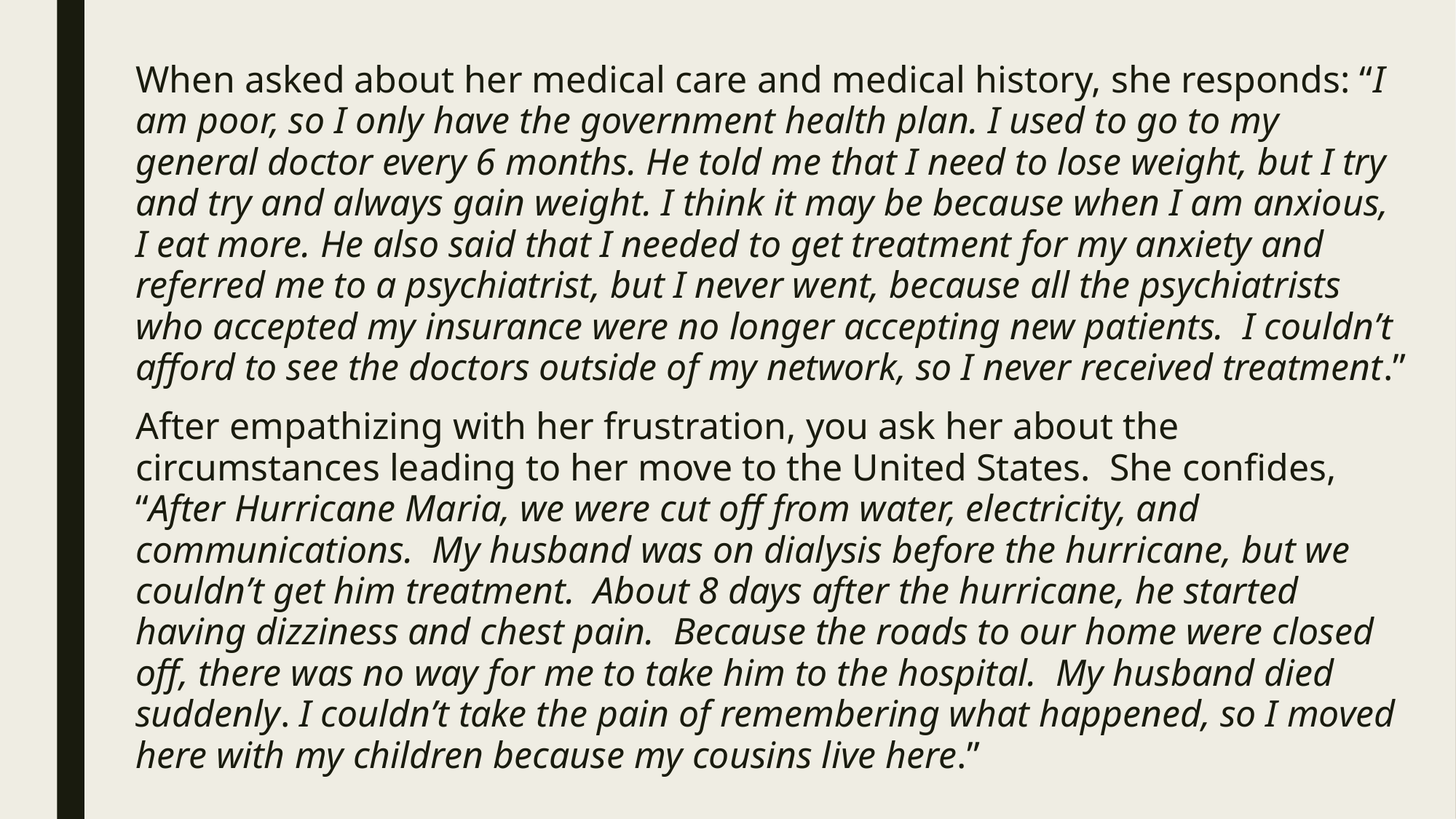

When asked about her medical care and medical history, she responds: “I am poor, so I only have the government health plan. I used to go to my general doctor every 6 months. He told me that I need to lose weight, but I try and try and always gain weight. I think it may be because when I am anxious, I eat more. He also said that I needed to get treatment for my anxiety and referred me to a psychiatrist, but I never went, because all the psychiatrists who accepted my insurance were no longer accepting new patients. I couldn’t afford to see the doctors outside of my network, so I never received treatment.”
After empathizing with her frustration, you ask her about the circumstances leading to her move to the United States. She confides, “After Hurricane Maria, we were cut off from water, electricity, and communications. My husband was on dialysis before the hurricane, but we couldn’t get him treatment. About 8 days after the hurricane, he started having dizziness and chest pain. Because the roads to our home were closed off, there was no way for me to take him to the hospital. My husband died suddenly. I couldn’t take the pain of remembering what happened, so I moved here with my children because my cousins live here.”

## Slide 40
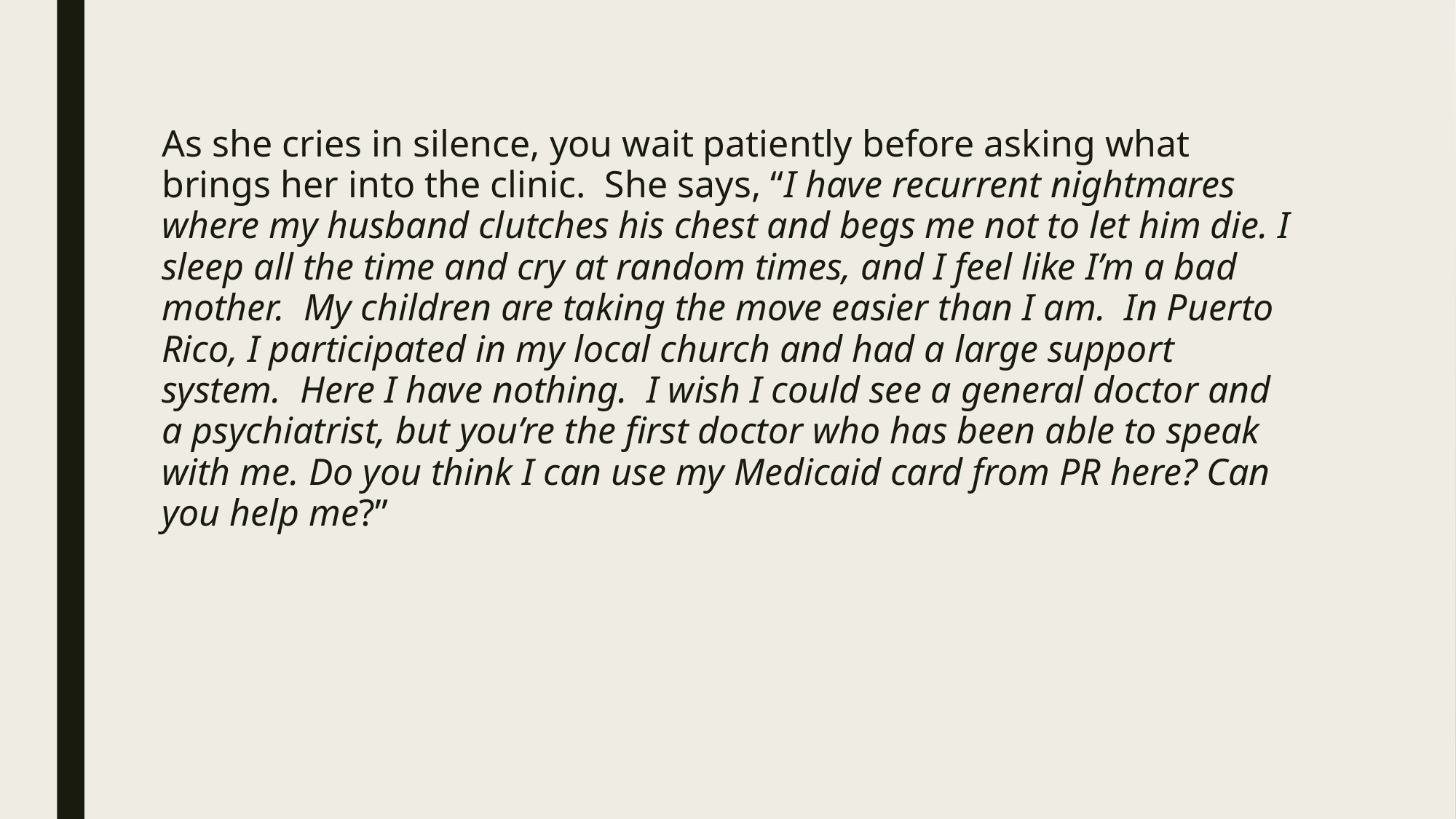

As she cries in silence, you wait patiently before asking what brings her into the clinic. She says, “I have recurrent nightmares where my husband clutches his chest and begs me not to let him die. I sleep all the time and cry at random times, and I feel like I’m a bad mother. My children are taking the move easier than I am. In Puerto Rico, I participated in my local church and had a large support system. Here I have nothing. I wish I could see a general doctor and a psychiatrist, but you’re the first doctor who has been able to speak with me. Do you think I can use my Medicaid card from PR here? Can you help me?”

## Slide 41
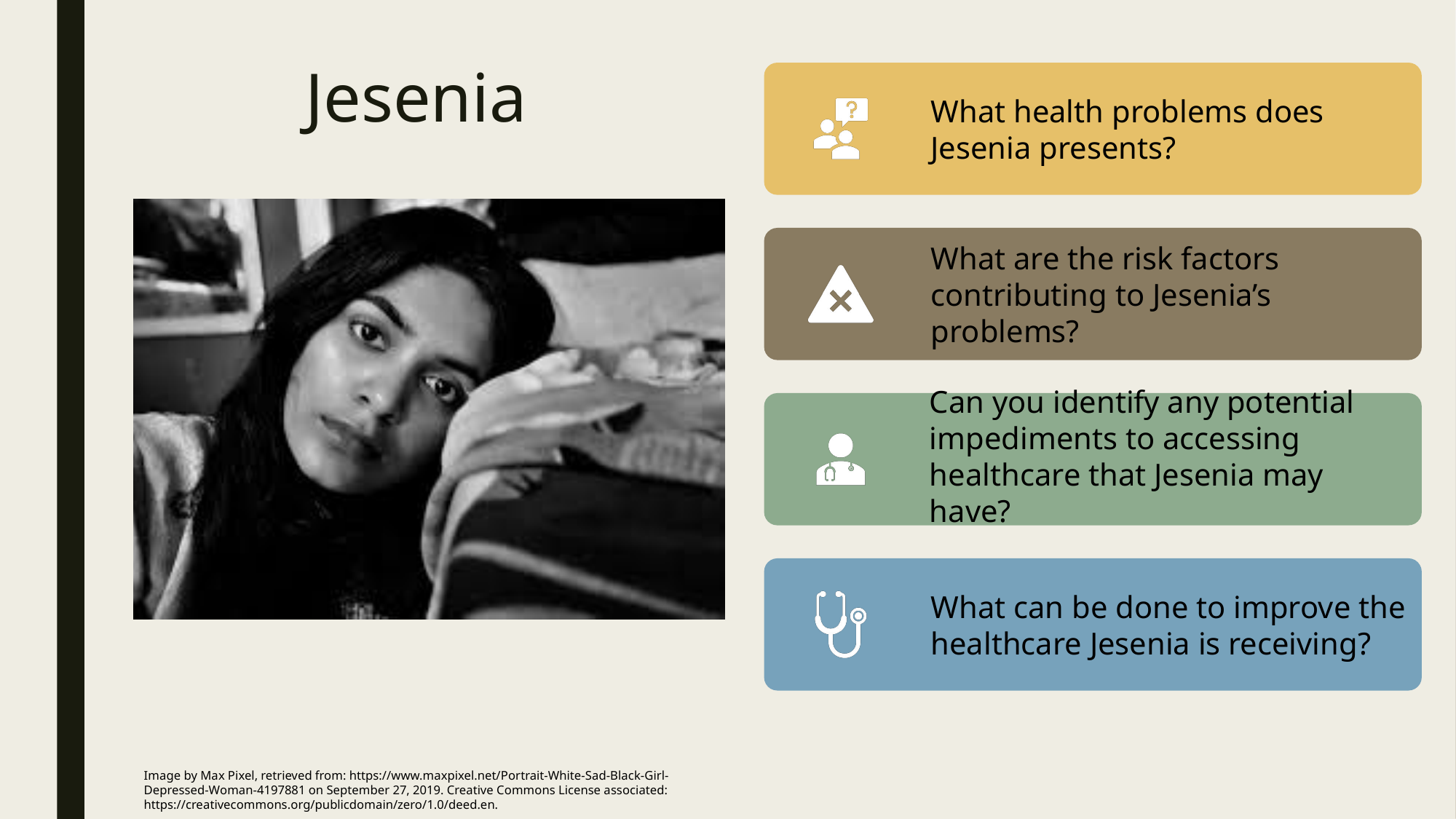

# Jesenia
Image by Max Pixel, retrieved from: https://www.maxpixel.net/Portrait-White-Sad-Black-Girl-Depressed-Woman-4197881 on September 27, 2019. Creative Commons License associated: https://creativecommons.org/publicdomain/zero/1.0/deed.en.

## Slide 42
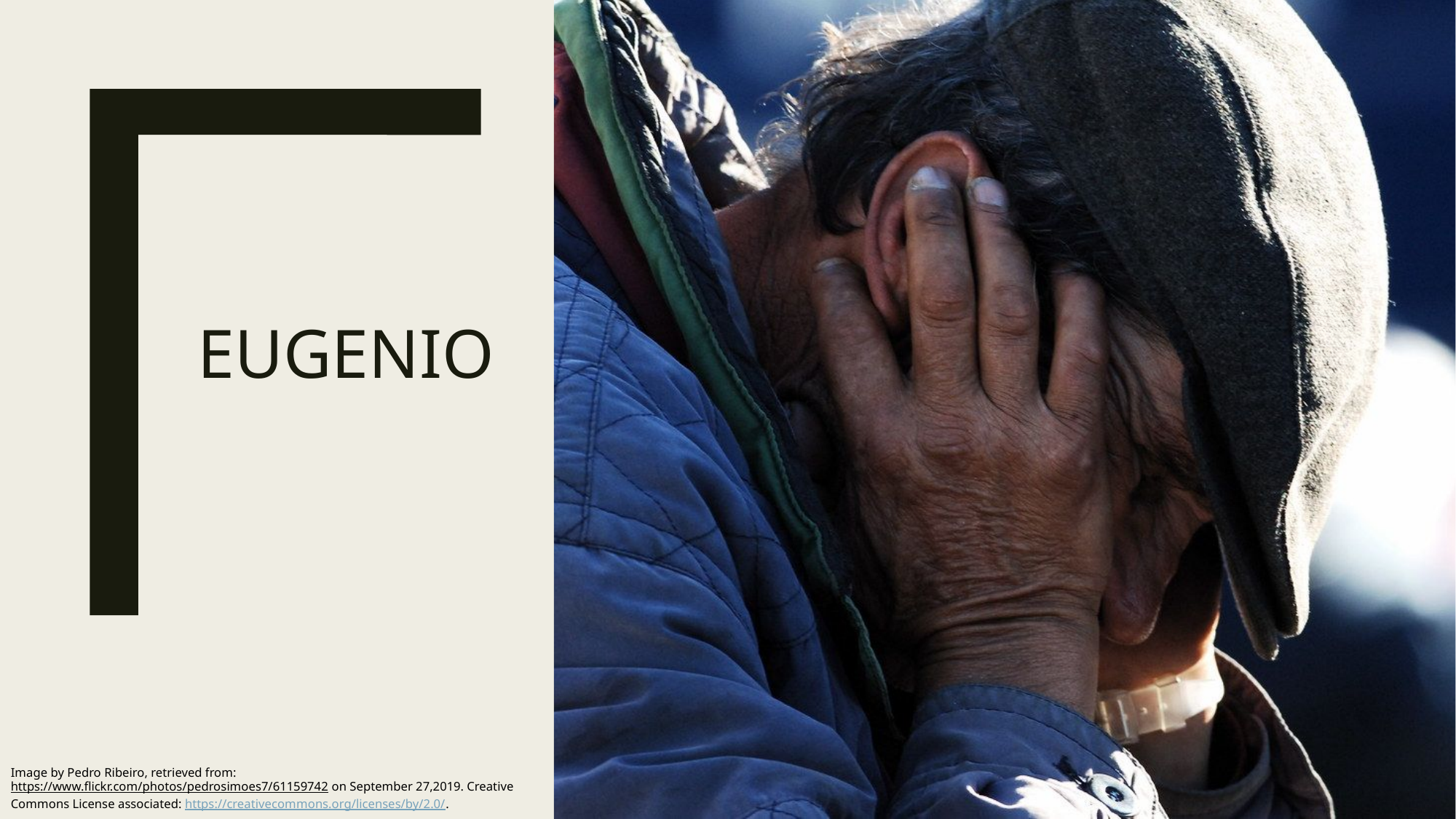

# Eugenio
Image by Pedro Ribeiro, retrieved from: https://www.flickr.com/photos/pedrosimoes7/61159742 on September 27,2019. Creative Commons License associated: https://creativecommons.org/licenses/by/2.0/.

## Slide 43
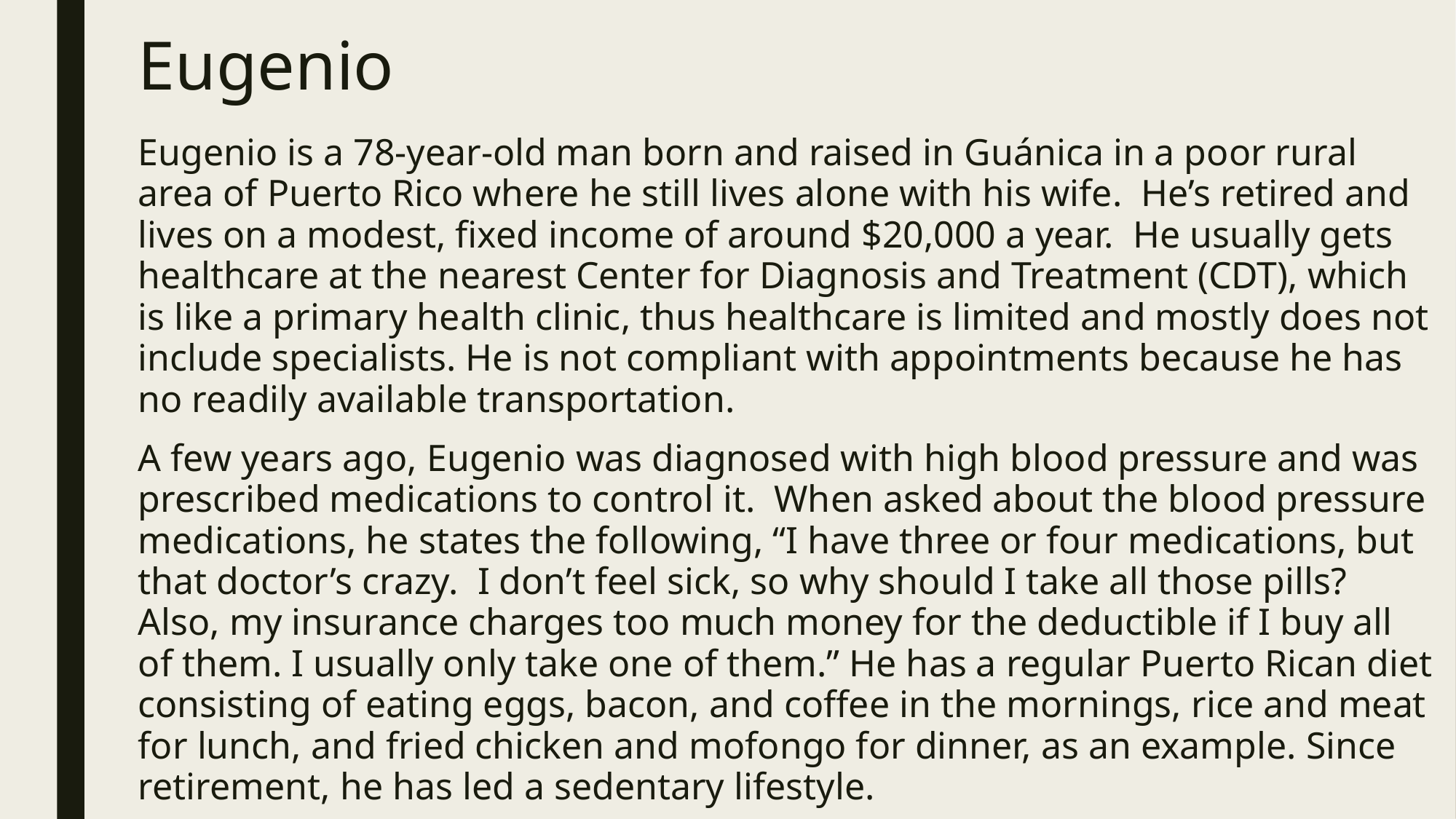

# Eugenio
Eugenio is a 78-year-old man born and raised in Guánica in a poor rural area of Puerto Rico where he still lives alone with his wife. He’s retired and lives on a modest, fixed income of around $20,000 a year. He usually gets healthcare at the nearest Center for Diagnosis and Treatment (CDT), which is like a primary health clinic, thus healthcare is limited and mostly does not include specialists. He is not compliant with appointments because he has no readily available transportation.
A few years ago, Eugenio was diagnosed with high blood pressure and was prescribed medications to control it. When asked about the blood pressure medications, he states the following, “I have three or four medications, but that doctor’s crazy. I don’t feel sick, so why should I take all those pills? Also, my insurance charges too much money for the deductible if I buy all of them. I usually only take one of them.” He has a regular Puerto Rican diet consisting of eating eggs, bacon, and coffee in the mornings, rice and meat for lunch, and fried chicken and mofongo for dinner, as an example. Since retirement, he has led a sedentary lifestyle.

## Slide 44
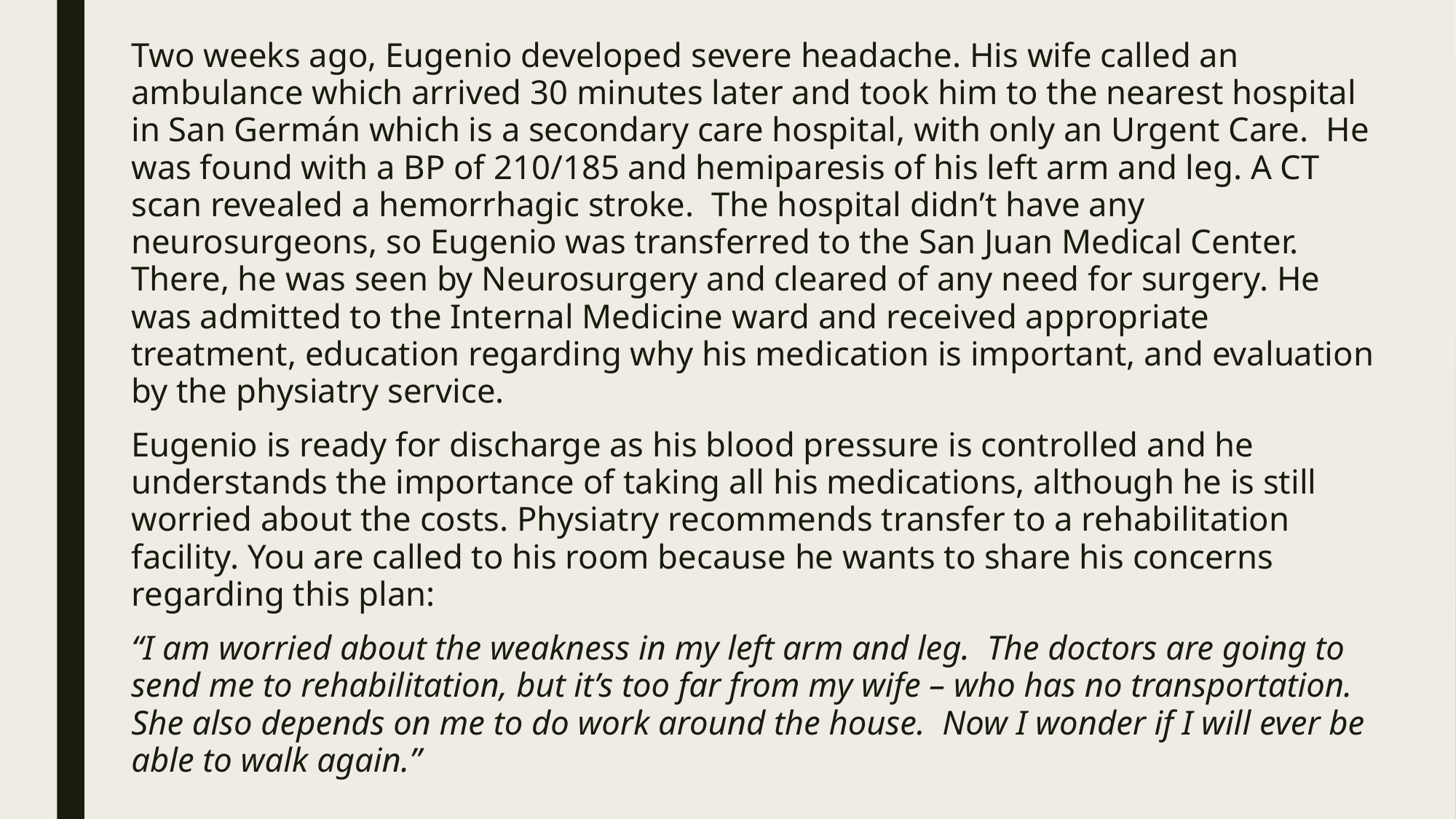

Two weeks ago, Eugenio developed severe headache. His wife called an ambulance which arrived 30 minutes later and took him to the nearest hospital in San Germán which is a secondary care hospital, with only an Urgent Care. He was found with a BP of 210/185 and hemiparesis of his left arm and leg. A CT scan revealed a hemorrhagic stroke. The hospital didn’t have any neurosurgeons, so Eugenio was transferred to the San Juan Medical Center. There, he was seen by Neurosurgery and cleared of any need for surgery. He was admitted to the Internal Medicine ward and received appropriate treatment, education regarding why his medication is important, and evaluation by the physiatry service.
Eugenio is ready for discharge as his blood pressure is controlled and he understands the importance of taking all his medications, although he is still worried about the costs. Physiatry recommends transfer to a rehabilitation facility. You are called to his room because he wants to share his concerns regarding this plan:
“I am worried about the weakness in my left arm and leg. The doctors are going to send me to rehabilitation, but it’s too far from my wife – who has no transportation. She also depends on me to do work around the house. Now I wonder if I will ever be able to walk again.”

## Slide 45
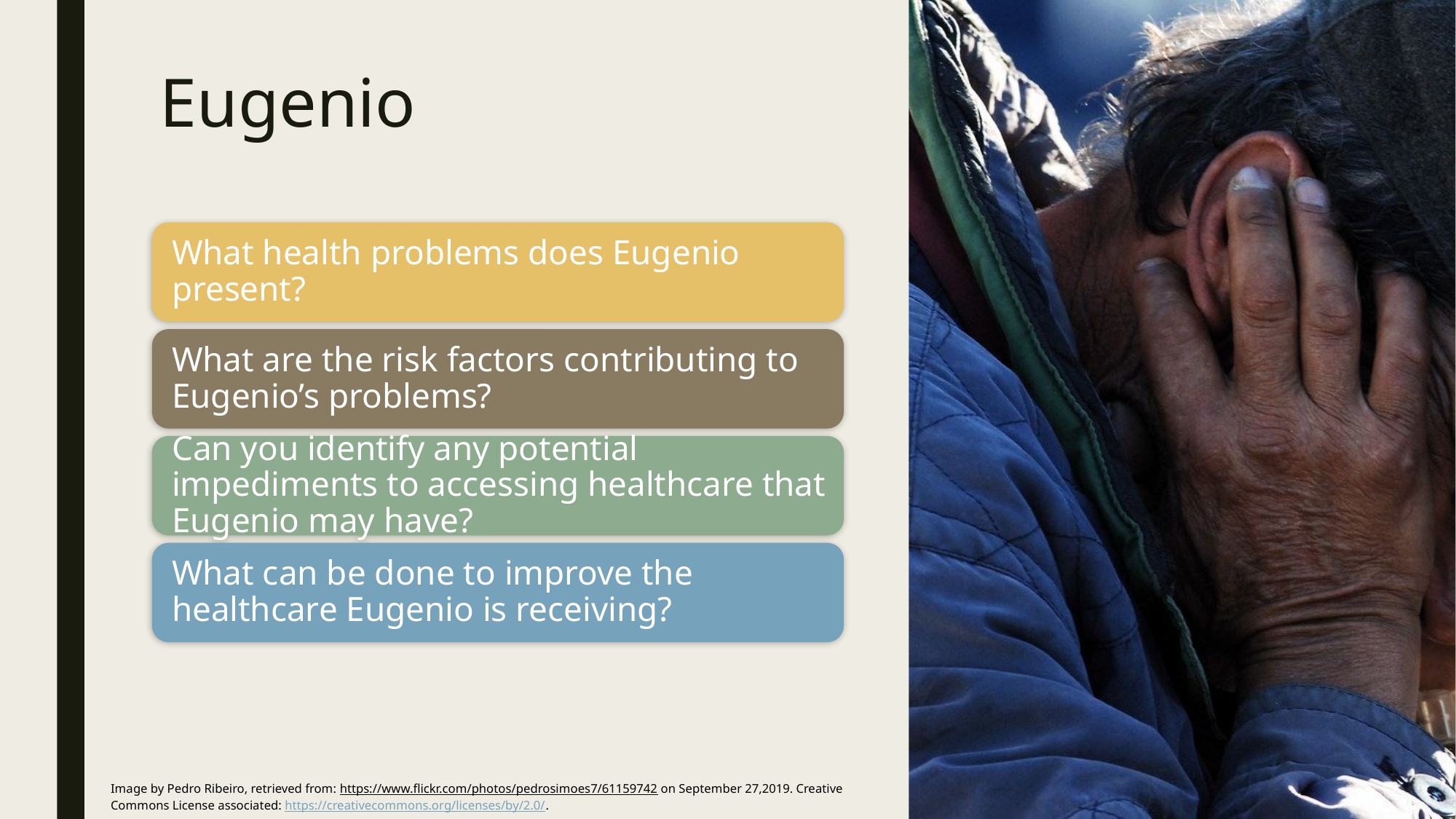

# Eugenio
Image by Pedro Ribeiro, retrieved from: https://www.flickr.com/photos/pedrosimoes7/61159742 on September 27,2019. Creative Commons License associated: https://creativecommons.org/licenses/by/2.0/.

## Slide 46
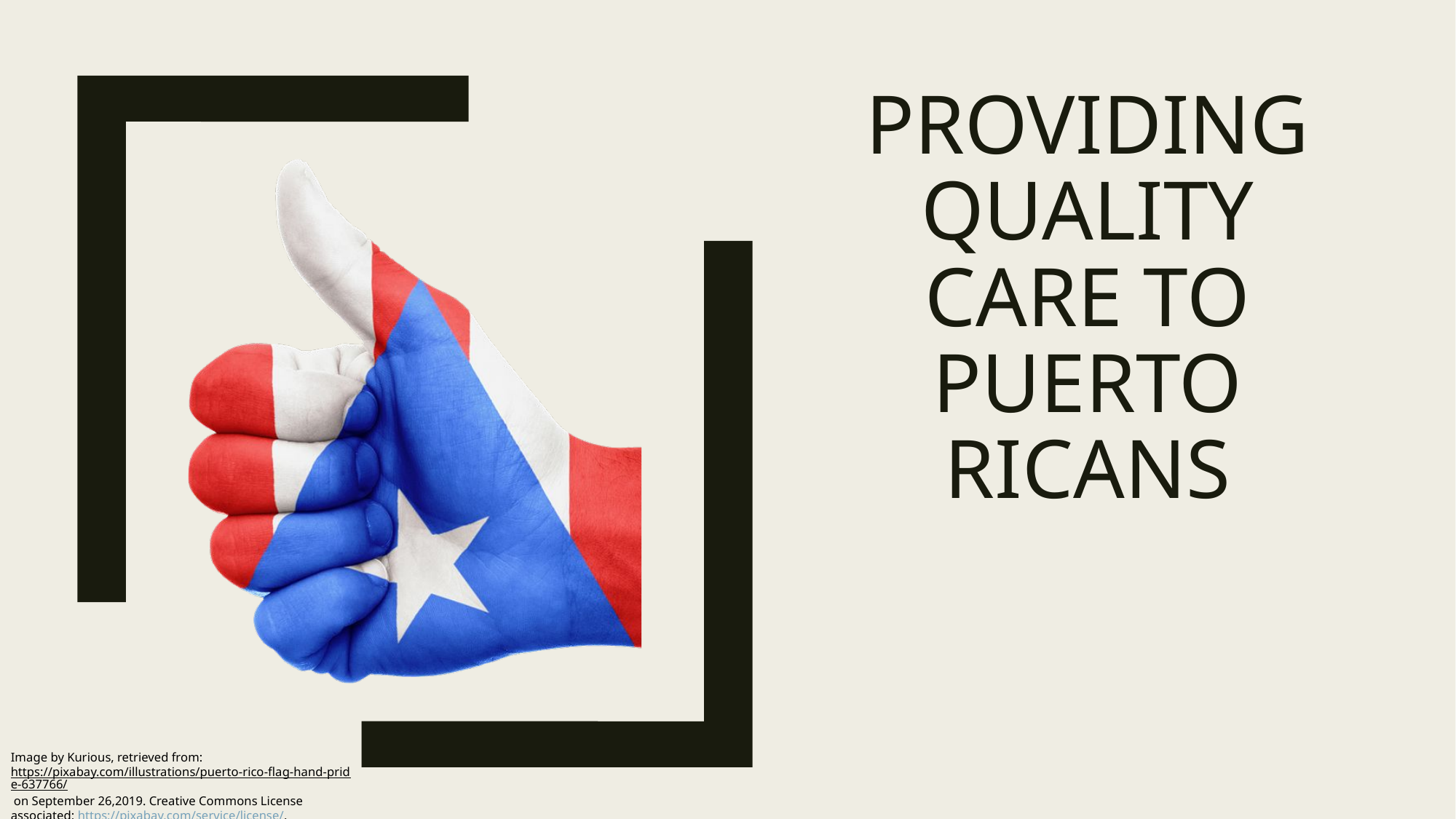

# Providing quality care to Puerto Ricans
Image by Kurious, retrieved from: https://pixabay.com/illustrations/puerto-rico-flag-hand-pride-637766/ on September 26,2019. Creative Commons License associated: https://pixabay.com/service/license/.

## Slide 47
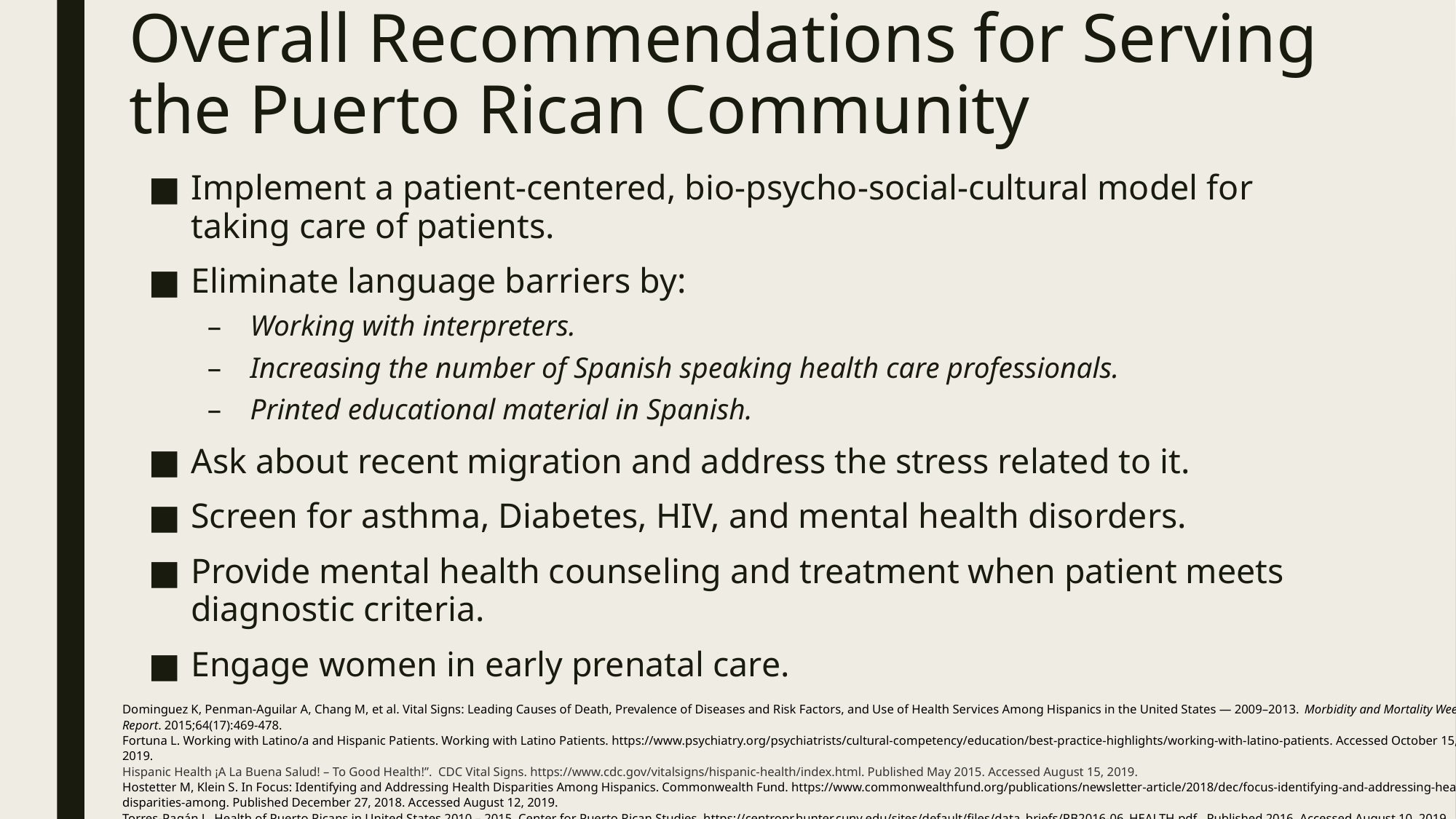

# Overall Recommendations for Serving the Puerto Rican Community
Implement a patient-centered, bio-psycho-social-cultural model for taking care of patients.
Eliminate language barriers by:
Working with interpreters.
Increasing the number of Spanish speaking health care professionals.
Printed educational material in Spanish.
Ask about recent migration and address the stress related to it.
Screen for asthma, Diabetes, HIV, and mental health disorders.
Provide mental health counseling and treatment when patient meets diagnostic criteria.
Engage women in early prenatal care.
Dominguez K, Penman-Aguilar A, Chang M, et al. Vital Signs: Leading Causes of Death, Prevalence of Diseases and Risk Factors, and Use of Health Services Among Hispanics in the United States — 2009–2013. Morbidity and Mortality Weekly Report. 2015;64(17):469-478.
Fortuna L. Working with Latino/a and Hispanic Patients. Working with Latino Patients. https://www.psychiatry.org/psychiatrists/cultural-competency/education/best-practice-highlights/working-with-latino-patients. Accessed October 15, 2019.
Hispanic Health ¡A La Buena Salud! – To Good Health!”. CDC Vital Signs. https://www.cdc.gov/vitalsigns/hispanic-health/index.html. Published May 2015. Accessed August 15, 2019.
Hostetter M, Klein S. In Focus: Identifying and Addressing Health Disparities Among Hispanics. Commonwealth Fund. https://www.commonwealthfund.org/publications/newsletter-article/2018/dec/focus-identifying-and-addressing-health-disparities-among. Published December 27, 2018. Accessed August 12, 2019.
Torres-Pagán L. Health of Puerto Ricans in United States 2010 – 2015. Center for Puerto Rican Studies. https://centropr.hunter.cuny.edu/sites/default/files/data_briefs/RB2016-06_HEALTH.pdf . Published 2016. Accessed August 10, 2019.

## Slide 48
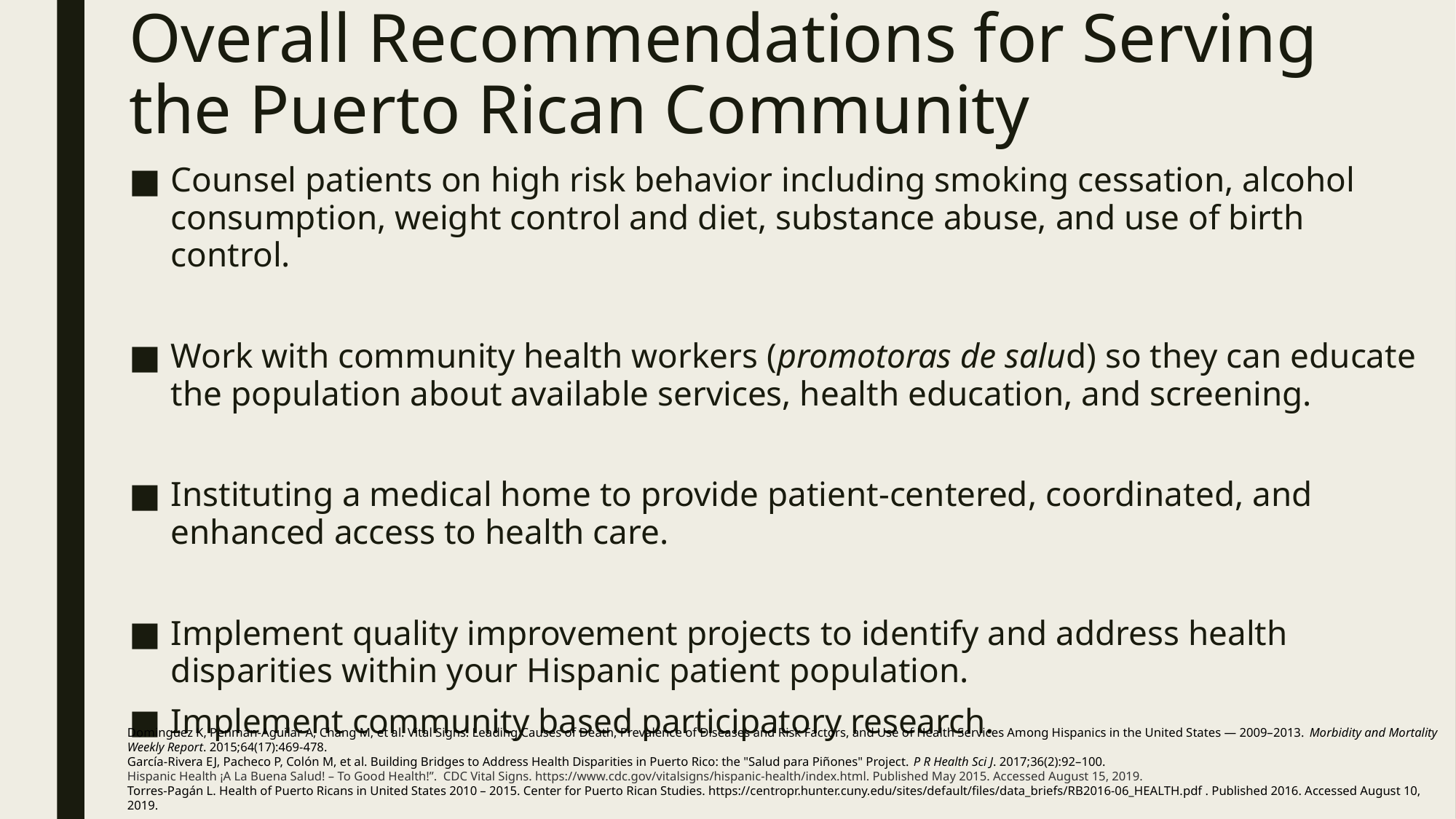

# Overall Recommendations for Serving the Puerto Rican Community
Counsel patients on high risk behavior including smoking cessation, alcohol consumption, weight control and diet, substance abuse, and use of birth control.
Work with community health workers (promotoras de salud) so they can educate the population about available services, health education, and screening.
Instituting a medical home to provide patient-centered, coordinated, and enhanced access to health care.
Implement quality improvement projects to identify and address health disparities within your Hispanic patient population.
Implement community based participatory research.
Dominguez K, Penman-Aguilar A, Chang M, et al. Vital Signs: Leading Causes of Death, Prevalence of Diseases and Risk Factors, and Use of Health Services Among Hispanics in the United States — 2009–2013. Morbidity and Mortality Weekly Report. 2015;64(17):469-478.
García-Rivera EJ, Pacheco P, Colón M, et al. Building Bridges to Address Health Disparities in Puerto Rico: the "Salud para Piñones" Project. P R Health Sci J. 2017;36(2):92–100.
Hispanic Health ¡A La Buena Salud! – To Good Health!”. CDC Vital Signs. https://www.cdc.gov/vitalsigns/hispanic-health/index.html. Published May 2015. Accessed August 15, 2019.
Torres-Pagán L. Health of Puerto Ricans in United States 2010 – 2015. Center for Puerto Rican Studies. https://centropr.hunter.cuny.edu/sites/default/files/data_briefs/RB2016-06_HEALTH.pdf . Published 2016. Accessed August 10, 2019.

## Slide 49
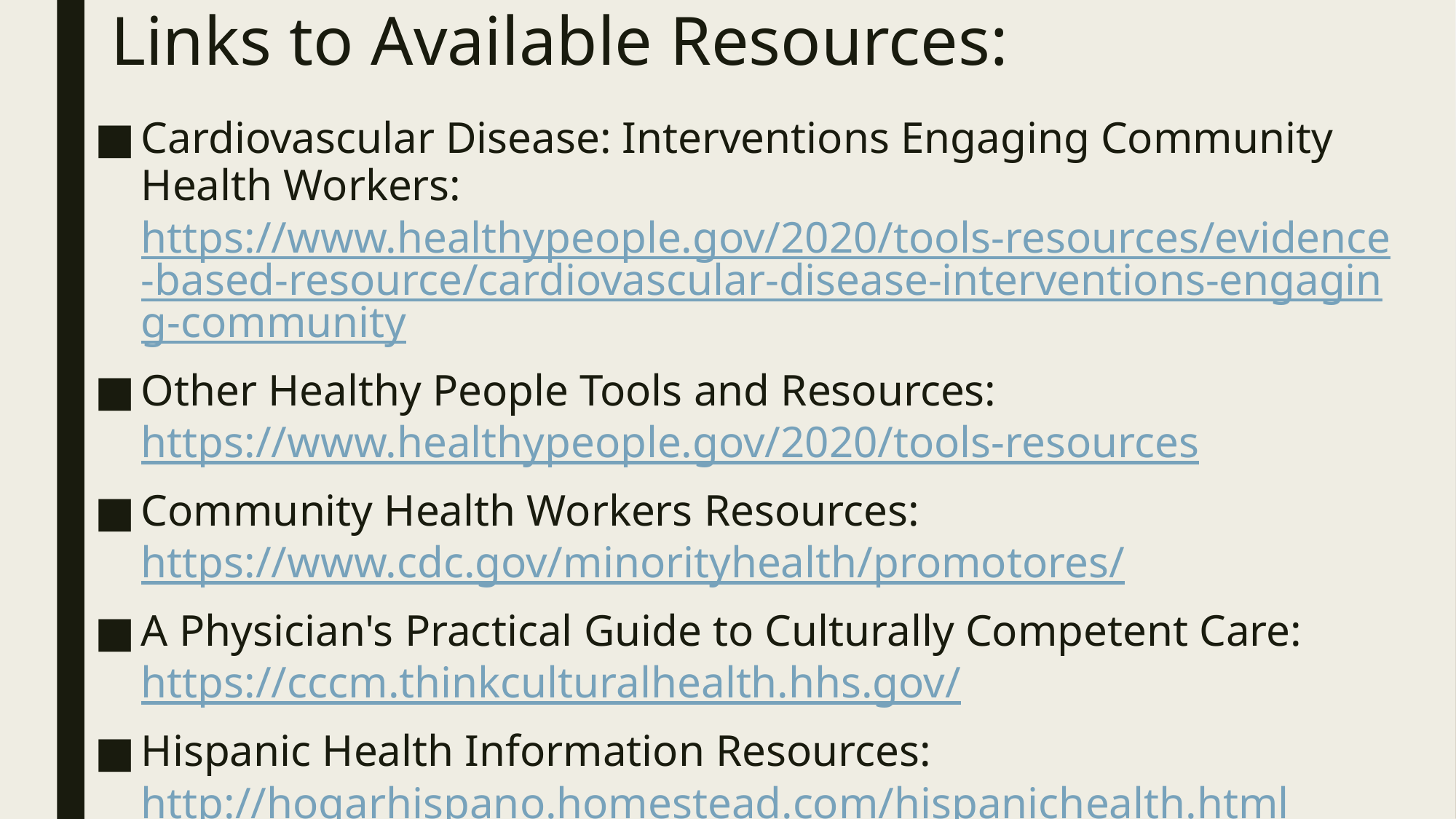

# Links to Available Resources:
Cardiovascular Disease: Interventions Engaging Community Health Workers: https://www.healthypeople.gov/2020/tools-resources/evidence-based-resource/cardiovascular-disease-interventions-engaging-community
Other Healthy People Tools and Resources: https://www.healthypeople.gov/2020/tools-resources
Community Health Workers Resources: https://www.cdc.gov/minorityhealth/promotores/
A Physician's Practical Guide to Culturally Competent Care: https://cccm.thinkculturalhealth.hhs.gov/
Hispanic Health Information Resources: http://hogarhispano.homestead.com/hispanichealth.html

## Slide 50
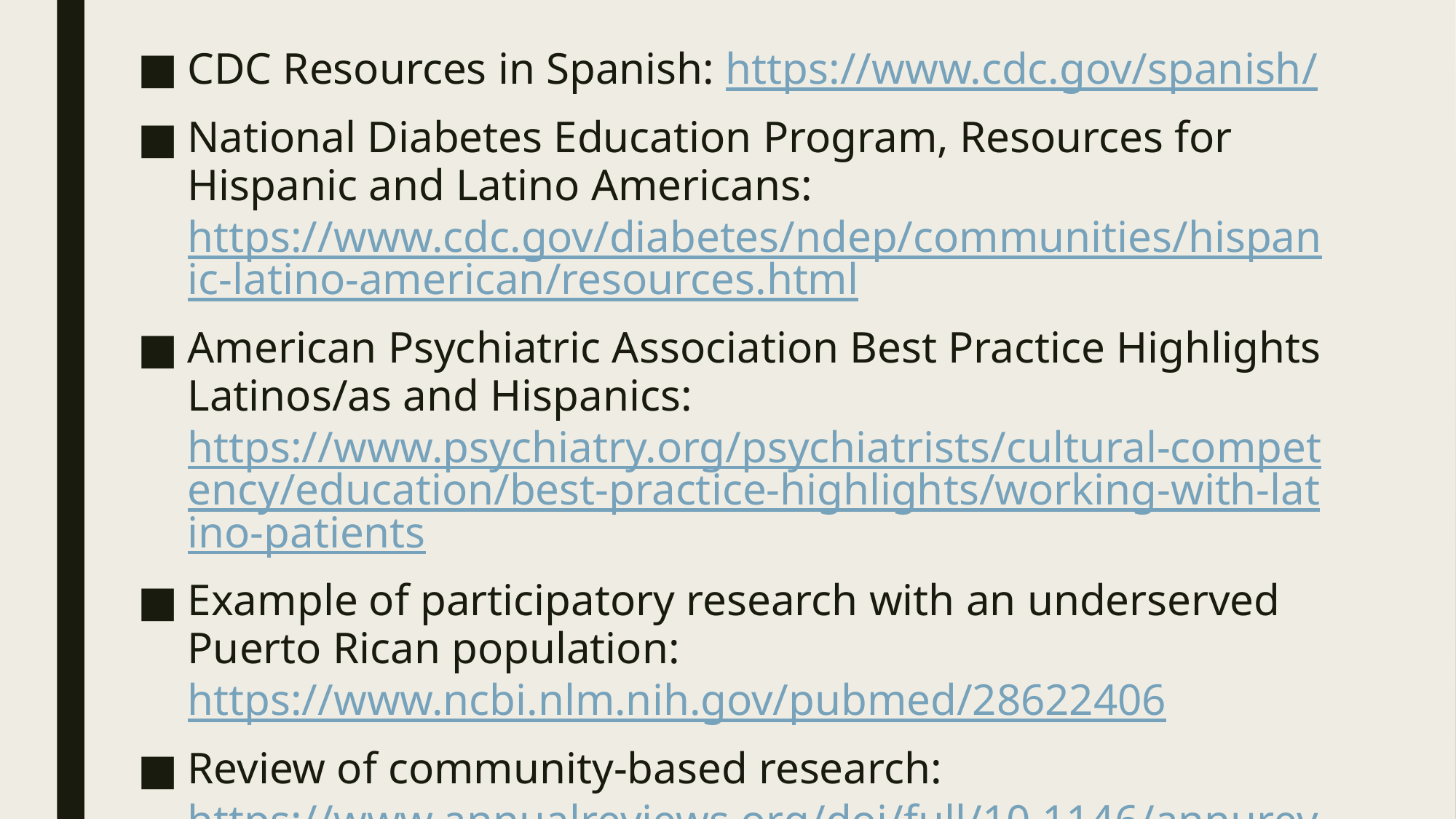

CDC Resources in Spanish: https://www.cdc.gov/spanish/
National Diabetes Education Program, Resources for Hispanic and Latino Americans: https://www.cdc.gov/diabetes/ndep/communities/hispanic-latino-american/resources.html
American Psychiatric Association Best Practice Highlights Latinos/as and Hispanics: https://www.psychiatry.org/psychiatrists/cultural-competency/education/best-practice-highlights/working-with-latino-patients
Example of participatory research with an underserved Puerto Rican population: https://www.ncbi.nlm.nih.gov/pubmed/28622406
Review of community-based research: https://www.annualreviews.org/doi/full/10.1146/annurev.publhealth.19.1.173

## Slide 51
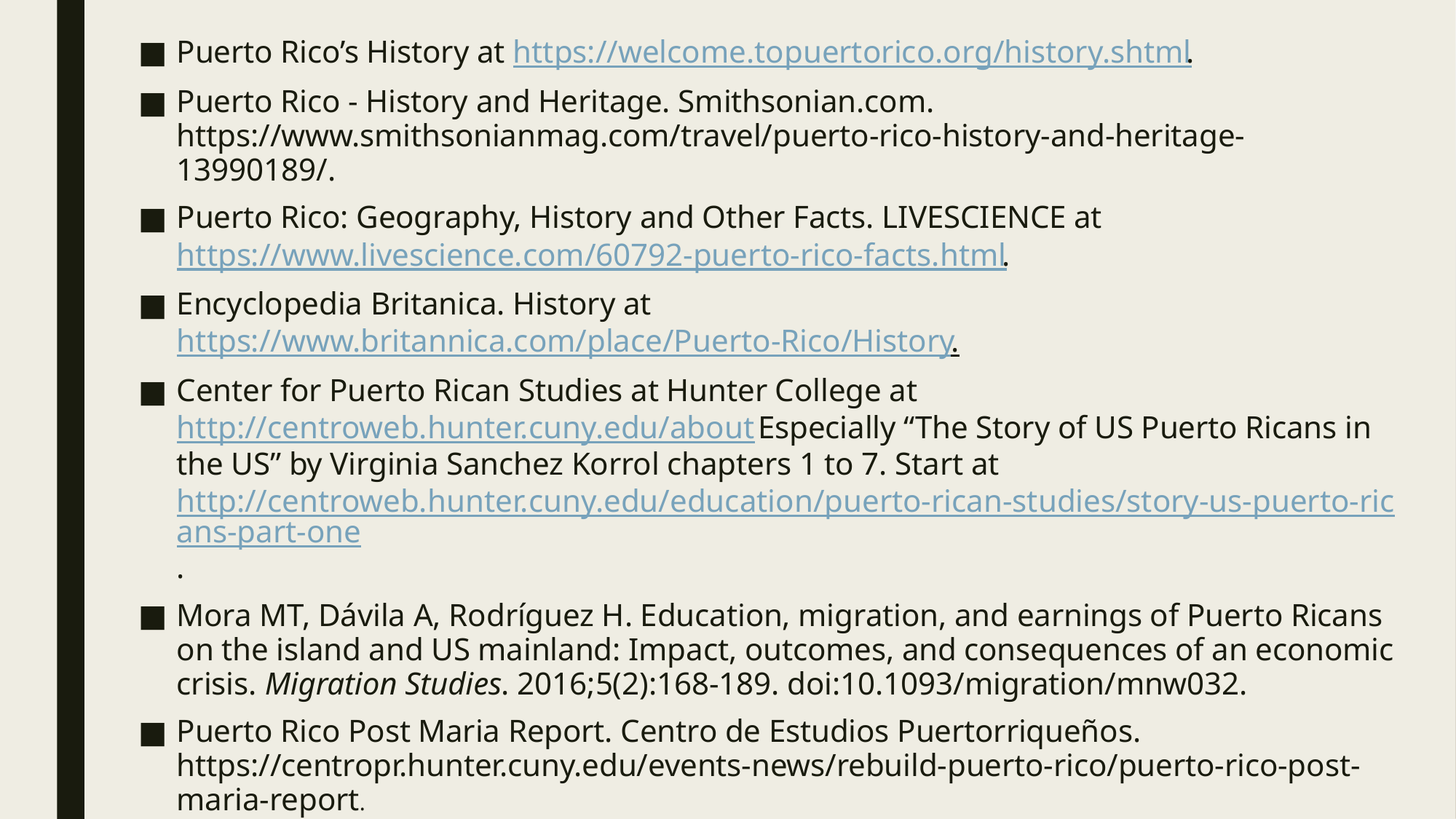

Puerto Rico’s History at https://welcome.topuertorico.org/history.shtml.
Puerto Rico - History and Heritage. Smithsonian.com. https://www.smithsonianmag.com/travel/puerto-rico-history-and-heritage-13990189/.
Puerto Rico: Geography, History and Other Facts. LIVESCIENCE at https://www.livescience.com/60792-puerto-rico-facts.html.
Encyclopedia Britanica. History at https://www.britannica.com/place/Puerto-Rico/History.
Center for Puerto Rican Studies at Hunter College at http://centroweb.hunter.cuny.edu/about Especially “The Story of US Puerto Ricans in the US” by Virginia Sanchez Korrol chapters 1 to 7. Start at http://centroweb.hunter.cuny.edu/education/puerto-rican-studies/story-us-puerto-ricans-part-one.
Mora MT, Dávila A, Rodríguez H. Education, migration, and earnings of Puerto Ricans on the island and US mainland: Impact, outcomes, and consequences of an economic crisis. Migration Studies. 2016;5(2):168-189. doi:10.1093/migration/mnw032.
Puerto Rico Post Maria Report. Centro de Estudios Puertorriqueños. https://centropr.hunter.cuny.edu/events-news/rebuild-puerto-rico/puerto-rico-post-maria-report.

## Slide 52
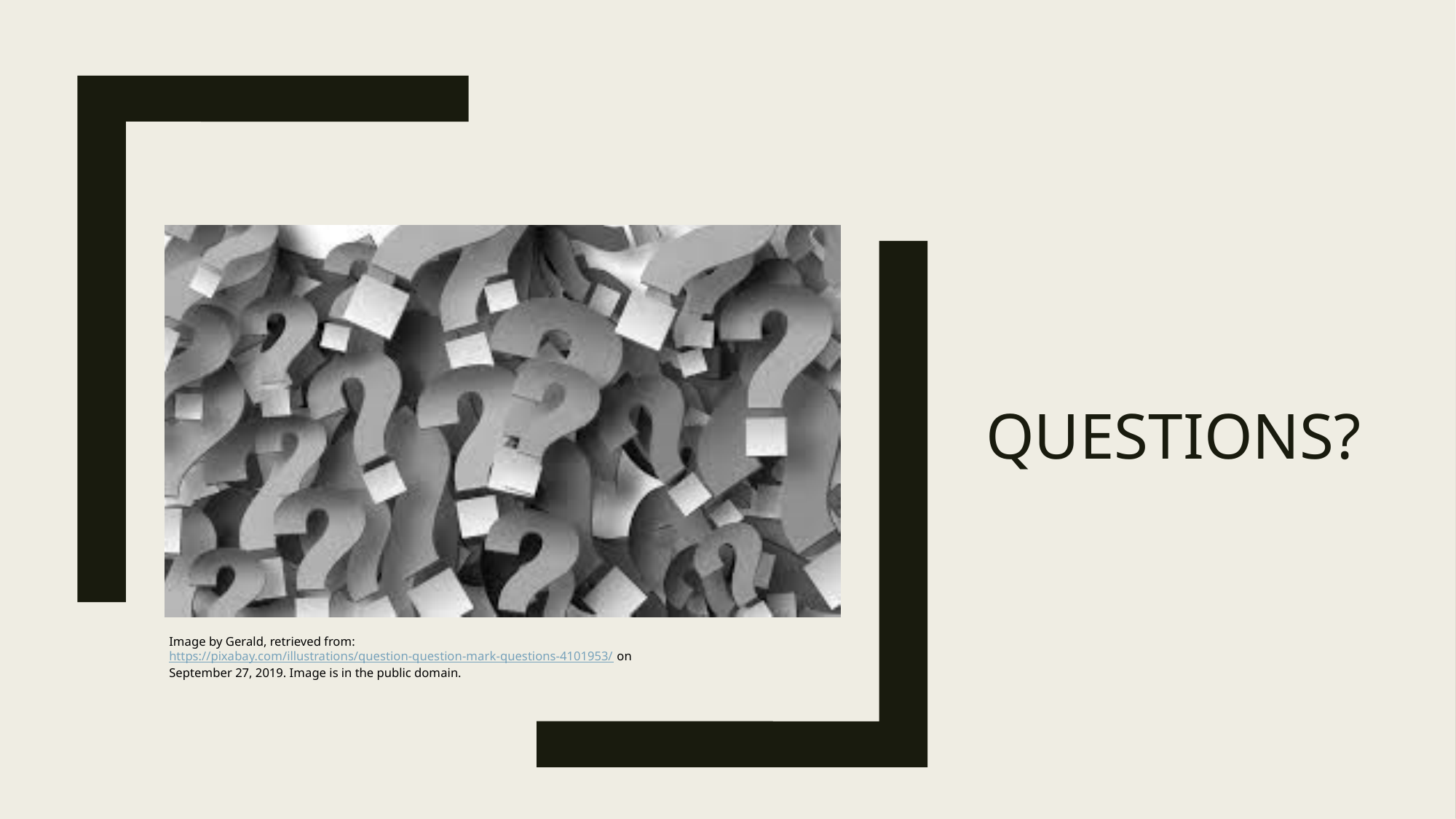

# Questions?
Image by Gerald, retrieved from: https://pixabay.com/illustrations/question-question-mark-questions-4101953/ on September 27, 2019. Image is in the public domain.
