# Supplementary material for: Taking Care of the Puerto Rican Patient: Historical Perspectives, Health Status, and Health Care Access
Source: MedEdPORTAL. 2020 Oct 7;16:10984. doi: 10.15766/mep_2374-8265.10984 (PMC7549386; doi:10.15766/mep_2374-8265.10984)
Supplement: Supplementary file 1 — Taking Care of the Puerto Rican Patient.pptxFacilitator Guide.docxEvaluation Forms.docx [file mep_2374-8265.10984-s001.zip › B. Facilitator Guide.docx]

**Taking Care of the Puerto Rican Patient:**

**Historical Perspectives, Health Status, and Health Care Access**

**Facilitator Instructional Guide**

**Overall Goals**

The goals of this module are to help health professionals, trainees, and pre – health students understand and analyze health disparities, access to care, and how identity influences the health status of the Puerto Rican population on the mainland and on the island with the purpose of improving their care. This module may be implemented by individuals of any background.

**Workshop Objectives**

- Describe the history of Puerto Rican identity within the United States.
- Compare and contrast health issues and disparities of Puerto Ricans residing on the island and on the mainland.
- Explain how at least one state or federal policy has impacted the health outcomes of Puerto Ricans on the mainland and/or the island.
- Identify at least two health care access problems faced by Puerto Ricans on the mainland and/or the island.

**Workshop Handouts and Materials**

- Evaluation Forms
- Computer set up and connection to projector

**Suggested Agenda and Timeline**

- Pre-workshop evaluation: 2-3 minutes
- Slides 1- 7: Introduction, Objectives, Agenda, History, Identity Formation- 5 minutes
- Slides 8-29: Migration, Health Care Status and Health Statistics of Puerto Ricans on the island and mainland US, and Puerto Ricans in NYC example: 20 minutes
- Slides 30-31: Case discussion and reflection exercises- 20 minutes
- Slide 45-50: Health Care Recommendations, Questions and Answers: 10 minutes
- Post-workshop evaluation: 2-3 minutes

**For Facilitators that allot more time for the workshop, alternative information may be added to the presentation when discussing slides and/or case discussions may be in a small group format. Alternatives are marked with an asterisk (*).*

**Suggested Reading Material and Resources for Facilitators in Preparation for Workshop**

- Puerto Rico’s History at <https://welcome.topuertorico.org/history.shtml>.
- Puerto Rico - History and Heritage. Smithsonian.com. https://www.smithsonianmag.com/travel/puerto-rico-history-and-heritage-13990189/.
- Puerto Rico: Geography, History and Other Facts. LIVESCIENCE at <https://www.livescience.com/60792-puerto-rico-facts.html>.
- Encyclopedia Britanica. History at <https://www.britannica.com/place/Puerto-Rico/History>,
- Center for Puerto Rican Studies at Hunter College at <http://centroweb.hunter.cuny.edu/about> Especially “The Story of US Puerto Ricans in the US” by Virginia Sanchez Korrol chapters 1 to 7. Start at <http://centroweb.hunter.cuny.edu/education/puerto-rican-studies/story-us-puerto-ricans-part-one>.
- Mora MT, Dávila A, Rodríguez H. Education, migration, and earnings of Puerto Ricans on the island and US mainland: Impact, outcomes, and consequences of an economic crisis. *Migration Studies*. 2016;5(2):168-189. doi:10.1093/migration/mnw032.
- Puerto Rico Post Maria Report. Centro de Estudios Puertorriqueños. https://centropr.hunter.cuny.edu/events-news/rebuild-puerto-rico/puerto-rico-post-maria-report.

**Slide Instructions**

This module should be modified by updating statistics and information from a literature review. Facilitators may wish to include specific examples of the state/city they will be presenting as a contextualized example in substitution of the NYC example slides. Implementation may be adapted to include only mainland Puerto Ricans, island Puerto Ricans, or both. Facilitators may also choose to divide the large group into small groups for the case discussions.

The number of facilitators will depend on the option for case discussions chosen. One facilitator is enough for the workshop as it was implemented. We suggest three facilitators, one for each case, if the chosen option is to discuss cases in small groups prior to the large group debriefing.

**Slide 1**: Title Slide

Add names and background information of facilitators to slide.

The facilitators should introduce themselves to the audience and discuss their roles in their respective institutions. Facilitators may choose to mention they are following the content and guidelines of this peer-reviewed module.

Facilitators will specify that, throughout the workshop, the term “island” is referring to the Island of Puerto Rico and the term “mainland” is referring to the Continental US.

**Slide 2:**

*Migration from PR to the mainland has been going on for centuries, and Puerto Ricans in the mainland make up the second largest Hispanic population, with the first being Mexicans*.^1^ *Thus, health care providers need to know how to improve their healthcare.*

*With this in mind, at the end of the workshop you all should be able to:*

- *Describe the history of Puerto Rican identity within the United States.*
- *Compare and contrast health issues and disparities of Puerto Ricans residing on the island and on the mainland.*
- *Explain how, at least, one state or federal policy has impacted the health outcomes of Puerto Ricans on the mainland and/or the island. AND*
- *Identify, at least, two health care access problems faced by Puerto Ricans on the mainland and/or the island.*

**Slide 3:**

Please read the agenda:

- *Introduce Puerto Rican history and identity formation.*
- *Discuss health statistics and healthcare of Puerto Ricans in the mainland and in the island.*
- *Discuss some cases and reflect on them, and*
- *Finish with some recommendations to health care providers for improving the health care of Puerto Ricans.*

**Slide 4:**

Transition slide.

**Slide 5:**^1-4^

The facilitator should discuss important historical points for each of the dates/titles in bold. The following is the recommended information to provide:

**Pre-1493- Taino settlement; Borikén:***The original inhabitants of Puerto Rico where the Taino Indians, who were a Subgroup of Arawak Amerindian Indians which migrated from what is now Venezuela and Borikén was the Taino name for Puerto Rico.*

- *This is the reason that Puerto Rico, a name given to the island by the Spaniards, is also called Borínquen by Puerto Ricans and they self-identify as Puerto Ricans, Borinqueños or Borincanos interchangeably****.***

**1493-1898- Spanish colonization; African Slave Trade; Loss of Taino civilization:**

- *In 1493 Christopher Columbus arrived to Borikén and Tainos were used as slaves for mining gold and building forts, they quickly decimated by diseases brought in by the Spaniards, such as Smallpox, and by violence.*
- *Africans from Spain and West Africa were brought in as slaves beginning around 1517.*

**Slide 6:** ^1-4^

**1898- 1901- Spanish American War; Treaty of Paris; Foraker Act; Insular Cases:**

- *In 1898, the Treaty of Paris ended the Spanish American War and established Cuba, Guam, the Philippines, and Puerto Rico as American territories. In the next few years, the US struggled to define its colonies.*
- *In 1900, the US Congress passed the Foraker Act and established a limited civil government in Puerto Rico.*
- *Puerto Ricans thought they had the same constitutional rights as US Citizens but in 1901, the US Supreme Court decisions in* ***the Insular Cases*** *declared Puerto Rico to be separate from and unequal to the states.*
- *The Insular cases’ decision set the precedent for the unequal federal funding for Medicare and Medicaid in Puerto Rico that are still prevalent today. Even now, Puerto Ricans pay the same social security and Medicaid that other Americans pay but get less benefits and doctors get paid less than those in the states.*
- *In 1917 Congress Approved the Jones Act and American citizenship was given to those who wanted it and all those born in Puerto Rico.*

**Alternative addition: Prior to beginning the discussion of the Treaty of Paris, discuss the Spanish American War: The Spanish American War was a conflict between the United States and Spain, which began when Cuba fought for their independence from Spain. The US decided to support Cuba in its efforts of independence and because of this, Spain declared war on the US and vice versa. The US won the war, Cuba was granted its independence, and at the Treaty of Paris was signed.*

**1946 – 1979- Commonwealth Status:**

- *In 1952 Commonwealth status was granted to Puerto Rico by Congress, in Spanish it is called El Estado Libre Asociado de Puerto Rico. As a Commonwealth, PR is a semi-autonomous territory under Congressional jurisdiction.*

**Alternative addition: Of note is that Puerto Ricans have always been divided into three positions: those that want to be a state of the US, those that want to be an independent country, and those that want to maintain the status as a semi-autonomous territory.*

**Slide 7:**

*Puerto Rican culture is a mixture of Taino, Spanish, African, and American cultures.*

*Cultural influences on the health of Puerto Ricans include their food preference, known locally as “cocina criolla” or Creole cooking.*

*For example:*

*The Tainos brought corn, yucca, and other starchy root vegetables to Puerto Rico, the invention of barbecue cooking, and the pilón (mortar and pestle) used to mash the foods.*

*The Africans plantains and gandules which are staples in the Puerto Rican diet; used to make mofongo, tostones (pictured) and arroz con gandules (rice with pigeon peas).*

*The Spanish introduced pork, beef, rice, wheat, and olive oil. In fact, the first step in Spanish colonization was often dropping off pigs and cattle.*

*While the Americans introduced pizza, hamburgers, French fries, and most of the fast-food establishments on the island.*

*Many of these high starch and high fat foods are fried, which we know increases the risk for developing health issues like obesity, diabetes, hypertension, and heart disease. Thus, we see how the Puerto Rican diet, albeit delicious, contributes to some of the major health issues we’ll describe later on.*

**Slide 8:**

*Another component of health, is genetic predisposition and it is now known that Puerto Rican DNA is a mixture of : 12% Native American; 65% West Eurasian Including Mediterranean, Northern European and/or Middle Eastern (Spain was occupied by a Middle Eastern caliphate for centuries), and 20% Sub-Saharan African.*

**Slide 9:**

Transition slide.

**Slide 10:**

*The population of PR has decreased from 3.7 Million in 2010 to around 3.1 million estimated for 2018, most of this decrease is due to migration in the last decade. Therefore, it is important that health care providers be knowledgeable of the most common causes of morbidity and mortality in Puerto Ricans from the island.*

*Risk factors for Puerto Ricans in the island begin with their living conditions. For example, as of 2017 92% of municipios (counties) in PR were classified as medically underserved areas and the top 10 poorest counties in the US were from PR.*

*In 2018, the US Census Bureau reported that 44.4% of the population lived in poverty.*

**Slide 11:**

*In 2017 three of the top 5 causes of mortality- Ischemic Heart Disease, Diabetes, and Stroke were the same, or directly related to the three most prevalent chronic conditions, high blood pressure, diabetes, and hyperlipidemia.*

**Slide 12:**

*The top 5 Risk factors for death and disability are mostly predictable based on chronic conditions and death causes: High Fasting Plasma Glucose, High Body Mass Index, Dietary Risks, High Blood Pressure, and Alcohol Abuse.*

*Almost everything on this slide can be associated with the diet we previously discussed. The rice and starchy vegetables that sustained the poor have now become harmful in times where food is more plentiful.*

**Slide 13:**

*Diabetes is a big problem for Puerto Ricans. Puerto Ricans in the island have 50% higher prevalence and three times the death rate from diabetes than the rest of the US. As compared to Mainland Puerto Ricans, death rates from Diabetes are higher for those living in the Island.*

**Slide 14:**

*Other conditions to keep in mind include viruses acquired through Aedes Aegyptus which is endemic in PR such as Dengue Fever, Zika, and Chikungunya, and Health issues associated with natural disasters including: Poor access to health care and treatment for chronic diseases, poor hygiene and increased risk of infectious diseases, and an increase in Mental health issues.*

**Slide 15:***For example,*

*After Hurricane Maria hospital access was limited and most of the dialysis centers lost their power; there was an increase in Leptospirosis infection; and an increase in suicides, anxiety and depression.*

*In a study done by Scaramutti and colleagues, they compared rates of PTSD of Puerto Ricans in the island and those relocated to Florida after Hurricane Maria and found that the incidence of PTSD was 43.6% for Puerto Ricans in the island vs. 67.7% for those who relocated to Florida.*

*In addition, lack of English proficiency increased mental distress in those migrating immediately after the Hurricane.*

**Alternative Addition: Prior to discussing the effects of Hurricane Maria in those migrating to the US, discuss the lack of preparation and inadequate response of the local government including minimizing deaths associated with the Hurricane. While the government of PR stated there were only 64 deaths attributable to the Hurricane, studies such as the one done by Kishore and colleges, estimates deaths to be 70 times higher than the 64.^5^ Studies also indicate that the Federal Government response was inadequate with far less allocation of resources and money directed to Puerto Rico than to areas affected by Hurricanes Harvey and Irma, although Hurricane Maria caused much more damage and deaths.^6^*

**Slide 16:**

Transition slide.

*A critical part of the history of Puerto Ricans in the mainland has been the migration trends*

**Slide 17:**^7-9^

The facilitator should discuss important historical points for each of the dates/titles in bold. The following is the recommended information to provide:

*Puerto Ricans have been migrating to the mainland since the last half of the 19^th^ century.*

**1921- 1939- Lose Sugar & Coffee; Great Depression:**

- *In the Early 1900’s migration spiked due to the US Great Depression.*
- *During this time conditions in Puerto Rico were deplorable with most of the population in poverty. Especially after another Hurricane destroyed sugar and coffee plantations.*
- *The U.S. believed that the issue was overpopulation. The Government-sponsored emigration and birth control; specifically, government sponsored sterilization.*

**1946- 1979- Operation Bootstrap:**

- *The Great Migration of the 1940’s to -1960’s was directly linked to Operation Bootstrap (Operación Manos a la Obra) – a Project approved in 1947 by congress to improve the financial situation of PR, by industrialization and tax exemption. US companies moved to the island creating jobs. There were not enough jobs in the island couples with the loss of agricultural jobs caused by hurricanes and industrialization, pushed poor, unskilled laborers to the mainland*
- *Migrants went to factories in the Northeast (New York, Philadelphia, & Boston) and Midwest (Chicago).*

**1980’s- US Recession; Adjustments to Minimum Wage:**

- *Another migration spike occurred in the Mid 1980’s concomitant to the U.S. recession due to high oil prices, which applied to PR.*
- *In addition, the Federal Government increased the jobs eligible for Federal Minimum Wage which was previously lower in the island than in the mainland to prevent economic collapse. Because of this increase, local companies closed, and jobs were lost.*

**Slide 18:**^9, 11-14^

The facilitator should discuss important historical points for each of the dates/titles in bold. The following is the recommended information to provide:

**2006 – 2016- Bootstrap Tax Breaks End; Great Recession; PR Financial Crisis:**

- *Since 2006 PR has been in the middle of a Great Recession and a Financial Crisis. During this time, Corporate tax exemptions ended which caused massive exodus of companies and factories and thus, loss of jobs.*
- *In addition to this, PR has been in a financial crisis which has increased the cost of living. As a consequence, more than 500,000 Puerto Ricans migrated to the mainland from 2006-2017.*

**2017- Hurricane María; Law 14 of 2017**

- *The last migration spike happened after Hurricane Maria hit the island on September 2017. It is estimated that, during the first six months after the hurricane, more than 130,000 Puerto Ricans relocated to the mainland as a result of the natural disaster. The Government already in crisis did not have the resources to adequately deal with the devastation caused by the hurricane including the ability to provide access to healthcare.*
- *It is well known that Doctors migrate to the mainland at higher rates than the rest of the population causing a need for doctors. In response to this, the PR congress passed Law 14 of 2017 which is intended to prevent the exodus of medical personnel by capping the state income tax rate at 4% for physicians, podiatrists, and dentists with the requirement that they provide 180 hours of community service per year to vulnerable populations for at least 15 years.*

**Slide 19:**

*How many Puerto Ricans live on the mainland? Prior to Hurricane Maria, which was on 2017, around 5,5 million Puerto Ricans lived on the mainland, most of whom were born in the United States.^15^ It is estimated that between 2017 and 2019 more than 400,000 Puerto Ricans will have migrated from PR to the mainland which would mean that there will be twice as many Puerto Ricans on the mainland than on the island.*

**Slide 20:**

*Where in the mainland they live? Traditional enclaves have always included New York, Philadelphia, Chicago, Connecticut, and Massachusetts. In the last few years, Puerto Ricans migrating and those being born on the mainland are choosing to live in new enclaves in Florida, Texas, and the Southeast due to Loss of job markets in the traditional areas that attracted migrants. After Hurricane Maria most relocated to Florida and the southeast.*

**Slide 21:**

Transition slide.

*What is the health status of Puerto Ricans on the Mainland?*

**Slide 22:**

*In a survey done in 2015 by Medicare, Puerto Ricans from the Mainland reported higher rates of having access to care as defined by knowing who and where to look for care, as compared to those in the island and other Hispanic patients, but were less satisfied than both groups with the ease of getting from their home to their doctors. This is also a problem of access to care.*

**Slide 23:**

*Data from the National Health Interview Survey from 2010–2014 demonstrated, that as compared to other Hispanic and Non-Hispanic adults:*

*Puerto Rican adults in the mainland consistently report poorer health status; were more likely to have had multiple chronic conditions; when asked if they could go out shopping, the movies, sports events, visit friends, attend clubs and meetings or going to parties, Mainland Puerto Rican adults were more likely to report restriction of social participation, as compared to all other groups; were nearly twice as likely to report serious psychological distress in the past 30 days; and Were nearly twice as likely as Non-Hispanics and nearly three times as likely as other Hispanic adults to be unable to work due to health problems.*

**Slide 24:**

Read the slide.

*Risk factors of Puerto Ricans in the mainland include*

*Language literacy:*

- *61.9% speak a language other than English at home.*
- *17.4% do not speak English “very well”.*

*Poverty/Employment Status*

- *26.2% live below the poverty line.*
- *8% are unemployed.*

*Cigarette smoking*

- *Higher among Puerto Rican males than whites or other Hispanic groups.*
- *More than three times more mainland Puerto Rican mothers reported smoking while pregnant as compared to the total Hispanic population.*

**Slide 25:**

*As compared to other Hispanic populations, mainland Puerto Rican women have a higher prevalence of:*

- *Hypertension*
- *Hypercholesterolemia*
- *Obesity*

*As compared to other Hispanic populations, mainland Puerto Ricans have higher rates of:*

- *Diabetes*
- *Heart Disease*
- *Asthma- children and adults with a prevalence of almost twice as the rest of the Hispanic population.*
- *Alcohol Consumption*

**Slide 26:**

*In 2017 the Top Ten Leading Causes of Death for Non-Hispanic whites and Hispanics were similar, except for Influenza and Pneumonia, for Non-Hispanic Whites, and Chronic Liver Disease-Cirrhosis, for Hispanics.*

*Overall Hispanic mortality is less than for Non-Hispanic Whites, but from the Hispanic population, mainland Puerto Ricans have a higher mean of deaths from all causes combined.*

*Paradoxically, mainland Puerto Ricans are more likely to have health insurance (private or public) than other Hispanic populations, Non-Hispanic Whites and Non-Hispanic Blacks.*

**Slide 27:**

*Specifically, mainland Puerto Ricans, as compared to other Hispanics, have a higher rate of mortality from:*

- *Cancer*
- *Diseases of the heart*
- *Unintentional injuries*
- *Alzheimer’s*
- *Influenza*
- *Homicide*
- *Infant Mortality (Conditions in the Perinatal Period)*
- *Septicemia*
- *Asthma*
- *Diabetes: in fact, Mainland Puerto Ricans and Mexicans are about twice as likely to die from diabetes than whites.*

**Slide 28:**

Transition slide.

*Let’s use Puerto Ricans from New York City as an example.*

**Slide 29:**

*The Department of Health and Mental Hygiene of NYC published the report Health of Latinos in New York City. From the 8.4 million of the total population of New York City, 2.4 million people identify as Latino; 30% of whom identify as Puerto Ricans*

*53% of Puerto Ricans in New York City live below the 200% of the Federal Poverty Level – which is a marker used to determine eligibility for programs such as Medicaid, CHIP, SNAP, Rent Assistance, etc. and 32% have less than a high school education.*

**Slide 30:**

*Puerto Ricans have poorer health than other New Yorkers in New York City.*

*They have higher rates of Smoking, in fact 1 out of 4 smokes tobacco, Childhood Obesity, Asthma, Intimate Partner Violence, Infant death, Premature death (dying before 65 y/o), and unintentional drug overdose.*

*They also report higher rates of serious psychological distress than other Hispanic populations in New York. All of these issues are important to address in primary care settings but can and will impact inpatient care as well.*

**Slide 31:**

Transition slide.

Note: the workshop was implemented by discussing cases in a large group as will be described in this Facilitator’s Guide. The facilitator may use one or more cases.

**Alternative: the group may be divided into small groups and each will discuss one case. Please instruct the attendants to divide into small groups and specify the time allotted to discuss cases in the small group and present answers to questions to the large group. The workshop organizers must print out cases with guided questions (contained in slides) to give out to participants. Also, organizers should printout each case discussion guide contained in this Facilitator’s Guide and give it to the small group facilitators. Each small group should have one facilitator knowledgeable in the topic. If this alternative is chosen, then we recommend that 20 minutes for discussion in small- groups and 10 minutes for each group oral presentation be provided.*

**Slide 32:**

*For each case, please reflect on the following:*

- *The health problems of the patient.*
- *The risk factors contributing to the health problems.*
- *Any problems with access to health care.*
- *Different ways to improve the health care the patient is receiving.*

**Slide 33-35:**

Read the slides

Jose’s Case.

Alternative: if discussing in small groups, we suggest the content of these slides be copied to a Word Document titled: Jose’s Case provide it to each small group participant.

**Slide 36:**Jose’s Case Discussion Guide

Facilitator will lead the discussion and reflection session by directing questions to the attendants and engaging them to discuss and reflect on each question. We suggest each question be discussed for 1-2 minutes.

The questions with the recommended discussion points/answers are as follows:

| **Discussion/Reflection Questions** | **Recommended Discussion Points/Answers** |
| --- | --- |
| *What health problems does José presents?* | - *Asthma* - *Substance Abuse* - *Pneumocystis Pneumonia* - *Possible HIV/AIDS* |
| *What were the risk factors contributing to these problems?* | - *Asthma Genetic Predisposition.^16-17^* - *Socioeconomic factors including poverty, and maybe homelessness.* |
| *Can you identify any problems José may have with access to health care?* | - *He has no health insurance as Medicaid Coverage is up to 26 years of age.* - *He has no primary doctor.* - *At first hospitalization he was not referred to a Social Worker or to a needle exchange program.* |
| *What do you think could be done to improve the health care José is receiving?* | - *Education in asthma management and needle exchange program.* - *Referral to a substance abuse treatment program.* - *Test for HIV/AIDS, other STD/s, and educate about transmission.* - *Assign a Social Worker to work with health insurance and access to treatment programs.* |

Alternative: if discussing in small groups, we suggest this discussion guide be copied into a word document titled Jose’s Case Discussion Guide and provide it to small group facilitators.

**Slides 37-40:**

Read the slides

Jesenia’s Case.

Alternative: if discussing in small groups, we suggest the content of these slides be copied to a Word Document titled: Jesenia’s Case provide it to each small group participant.

**Slide 41:**

Jesenia’s Case Discussion Guide

Facilitator will lead the discussion and reflection session by directing questions to the attendants and engaging them to discuss and reflect on each question. We suggest each question be discussed for 1-2 minutes.

The questions with the recommended discussion points/answers are as follows:

| **Discussion/Reflection Questions** | **Recommended Discussion Points/Answers** |
| --- | --- |
| *What health problems does Jesenia presents?* | - *Obesity* - *HBP- may be due to how anxious she is, weight related, or poverty related.* - *Anxiety* - *Probable Depression* - *Stress-induced mood disorder secondary to a natural disaster leading to exacerbated anxiety and possible PTSD.* |
| *What were the risk factors contributing to these problems?* | - *Poorly controlled anxiety due to lack of treatment.* - *Natural disaster.* - *Death in her spouse.* - *Sudden migration.* |
| *Can you identify any problems Jesenia may have with access to health care?* | - *In PR no access to a Psychiatrist.* - *In the mainland, problems with accessing care due to language barriers.* - *Difficulty navigating a new health care system.* - *Unknown if Medicaid will cover her health care as everyone is a Citizen but coverage is different for Puerto Rico.* |
| *What do you think could be done to improve the health care Jesenia is receiving?* | - *Screen and address for mental health disorders, e.g., Major Depression, Generalized Anxiety Disorder, and PTSD.* - *Educate about weight control including healthy diet and exercise.* - *Try to get a Social Worker or a Community Health Worker (Promotora de Salud) involved in the case to help her get adequate health insurance coverage, a primary physician, and low cost or free psychiatrist evaluation.* |

Alternative: if discussing in small groups, we suggest this discussion guide be copied into a word document titled Jesenia’s Case Discussion Guide and provide it to small group facilitators.

**Slides 42-44:**

Read the slides

Eugenio’s Case.

Alternative: if discussing in small groups, we suggest the content of these slides be copied to a Word Document titled: Eugenio’s Case and provide it to each small group participant.

**Slide 45:**

Eugenio’s Case Discussion Guide

Facilitator will lead the discussion and reflection session by directing questions to the attendants and engaging them to discuss and reflect on each question. We suggest each question be discussed for 1-2 minutes.

The questions with the recommended discussion points/answers are as follows:

| **Discussion/Reflection Questions** | **Recommended Discussion Points/Answers** |
| --- | --- |
| *What health problems does Eugenio presents?* | - *Uncontrolled hypertension* - *Hemorrhagic Stroke* - *Hemiparesis of Left arm and leg* |
| *What were the risk factors contributing to these problems?* | - *High-fat Diet* - *Access to a nutritious diet due to poverty* - *Sedentary Lifestyle* - *Poor compliance with treatment* - *Poverty, underinsured* |
| *Can you identify any problems Eugenio may have with access to health care?* | - *Access to primary care physicians due to rural living and lack of transportation.* - *Access to medication due to poverty.* - *Access to specialized care for his HBP.* - *Access to rehabilitation if he decides to forego it due to his wife’s transportation issues.* |
| *What do you think could be done to improve the health care Eugenio is receiving?* | - *Improve education regarding his health problems.* - *Address risk behaviors and begin an education program to improve diet and include regular exercise.* - *Assign a social worker to help with transportation problems for regular appointments and for wife to be able to visit him while in rehabilitation.* - *Equitable federal funding for Medicaid & Medicare in Puerto Rico.* |

Alternative: if discussing in small groups, we suggest this discussion guide be copied into a word document titled Eugenio’s Case Discussion Guide and provide it to small group facilitators.

Alternative: when discussing in small groups, another alternative is to provide time so that a representative from each small group discusses with the large the group their answers. If this is done, then time to read each case out loud or by reading the case’s slides, must be provided and accounted for.

**Slide 46:**

Transition slide

*As a final point, lets enumerate some recommendations for improving the healthcare of Puerto Ricans.*

**Slide 47:**

*Doctors and other healthcare professionals can engage in culturally targeted approaches such as:*

*Implement a patient-centered, bio-psycho-social-cultural model for taking care of patients.*

*Take the time to develop a cultural formulation, which includes a consideration for acculturation, community and family connection, immigration status/ history, and education.*

*Eliminating language barriers by providing printed educational materials in Spanish, working with interpreters or Spanish speaking professionals thus the importance of teaching Medical Spanish at medical schools.*

*Asking about recent migration and address the stress related to it.*

*Screening for asthma, HIV, and mental health disorders.*

*Providing mental health counseling and treatment when patients meets diagnostic criteria.*

**Slide 48:**

*Engaging women in early prenatal care.*

*Counseling patients on high risk behavior including smoking cessation, alcohol consumption, weight control and diet, substance abuse, and use of birth control.*

*Working with community health workers (Promotoras de Salud) so they can educate the population about available services, health education, and screening. If you want to know more about the community health workers programs, we will provide some resources in the next slide.*

*Instituting a medical home to provide patient-centered, coordinated, and enhanced access to health care.*

*Implementing quality improvement projects to identify and address health disparities within your Hispanic patient population such as, interviewing patients from different Hispanic groups in focus groups to ask them about specific situations accessing the care they need.*

*Implement community based participatory research in which community members are involved in identifying their health problems, developing and implementing a research plan and once outcomes are available, present them to the community at large. Based on these research outcomes, the community develops action plans to address the problems.*

**Slides 49-51:**

*Here are some resources you can use to aid you in providing competent health care to the Puerto Rican population and other Hispanic populations including material to print in Spanish, health intervention recommendations, participatory and community research descriptions, and Puerto Rican history and identity formation.*

Alternative: workshop organizers may choose to copy these two slides into a word document and provide it to participants as a list of resource materials.

**Slide 52:**

*Thanks for your attention and participation. If there are any questions?*

**References:**

- 1. Dominguez K, Penman-Aguilar A, Chang M, et al. Vital Signs: Leading Causes of Death, Prevalence of Diseases and Risk Factors, and Use of Health Services Among Hispanics in the United States — 2009–2013. *Morbidity and Mortality Weekly Report*. 2015;64(17):469-478.
  2. Fernández-Armesto Felipe. *Our America: a Hispanic History of the United States*. New York: W.W. Norton & Company; 2015.
  3. Immerwahr Daniel. *How to Hide an Empire: a History of the Greater United States*. S.l.: PICADOR; 2020.
  4. Puerto Rico - History and Heritage. Smithsonian.com. https://www.smithsonianmag.com/travel/puerto-rico-history-and-heritage-13990189/. Published November 6, 2007. Accessed August 12, 2019.
  5. Kishore N, Marques D, Mahmud A, et al. Mortality in Puerto Rico after Hurricane Maria. *New England Journal of Medicine*. 2018;379(17):162-170. doi:10.1056/nejmc1810872.
  6. Willison CE, Singer PM, Creary MS, Greer SL. Quantifying inequities in US federal response to hurricane disaster in Texas and Florida compared with Puerto Rico. *BMJ Global Health*. 2019;4(1). doi:10.1136/bmjgh-2018-001191.
  7. Mora M, Dávila A, Rodriguez H. Migration, Geographic Destinations, and Socioeconomic Outcomes of Puerto Ricans during La Crisis Boricua: Implications for Island and Stateside Communities Post-Maria. *Centro Journal*. 2018;30(3):208-229.
  8. Sanchez Korrol V. The Story of U.S. Puerto Ricans - Part Four. Centro de Estudios Puertorriqueños. http://centroweb.hunter.cuny.edu/education/puerto-rican-studies/story-us-puerto-ricans-part-four. Published 2016. Accessed August 8, 2019.
  9. Santiago CE. The Migratory Impact of Minimum Wage Legislation: Puerto Rico, 1970-1987. *International Migration Review*. 1993;27(4):772. doi:10.2307/2546912.
  10. Minimum Wage - Wage and Hour Division (WHD) - U.S. Department of Labor. History of Changes to the Minimum Wage Law. https://www.dol.gov/whd/minwage/coverage.htm. Accessed August 12, 2019.
  11. Mora MT, Dávila A, Rodríguez H. Education, migration, and earnings of Puerto Ricans on the island and US mainland: Impact, outcomes, and consequences of an economic crisis. *Migration Studies*. 2016;5(2):168-189. doi:10.1093/migration/mnw032.
  12. Puerto Rico Post Maria Report. Centro de Estudios Puertorriqueños. https://centropr.hunter.cuny.edu/events-news/rebuild-puerto-rico/puerto-rico-post-maria-report. Published 2018. Accessed August 8, 2019.
  13. Sanchez Korrol V. The Story of U.S. Puerto Ricans - Part Four. Centro de Estudios Puertorriqueños. http://centroweb.hunter.cuny.edu/education/puerto-rican-studies/story-us-puerto-ricans-part-four. Published 2016. Accessed August 8, 2019.
  14. Ley Núm. ^14^ del año 2017. Lexisjuris.com. http://www.lexjuris.com/lexlex/Leyes2017/lexl2017014.htm. Published 2017. Accessed August 11, 2019.
  15. Michaud J, Kates J. Public Health in Puerto Rico after Hurricane Maria. The Henry J. Kaiser Family Foundation. https://www.kff.org/other/issue-brief/public-health-in-puerto-rico-after-hurricane-maria/. Published November 20, 2017. Accessed August 9, 2019.
  16. Ober C, Yao T-C. The genetics of asthma and allergic disease: a 21st century perspective. Immunological Reviews. 2011;242(1):10-30. doi:10.1111/j.1600-065x.2011.01029.x.).
  17. Celedon J. Asthma: More Than Just Genetics. National Institute of Minority Health and Health Disparities. <https://nimhd.nih.gov/news-events/features/biological-behavioral-siences/asthma.html>. Published May 20, 2029. Accessed April 1, 2020.
