# Supplementary material for: Taking Care of the Puerto Rican Patient: Historical Perspectives, Health Status, and Health Care Access
Source: MedEdPORTAL. 2020 Oct 7;16:10984. doi: 10.15766/mep_2374-8265.10984 (PMC7549386; doi:10.15766/mep_2374-8265.10984)
Supplement: Supplementary file 1 — Taking Care of the Puerto Rican Patient.pptxFacilitator Guide.docxEvaluation Forms.docx [file mep_2374-8265.10984-s001.zip › C. Evaluation Forms.docx]

**Taking Care of the Puerto Rican Patient: Historical Perspectives, Health Status, and Health Care Access**

**Pre-Test**

**Part I. Demographic Information**

**Select ONE circle that describes your current professional role:**

- Medical Student
- Medical Resident or Fellow
- Academic Faculty (MD, DO, NP, PA, Other)
- Clinician in Non-Academic Practice (MD, DO, NP, PA, Other)
- Other _________________

**Please mark all that apply:**

- Female
- Male
- Transgender or Gender Nonconforming
- Different Identity

**Please mark all that apply:**

- Latino/Hispanic
- Puerto Rican
- Black/African American
- White
- Asian
- Native American/Alaskan or Pacific Islander
- Other ________________

**Part II. Self-Assessment**

| **Please rate how much CONFIDENCE do you have in your ability to…** | **No**  **Confidence**  **0** | **1** | **2** | **3** | **Complete**  **Confidence**  **4** |
| --- | --- | --- | --- | --- | --- |
| Obj 1: Describe the history of Puerto Rican identity within the United States | 0 | 1 | 2 | 3 | 4 |
| Obj 2: Compare and contrast health issues and disparities of Puerto Ricans residing on the island and on the mainland | 0 | 1 | 2 | 3 | 4 |
| Obj 3: Explain how, at least, one federal policy or decision has impacted the health outcomes of Puerto Ricans on the mainland and/or the island. | 0 | 1 | 2 | 3 | 4 |
| Obj 4: Identify, at least, two health care access problems faced by Puerto Ricans on the mainland and/or the island. | 0 | 1 | 2 | 3 | 4 |

**Part III. Objective Assessment**

**Please Choose the Best Answer:**

1. Puerto Ricans are American citizens through…
   1. Naturalization
   2. Birthright
   3. International treaty
   4. United Nations resolution
2. Which of the following set the precedent for unequal federal funding for Medicare and Medicaid in Puerto Rico?
   1. Jones-Shafroth Act
   2. Medicare/Medicaid Act
   3. Insular Cases
   4. Affordable Care Act
3. Compared to other Hispanic and Non-Hispanic adults, Puerto Ricans in the mainland have higher prevalence of:
   1. Malnutrition
   2. Hypothyroidism
   3. Asthma
   4. Chronic Liver Disease-Cirrhosis

**Taking Care of the Puerto Rican Patient: Historical Perspectives, Health Status, and Health Care Access**

**Post-Test**

**Part I. Self-Assessment**

| **Please rate how much CONFIDENCE do you have in your ability to…** | **No**  **Confidence**  **0** | **1** | **2** | **3** | **Complete**  **Confidence**  **4** |
| --- | --- | --- | --- | --- | --- |
| Obj 1: Describe the history of Puerto Rican identity within the United States | 0 | 1 | 2 | 3 | 4 |
| Obj 2: Compare and contrast health issues and disparities of Puerto Ricans residing on the island and on the mainland | 0 | 1 | 2 | 3 | 4 |
| Obj 3: Explain how, at least, one federal policy or decision has impacted the health outcomes of Puerto Ricans on the mainland and/or the island. | 0 | 1 | 2 | 3 | 4 |
| Obj 4: Identify, at least, two health care access problems faced by Puerto Ricans on the mainland and/or the island. | 0 | 1 | 2 | 3 | 4 |

**Part II. Objective Assessment**

**Please Choose the Best Answer:**

1. Puerto Ricans are American citizens through…
   1. Naturalization
   2. Birthright
   3. International treaty
   4. United Nations resolution
2. Which of the following set the precedent for unequal federal funding for Medicare and Medicaid in Puerto Rico?
   1. Jones-Shafroth Act
   2. Medicare/Medicaid Act
   3. Insular Cases
   4. Affordable Care Act
3. Compared to other Hispanic and Non-Hispanic adults, Puerto Ricans in the mainland have higher prevalence of:
   1. Malnutrition
   2. Hypothyroidism
   3. Asthma
   4. Chronic Liver Disease-Cirrhosis

**Part III. Comments**

**Please answer the following questions:**

1. What did you like about this workshop?
2. What suggestions do you have to improve this workshop?
